# Advances in molecular pathology and therapy of non-small cell lung cancer

**DOI:** 10.1038/s41392-025-02243-6

**Published:** 2025-06-15

**Authors:** Qing Huang, Yuanxiang Li, Yingdan Huang, Jingyi Wu, Wendai Bao, Chang Xue, Xiaoyu Li, Shuang Dong, Zhiqiang Dong, Sheng Hu

**Affiliations:** 1 Department of Medical Oncology, Huazhong University of Science and Technology, Tongji Medical College, Hubei Cancer Hospital, Wuhan, 430079 Hubei China; 2 Center for Neurological Disease Research, Taihe Hospital, Hubei University of Medicine, Shiyan, 442000 Hubei China

**Keywords:** Lung cancer, Cancer

## Abstract

Over the past two decades, non-small cell lung cancer (NSCLC) has witnessed encouraging advancements in basic and clinical research. However, substantial unmet needs remain for patients worldwide, as drug resistance persists as an inevitable reality. Meanwhile, the journey towards amplifying the breadth and depth of the therapeutic effect requires comprehending and integrating diverse and profound progress. In this review, therefore, we aim to comprehensively present such progress that spans the various aspects of molecular pathology, encompassing elucidations of metastatic mechanisms, identification of therapeutic targets, and dissection of spatial omics. Additionally, we also highlight the numerous small molecule and antibody drugs, encompassing their application alone or in combination, across later-line, frontline, neoadjuvant or adjuvant settings. Then, we elaborate on drug resistance mechanisms, mainly involving targeted therapies and immunotherapies, revealed by our proposed theoretical models to clarify interactions between cancer cells and a variety of non-malignant cells, as well as almost all the biological regulatory pathways. Finally, we outline mechanistic perspectives to pursue innovative treatments of NSCLC, through leveraging artificial intelligence to incorporate the latest insights into the design of finely-tuned, biomarker-driven combination strategies. This review not only provides an overview of the various strategies of how to reshape available armamentarium, but also illustrates an example of clinical translation of how to develop novel targeted drugs, to revolutionize therapeutic landscape for NSCLC.

## Introduction

**Fig. 1:**
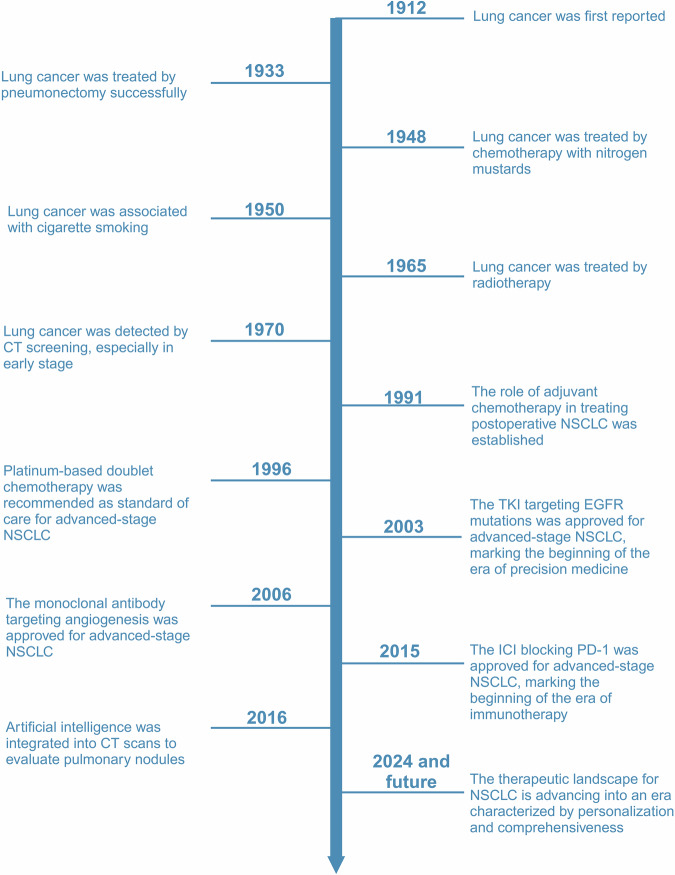
Key milestone events in lung cancer research. The illustration provides 11 significant advances in the diagnosis and treatment of lung cancer, highlighting the most rapid developments over the past 20 years and projecting future directions

Lung cancer is a relatively recent addition to the medical field, being almost unknown until just over a century ago, with mere 374 confirmed cases documented worldwide by Adler’s report in 1912.^1^ Unfortunately, today, the global burden of lung cancer which includes NSCLC (near to 85%, accordingly focused by this article) and small cell lung cancer (SCLC), has become a paramount societal, public health, and economic problem, because of its catastrophic prevalence with around 2.5 million new cases and more than 1.8 million deaths in 2022, ranking first in both sexes and all ages.^2^ Similarly, in China, the numbers are 4,824,703 and 2,574,176, respectively. Since 2006, owing to significant improvements in screening and diagnostic techniques, high-precision radiotherapy (RT) and surgical operations, and novel targeted therapies and immunotherapies according to biomarkers (Fig. 1),^3^ the incidence of NSCLC has decreased annually by 2.5% in males and 1% in females in some countries with very high Human Development Index (HDI), along with the mortality rates. Nevertheless, the five-year survival from NSCLC still tends to be below 20% in most countries, with little difference according to HDI.^2^

Consequently, investing in preventive measures, such as targeting key risk factors for cancer (e.g., smoking, overweight obesity, and a legacy of human behavior resulting in complex environmental exposures),^4^ and utilizing the latest technological tools to delve deeply into the occurrence, development, and metastasis mechanisms of NSCLC is a high-priority essential. This will allow us to translate these findings into highly effective, low-toxicity drugs and precise treatment strategies, ultimately holding the potential to save a multitude of lives affected by NSCLC globally. While the upfront costs may appear daunting in the short term, from a long-term macro-perspective, the substantial net economic and social benefits to countries over the next few decades cannot be ignored.^5^

Therefore, this review offers a holistic perspective into the epidemiological features of NSCLC, the multi-dimensional dynamics in cancer cell or non-malignant cell phenotypic characteristics, and strategies on how to optimize multifaceted therapeutic approaches tailored to different stages of NSCLC powered by diverse biomarkers. We also explored the intricate interplay between host and tumor fostering drug resistance, and then discussed how to overcome resistance through mechanism-driven combinatorial therapy methods. Lastly, we proposed how to accelerate the translation of novel drugs by leveraging various platforms and technologies grounded in big-data-driven artificial intelligence (AI) algorithms. More critically, we underscored the necessity of judiciously harnessing real-world data in selecting the Goldilocks treatment, especially given the pressing time constraints faced by patients waiting for randomized controlled trials to commence. Alternatively, a more nuanced approach would be to enable patients to manage cancer as a chronic, minimally symptomatic condition, which could significantly enhance their quality of life, considering that the total eradication of cancer, including NSCLC, might not always be achievable.

However, the achievement of this goal faces enormous challenges characterized by the daunting diversity, plasticity, and neo-Darwinian adaptability of NSCLC in the breadth and scope of (epi)genetics, cell and tissue biology, pathology, and therapeutic response, as well as the various logistical and economic barriers faced by patients and their families, also by governments.^6^ Optimistically, no matter the obstacles, we firmly believe that within the next two decades, an entirely new landscape of NSCLC treatments will unfold before our eyes (Fig. 1^1,7–17^).

## NSCLC global epidemiology

Globally, lung cancer, mainly NSCLC, leads in cancer incidence and mortality and primarily occurs in individuals over 65 years of age, with only a small percentage of cases diagnosed in individuals younger than 45 years. The increase in incidence with age can be attributed to various factors, including the accumulation of chronic genetic damage, epigenetic drift, alterations in tissue microenvironments, and dysfunction in adaptive and innate immunity.^18^ The epidemiology of lung cancer exhibits significant heterogeneity and dynamics related to region, gender, smoking status, and socio-economic factors. According to regions, North America, East Asia, and Northern Europe have higher incidences of lung cancer, with Hungary having the highest incidence rate. The American Cancer Society predicts approximately 234,580 new cases and 125,070 deaths from lung cancer in the US in 2024. In comparison, unfortunately, one-third of total lung cancer cases occur in China in 2022, with an estimated 1,060,584 new cases and 733,291 deaths due to the large population base.

Among men worldwide, East Asia has the highest incidence of lung cancer, followed by Micronesia/Polynesia and Eastern Europe, with Turkey leading the rate. Among women, lung cancer is the most common cause of cancer death in 23 countries including China and the United States. Lung cancer ranks first in both incidence and mortality in men, and second in both in women, with a global male-to-female ratio of approximately 2 for incidence and mortality. However, this ratio varies significantly by region, ranging from almost equal in North America and Northern Europe to four to five times higher in North Africa and Eastern Europe. Adenocarcinoma is the most common subtype of lung cancer globally in 2022, with an incidence surpassing squamous cell carcinoma in men in most countries and in women in all 185 countries.

**Fig. 2:**
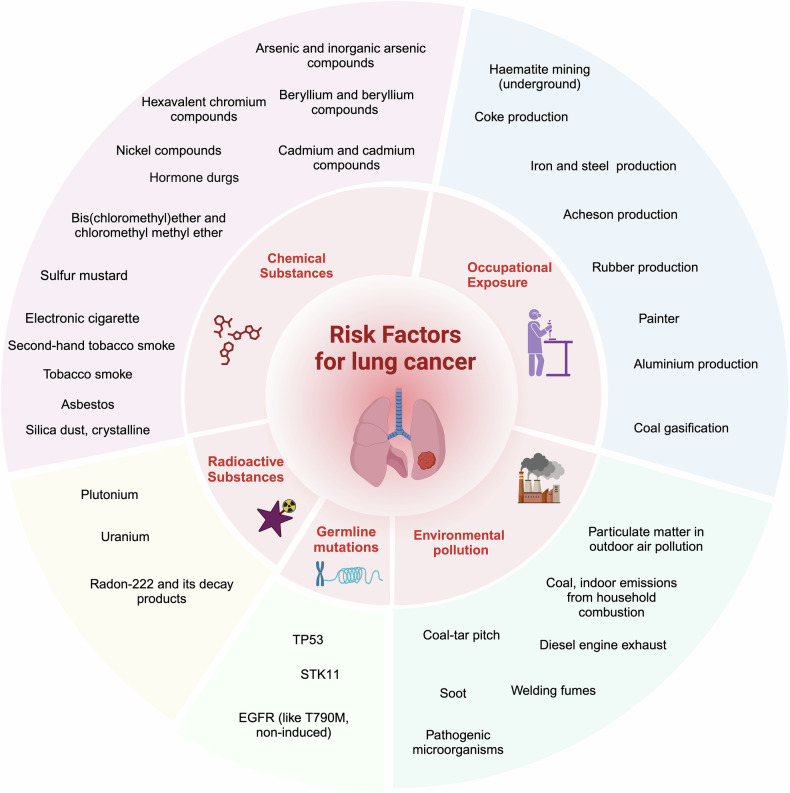
Known carcinogenic causes of lung cancer (cited from the World Cancer Report, Cancer research for cancer prevention, 2020, with modifications and updates). The carcinogenic causes of lung cancer vary by region, race, and gender. Collectively, they, when combined, contribute to the development of lung cancer, including environmental pollution, unhealthy lifestyle habits, and genetic predisposition, though the extent may vary among individuals. Over time, with social progress, the nature of these carcinogenic factors has evolved; for example, infections have increasingly been linked to environmental pollution, and traditional tobacco use has shifted toward electronic cigarettes

As all traditional smoking and smokeless tobacco products are linked to lung cancer, the extent of tobacco use in the countries, as well as their historical differences in tobacco exposure from smoking intensity and duration, types of cigarettes, and degree of inhalation may be primarily reflected in the unique patterns of lung cancer incidence and mortality across geography, gender, and temporality. In addition, other environmental risk factors and genetic susceptibility play significant roles in the development of lung cancer, particularly among non-smoking populations, although the specific dominant factor remains unclear (Fig. 2). Some countries have the highest prevalence of male smokers, such as China, Russia, and Indonesia, which are also the most populous countries in the world. In contrast, there is a huge variation in female smoking rates, with a small proportion of women estimated to smoke daily (<5%) in Indonesia, China, and most African countries. However, globally, about a quarter of lung cancer cases are attributed to causes other than tobacco smoking. In East Asia, where female smoking rates are extremely low, non-smoker lung cancer accounts for a significant proportion of the overall disease burden, partly due to environmental exposures. For example, the high rate of lung cancer (mainly adenocarcinoma) among Chinese women believed to involve an interplay of genetic risks, increased ambient particulate matter, and exposure to solid fuel waste during heating and cooking processes. Moreover, multiple studies have revealed that lung cancer in non-smokers differs on a genomic and molecular level from smoking-related lung cancer, characterized by an enrichment of targetable oncogenic alterations (such as EGFR mutations). With the reduction in tobacco consumption, adenocarcinoma may eventually become the most common form of lung cancer in the future, underscoring the need for a comprehensive understanding of its pathogenesis to devise effective preventative and therapeutic measures against this growing concern.^19^

In addition to geographical and gender differences, the epidemiological characteristics of lung cancer are also associated with various significant social and macroeconomic costs. Therefore, according to the United Nations Development Program’s Human Development Report 2021–2212, lung cancer burden is classified according to predefined low, medium, high and very high HDI levels. From a global perspective, as the HDI level increases, the risk of lung cancer tends to increase. In countries with high HDI levels (including China), lung cancer is the most common type of cancer (7,436,122 new cases and 3,991,272 deaths), however, female breast cancer is the most prevalent form in terms of incidence in higher HDI-level countries except for China. When classified by income, lung cancer exhibits similar epidemiological characteristics, remaining the leading cause of incidence and mortality in upper-middle income countries (including China), with 7,811,817 new cases and 4,105,041 deaths, respectively.

Given the late-stage diagnosis of most lung cancers, that makes curative treatment difficult, there has been a longstanding focus on screening high-risk individuals (smokers and former smokers). Randomized controlled trials such as the US National Lung Screening Trial and the NELSON study have shown that low-dose computed tomography (CT) significantly reduces the mortality rate of lung cancer in this population.^20^ However, translating this into benefits for the general population poses significant challenges, taking into account the costs and necessary infrastructure.^21^ In the future, prospective research using deep learning (DL), an artificial intelligence solution, will be utilized to evaluate whether low-dose CT screening can reduce the frequency of false positive, although it may not be cost-effective.^22^ By extension, cancer prevention through reducing tobacco consumption, may have higher cost-effectiveness, saving $7.9 billion annually in China, $402 million in Brazil, and $1.38 billion in South Africa (based on 2012 data).^5^

Looking ahead to the future, based on the demographic assumption of a constant growth rate, the global population is projected to reach 9.7 billion by 2050, with over 35 million new cancer cases expected to occur. This represents a 77% increase from the estimated 20 million cases in 2022. While the absolute differences in cancer burden are greatest in high HDI countries, including China, and very high HDI countries (with expected increases of 4.8 million and 3.9 million cases, respectively, compared to 2022), the highest relative growth rates will be seen in the low and medium HDI countries, including India. The increase is anticipated to be from 800,000 to 2 million cases, and from 2.4 million to 4.8 million cases respectively, as these countries are experiencing significant cancer risk factors known to be prevalent in the former two, including smoking, unhealthy diet, being overweight, and lacking in physical activity.

In the molecular epidemiology of NSCLC, significant racial differences exist, particularly, characterized as Asian women are nearly four times more likely to have EGFR mutations compared to Caucasian women, although there are some variations in the specific subtypes of the mutations. On the other hand, KRAS mutations are less frequently observed in Asian patients (8–10% compared to 20–30%), with a prevalence of KRAS^G12C^ mutations at 1.5–4.3%, which is significantly lower than the 10–15% observed in Caucasians.^23^ Differences in other mutations are less pronounced.^24^ Light or never smokers and younger patients are more likely to have EGFR mutations and fusions such as ROS-1, ALK, and RET,^25^ whereas heavy smokers are more likely to have KRAS, MAP2K1, and TP53 mutations. Moreover, disease progression or drug stress can lead to the loss or acquisition of various genetic variants. However, at present, the relationship between the epidemiology of epigenetic changes and racial, regional, and environmental factors remains unclear in NSCLC, although epigenetic changes are also potential targets for treatment.

The International Agency for Research on Cancer (IARC) provides valuable interpretations of the global cancer burden and characteristics every two years, however the reports need to be carefully interpreted since many low and middle-income countries lack high-quality registration data on incidence and mortality rate. Furthermore, the COVID-19 pandemic in 2019 has had a significant impact on cancer data registration globally, with estimates provided in 2022 not reflecting the pandemic’s effects as they are primarily extrapolated from cancer data collected before 2020. Nevertheless, some progress has been made in compiling the 2022 estimates of cancer incidence by integrating data from various sources, such as utilizing data from 700 cancer registration centers in China and the SurvCan-3 project, as well as from the collaboration with the European Commission’s Joint Research Centre and the European network of cancer registries. In summary, while we have shown the overall burden of lung cancer, comprehensive and in-depth research that covers the exploration of pathological mechanisms, clinical validations, and practical applications is essential for planning, implementing and monitoring the effectiveness of national or regional cancer control programs (canceratlas.cancer.org).

## Advances in preclinical research related to molecular pathology in NSCLC

The development of NSCLC, including lung adenocarcinomas (LUAD) and lung squamous cell carcinoma (LUSC), is driven by heterogeneous genetic and epigenetic alterations, which are multi-step and complex processes involving various signaling crosstalk among distinct pathways. Currently, preclinical studies mainly focus on elucidating the molecular mechanisms underlying the origin, development, and metastasis of NSCLC, providing essential theoretical foundations for cancer prevention, identification of new biomarkers, discovery of new therapies and optimization of treatment strategies. With advances in single-cell sequencing technology and spatial multi-omics analysis, we can gain a deeper understanding of the heterogeneity and complexity of tumor progression, as well as analyze the crosstalk between cancer cells and the tumor microenvironment (TME), giving rise to new perspectives for identifying therapeutic targets that may halt or possibly eliminate cancer growth and metastasis.

Molecularly, LUSC is characterized by a high rate of genetic mutations and chromosomal instability, however, there are some special mutations that are enriched in certain subsets of patients.^26^ However, the common driver mutations found in LUAD are rarely identified in LUSC and efforts at identifying driver mutations in LUSC have not been fruitful.^27^ Common patterns of chromosomal aberrations in LUSC can be grouped into several categories, including upregulation of squamous cell differentiation pathways (NOTCH, SOX2 and TP63), loss of cell cycle regulation (TP53, RB1, CDKN2A, MYC and SMARCB1), upregulation of oncogenic signaling through the RAS and PI3K pathways, and abnormalities in epigenetic regulators (KMT2D, NSD1 and KDM6A).^26^

However, in this article, we highlighted LUAD, the most common histological entity in NSCLC, into which precision oncology has taken a lead in pioneering. Growth patterns, categorized as lepidic (low-grade), papillary and acinar (mid-grade), and cribriform, micropapillary, and solid (high-grade), are frequently mixed within a single LUAD tumor, and the proportion of high-grade patterns within each tumor is known to impact patient outcome,^28^ although their potential genomic underpinnings, are still poorly understood.

### Mechanisms that regulate tumorigenesis

**Fig. 3:**
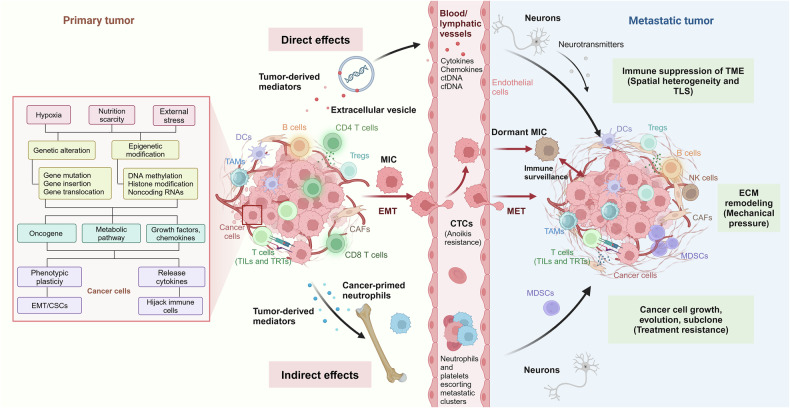
Mechanisms involved in cancer cell metastasis in NSCLC. This schematic diagram summarizes the mechanisms involved in cancer cell metastasis in NSCLC. From an evolutionary perspective, metastasis represents a systemic adaptive response to stress within the survival microenvironment. The process begins with several changes in cancer cell characteristics, at least including the activation of oncogenes (e.g., EGFR, ALK, and RAS, etc.), upregulation of cytokines (e.g., IL-6, TGF, IGF, and HIF-1, etc.), chemokines, and their receptors (e.g., CXCL9, CXCL10, CXCL11, CXCR3, CXCR4, and CCR5, etc.), as well as metabolic reprogramming, as detailed in the box. This is followed by phenotypic transformation involving EMT, primarily driven by miRNAs, ZEB1/2, and EZH2. Subsequently, through various soluble molecules and exosomes, the microenvironment of distant organs is altered to support the survival of cancer cells(direct effect). Cancer cells also reeducate bone marrow-derived immune cells, creating an immunosuppressive microenvironment (indirect effect). Additionally, when circulating in the bloodstream, cancer cells co-opt neutrophils and platelets to resist anoikis, and subsequently upregulate adhesion molecules on endothelial cells, facilitating their entry into tissues. In the pre-metastatic niche of distant organs, restoration of MET, ECM remodeling (by MMP2, 9, and 10), induction of angiogenesis (via VEGF, VEGFR, TIE2, and angiopoietin 1/2), and recruitment of CAFs and neuronal cells (via GABA) occur, alongside the engagement of immunosuppressive cells (MDSCs, T_reg_s, tumor-promoting TANs and TAMs). These cells assist in inducing T cell exhaustion (via PD-(L)1, CTLA-4, LAG3, and TIM3, etc.) and metabolic competition (through upregulation of glutaminases GLS1 and GLS2, and accumulation of toxic cancerous metabolites), enabling the evasion of surveillance by TILs and tissue-resident T cells (αβ or γδ T cells), NK cells, macrophages, and B cells. This complex interplay allows for survival, subclonal evolution, and therapeutic resistance. Without these mechanisms, the tumor may remain dormant for over 10 years before manifesting clinically in distant organs. Note that the pathways and molecules listed do not cover the entire spectrum of the metastatic process, nor do they fully explain the organ-specific nature of metastasis or provide insights into how these contribute to therapeutic resistance

Cancer cells originate from defects within pre-existing cells such as increased proliferation, genetic susceptibility, as well as external stress and various carcinogenic factors. Smoke exposure can lead to a well-defined series of morphological changes of the bronchial epithelium progressing from basal cell hyperplasia to metaplasia, severe dysplasia to carcinoma in situ and, finally, frank carcinoma. This series of changes is primarily associated with LUSC, which may be associated with potential advantages in terms of immunotherapy benefits. By contrast, adenocarcinomas can also arise in the context of heavy carcinogen exposure and underlying lung damage, but they are generally considered to be the dominant subtype in never-smokers with low carcinogen exposure.^29^ Mechanistically, the tumorigenesis of NSCLC is driven by multiple pathways which are involved in genetic variants in germline or non-germline cells^30^ and epigenetics variants^31^ (Fig. 3). Till now, most studies have focused on the various genomic, proteomic, metabolomic, and immunological characteristics and interactions involved in cancer formation and on how to target them. Fundamentally, the transformation of cancer cells into normal cells has not been unsuccessful.

#### Driver mutations of NSCLC

The identification of driver mutations has been pivotal in understanding the molecular mechanisms underlying the tumorigenesis of NSCLC (the vast majority occurring in LUAD). Recent technological advances in genomic analysis have revealed numerous genetic alterations involving key pathway components in receptor tyrosine kinase signaling, mTOR signaling, oxidative stress response, proliferation and cell cycle progression, which lead to oncogenic transformation in this disease.^31^ We primarily focus on driver gene variations (also known as oncogene addiction) in NSCLC, such as KRAS, EGFR, anaplastic lymphoma kinase (ALK), ROS1, BRAF, HER-2, MET, NTRK and RET mutations, as these can serve as therapeutic targets. However, inactivating mutations in other tumor related genes, such as TP53, KEAP1, STK11, and NF1, are also important.^32,33^

##### EGFR mutant

EGFR is a 170 kDa protein that belongs to a family of receptor tyrosine kinases, which is stimulated by cognate ligands, such as EGF, amphiregulin and transforming growth factor-α (TGF-α), or non-cognate ligands, such as betacellulin, heparin-binding EGF and epiregulin, triggering a cascade of intracellular signaling that promotes cellular survival, proliferation and migration, but restrains apoptosis, and even indirectly induces angiogenesis within TME. In tumor cells, EGFR tyrosine kinases are activated by various mechanisms, including mutation, overexpression, and autocrine or paracrine production of EGF family ligands. Therefore, they, especially mutations, can serve as therapeutic targets.^34^ In most advanced NSCLC patients, EGFR mutations primarily consist of exon 19 deletions (accounting for 45%) and the L858R mutation (accounting for 40%), either alone or in combination with other mutations.^35^ These mutations are particularly prevalent among Asian women with adenocarcinoma who have either never smoked or have a history of mild smoking. However, in Caucasian NSCLC patients, these mutations account for only about 10%. EGFR mutations can indeed occur in adenosquamous carcinomas and in a minority of pure squamous cell carcinoma patients,^36^ although their response to tyrosine kinase inhibitors (TKIs) is typically shorter compared to adenocarcinoma patients.^37^

Exon 20 insertion mutations are the third most common type of EGFR mutations among NSCLC patients, comprising approximately 2% of cases, and frequently coexist with other EGFR mutations at a rate of 4–12%,^38^ among which the most commonly observed insertions are situated at Asp770 (28.7%), Val769 (20.5%), Pro772 (17.2%), and His773 (14.0%).^39^ The EGFR Thr790Met (T790M) mutation is more typical in patients who have acquired resistance to TKI treatments.^40^ This mutation, along with mutations in the STK11 and TP53 genes, is associated with an increased susceptibility to lung cancer, therefore, to test for their germline mutations and simultaneously to provide genetic counseling are strongly recommended.^41^

##### KRAS mutant

KRAS is one of the most commonly mutated oncogenes accounting for approximately 30% of LUAD,^42^ and is also highly mutated in patients with significant tobacco exposure. Mutated KRAS prevents the GTPase activity, blocking RAS proteins away from active GTP, leading to the sustained activation of downstream effector signaling pathways. Approximately 80% of mutations occur in codon 12, which is related to nucleotide-binding of KRAS and effector protein switches. Among them, the most common mutations are KRAS^G12C^ (transformation of glycine to cysteine, approximately 40%), KRAS^G12V^ (glycine to valine, approximately 18–21%), and KRAS^G12D^ (glycine to aspartic acid, approximately 17–18%). Besides codon 12, both codon 13 and 61 are also frequently mutated, like glycine to cysteine (G13C) and glutamine to histidine (Q61H).^43^ In NSCLC, studies have revealed that RAS pathway inhibitors, possibly unlike distinctively from those against other oncogenic pathways, could potentially amplify the immunogenicity of these tumors via a range of mechanisms, including reducing PD-L1 expression, promoting the synthesis of MHC-I molecules, altering the tumor microenvironment to facilitate T cell activity, and escalating the proportion of antitumor macrophages relative to pro-tumor macrophages. Essentially, the partial function of mutation-selective RAS inhibitors lies in stimulating in vivo antitumor adaptive immune responses, as their efficacy tends to be weaker in mouse models lacking T cells.^44^ Usually, KRAS mutations do not coincide with genetic variations in EGFR, ROS1, BRAF and ALK, but they can, albeit rarely, align with RET rearrangements.^45^ Patients with KRAS mutations often have a shorter survival time, historically making KRAS mutation a poor prognostic biomarker, however, with the advent of various targeted therapies and ICIs, this notion may be evolving.^44^

##### ALK translocations (also known as fusions or rearrangements)

ALK rearrangements, primarily involving EML4-ALK fusions but also including KIF5B-ALK, TFG-ALK, and KLC1-ALK among others, are detected in approximately 3–5% of NSCLC cases.^46^ These cases share clinical features with EGFR mutations, such as adenocarcinoma histology and a history of mild or no smoking.^47^ Fusion transcripts containing the ALK kinase domain may promote kinase activation in downstream survival signaling, thereby providing vulnerability to TKI treatment.^48^

##### ROS1 rearrangements

The fusions of ROS1 gene, located on chromosome 6 at region 6q22.1, were first identified in the U118MG glioblastoma cell line in 1987.^49^ Subsequently, in 2007, this gene fusion was observed in younger (with a median age below 50) non-smoking (approximately 80%) LUAD patients, constituting about 1–2% of cases. Typically, ROS1 gene fusions are mutually exclusive with other driver mutations and associated with a higher rate of venous thromboembolism. Despite being an independent receptor tyrosine kinase, ROS1 shares approximately 70% homology with the kinase domain of ALK and consequently can be inhibited by the ALK inhibitor crizotinib.^50^

##### BRAF mutations

BRAF (v-Raf murine sarcoma viral oncogene homolog B), a serine/threonine kinase, is a component of the MAP/ERK signaling pathway. More than 200 BRAF mutations have been identified, predominantly occurring at the 600th codon (where approximately 50% are V600E and the others include V600K, V600D, V600R, and V600M mutations),^51^ which are found in approximately 1–2% of NSCLC, comprising 30–50% of all BRAF mutations. Additional mutations include low-activity BRAF variants that span from position G464 to K601. Such mutations are frequently linked to smoking habits.^52^ BRAF mutations typically do not coincide with EGFR mutations, MET exon 14 skipping mutations, RET rearrangements, ALK rearrangements or ROS1 rearrangements. Although the frequency is lower, mutation testing for BRAF may also be considered in patients with metastatic LUSC.^36^

##### NTRK1/2/3 Fusions

The NTRK (neurotrophic tyrosine receptor kinase) family, including NTRK1, NTRK2, and NTRK3, comprises a group of transmembrane tyrosine kinases that play a critical role in neural development.^53^ In NSCLC, NTRK fusions are rare but recurrent oncogenic drivers, estimated to occur in a range of 0.1–0.2%, and typically do not overlap with other oncogenic drivers such as EGFR, ALK or ROS1. Patients with NSCLC harboring NTRK fusions tend to be younger and have little to no history of smoking.^54^ Additionally, certain tumors have been observed to carry NTRK point mutations, splicing variants, and copy number increases,^55^ yet, whether these alterations can serve as viable treatment targets or correlate with the benefits of accessible targeted therapies, remains unclear.^53^

##### MET Alterations

C-MET (cellular mesenchymal-epithelial transition), a tyrosine kinase receptor, is commonly associated with driving genomic alterations, including MET exon 14 skipping or other kinase domain point mutations,^56^ or gene amplification. The incidence of MET exon14 skipping mutations is around 3–4% in adenocarcinoma,^57^ approximately 1–2% in squamous cell carcinoma, and about 20% in pulmonary blastoma. In NSCLC, MET exon 14 mutation carriers are more common among elderly women (median age 70 years), with a history of more frequent tobacco exposure,^58^ compared to other oncogenic mutations. MET exon 14 mutations coexist with MDM2, CDK4, and MET amplifications at rates of 34%, 19%, and 11%, respectively, and TP53 mutations at 42%,^59^ but are generally mutually exclusive with other oncogenic drivers.

##### RET rearrangements

The proto-oncogene RET, identified in 1985, is a receptor tyrosine kinase (RTK) that, through rearrangements with other regions such as KIF5B (accounting for 70% of RET rearrangements) and CCDC6, can lead to excessive activation of the RET protein. In NSCLC, RET rearrangements occur in approximately 1–2% of cases, particularly more frequently in relatively younger patients (≤60 years old) with poorly differentiated adenocarcinomas and with little to no smoking history, who are typically characterized by low PD-L1 expression levels and a low tumor mutation burden (TMB).^60^ RET rearrangements usually do not overlap with genetic variations in EGFR, ROS1, BRAF, MET exon 14 skipping, and ALK, however, there might be sporadic instances of co-mutations with KRAS.^45,61^

##### ERBB2 (HER-2) mutations

HER-2, a receptor tyrosine kinase found on the surface of normal epithelial cells, acts as a confirmed oncogenic driver mutation characterized by overexpression or mutation in NSCLC, affecting 1.5–3% of patients with a median age of 62 years. The most prevalent mutations in NSCLC involve intronic insertions within exon 20, with the Y772dupYVMA insertion accounting for 68% of all HER-2 exon 20 insertions, followed by G778dupGSP at 14% and G776delinsVC at 9%.^62^ HER-2 exon 20 insertions show an exclusion relationship with EGFR mutations and ALK rearrangements, and they are more prevalent in non-smokers, with a higher incidence in adenocarcinoma patients with brain metastases.^63^ For patients with metastatic LUSC, consideration should also be given to testing for HER-2 mutations.

##### Other mutants

Dozens of other less common mutations have also been identified as oncogenic driver mutations of NSCLC, including BRCA2, SRC, DSP, RGL2, BTN3A2, and CCDC116, among others.^64^ In addition, loss-of-function mutations in tumor suppressor genes, such as TP53 and RB1, also frequently occur.^32^ Deeper studies are underway to further explore the therapeutic value of these pathogenic mutations.^65^

#### Non-mutational epigenetic reprogramming

Recent epigenetic advances in NSCLC have improved our understanding that epigenetic alterations, including DNA methylation, histone modification, and non-coding RNA regulation could drive the development, progression and invasion of tumor, as well as influence the response to drug therapy.

##### DNA methylation

DNA methylation comprises the transfer of methyl groups to the 5′ position of cytosine in a cytosine-guanine (CpG) dinucleotide, which is frequently found in high-density regions, termed CpG islands, typically located in gene promoters.^66^ Dysregulation of DNA methylation, including hypermethylation and hypomethylation, has profound effects on transcriptional regulation and imprinting. Recent studies have revealed that either global DNA hypomethylation or local hypermethylation, particularly in gene-specific promoters, appear to be closely related with tumor progression. In general, global genome hypomethylation is one of the hallmarks of cancer, which induces activation of proto-oncogene, loss of imprinting and genomic instability. High-resolution mapping of the DNA methylation status suggests that extensive DNA hypomethylation occurs specifically at repetitive sequences, including short and long interspersed nuclear elements and LTR elements, segmental duplications in lung cancers. Zhang et al. analyzed EGLN DNA methylation data from the tumor tissue samples of 1230 NSCLC patients, and the results showed that DNA methylation of EGLN2-HIF (hypoxia-inducible factor)1a affects the prognosis of NSCLC.^67^ SORT1 is downregulated in NSCLC and its epigenetic irregularities, especially DNA methylation level, is related to patients’ survival. Moreover, specific methylated genes in NSCLC, such as RASSF1A, SHOX2, APC and p16 (INK4a), have also been identified and could serve as diagnostic biomarkers.

##### Histone modifications

Histones, such as H2A, H2B, H3, H4, are proteins that form protein octamers around which genomic DNA is wrapped in eukaryotic cells. There are several types of post-translational modifications of histones, including acetylation, methylation, phosphorylation, and ubiquitination, as well as rare ADP-ribosylation, acylation and SUMOylation.^68^ Among these modifications, acetylation and methylation of histones, which are most extensively discussed, play an important role in lung cancer development by altering the structural properties of chromatin, thus regulating the transcription activation or repression of various oncogenes and tumor suppressors. Histone deacetylases (HDACs) catalyze the removal of acetyl groups from core histones, which are often overexpressed in cancers. Recent years, HDAC inhibitors have been developed to antagonize the reduced global histone acetylation observed in many tumor types, including NSCLC.^69^ The combination of HDAC3 inhibitor and trametinib has shown therapeutic benefits in genetically engineered mouse models of NSCLC. The YEATS domain of YEATS2 directly binds to histone H3K27 acetylation, and regulates a transcriptional program essential for NSCLC tumorigenesis. Additionally, the acetylation of SIRT6 disrupts its interaction with FOXA2, promoting ZEB2 expression and tumor progression in NSCLC.^70^ Abnormalities in histone methylation have also been reported to be closely related with tumorigenesis. EZH2 induces condensation of chromatin, thereby inhibiting the transcription of tumor suppressor genes.^71^ A bioinformatic analysis of methyltransferases and demethylases in NSCLC using TCGA and cBioportal databases showed that H3K27 methyltransferase EZH2 was significantly up-regulated while H3K27 demethylase KDM6B was significantly down-regulated in lung cancer. Furthermore, copy number variations and missense of other methyltransferases and demethylases were also detected in lung cancer patients, such as PRDM9, SETD1A, SMYD3, KDM5A and KDM5B.^72^ Taken together, the above research implicates that epigenetic alterations affect the key molecules involved in NSCLC and play an important role in tumorigenesis. The epigenetic-related signatures thus could serve as diagnostic and prognostic predictors, or as therapeutic targets for NSCLC.

##### Non-coding RNAs(ncRNAs)

Recent studies have transformed our perception of ncRNAs including microRNAs (miRNAs), long ncRNAs (lncRNAs), and circular RNAs (circRNAs), from seemingly redundant transcriptional products to functional RNAs that actively regulate various cellular processes, like epithelial to mesenchymal transition and cancer metastasis, by modulating gene expression and signal transduction.^73^ For instance, miR-196b-5p by downregulating TSPAN12 and GATA6,^74^ and miR-142-3p by activating the PI3K/Akt/mTOR pathway through HMGB1 inhibition,^75^ have been reported to drive tumor progression in NSCLC. In the meantime, increasing evidence suggests that circRNAs also may exert an impact on many types of transcripts with important functions in cellular homeostasis and thus serve as predictive biomarkers or therapeutic targets for NSCLC.^76^ However, the function of oncogenic lncRNAs is dichotomous, manifesting either as tumor-promoting, like LINC00673, LINC00173, and lncRNA-ATB, or as tumor-suppressing in NSCLC. Therefore, the flexible and dynamic changes in this epigenetic state can facilitate rapid or evolutionary responses to anticancer drugs, leading to the development of therapeutic resistance in patients.^73,77^ Related to methodological approaches, the future widespread use of single-cell sequencing tools, aided by AI, to determine these biomarkers not only simultaneously but also in a space and eventually in vivo in a temporal context, will constantly revolutionize this field.^78^

### Expansion of cancer cells

In terms of cancer cell expansion, the intratumor heterogeneity offers tumors the adaptability to survive, grow, metastasize and escape from immune attack. During the process of expansion and metastasis of lung cancer, cancer cells develop spatial and temporal diversity of genomic instability.^79^ Over the last decade, a series of TRACERx (TRAcking Cancer Evolution through therapy Rx) studies have extensively discussed the evolution and development of genomic intratumor heterogeneity in NSCLC.^80,81^ Hanjani et al. performed whole-exome sequencing on 327 tumor regions of 100 early-stage NSCLC tumors.^82^ Driver mutations, such as EGFR, MET, BRAF primarily occurred, and were almost always clonal. Branch mutations that occurred later were common in genes associated with chromatin modification and DNA damage repair, such as PIK3CA and NF1.^82^ Branch mutations occurred during tumor progression, are considered to drive cancer cell adaptations to external environment and are probably the cause of metastasis and therapeutic resistance.^31^ Recently, paired whole-exome and RNA sequencing data were investigated and analyzed by multiple machine-learning approaches to better understand the impact of transcriptomic features and their interplay with genomic diversity in NSCLC.^80^ Besides, the intratumor heterogeneity was also evaluated in multi-region NSCLC patient-derived xenograft models.^81^

Through unbiased single-cell RNA-sequencing (scRNA-seq) or other, high-resolution multi-omics technologies, studies mapping the cell type-specific transcriptomic landscapes within of NSCLC have revealed that tumors from different patients display extensive heterogeneity in cellular composition, chromosomal structure, developmental trajectories, intercellular signaling networks, and phenotypic dominance. Han et al. through scRNA-seq and high-resolution spatial transcriptomics (details provided in subsequent sections), studied 246,102 single epithelial cells from 16 early-stage LUAD and 47 matched normal lung samples. They found that KRT8-high expressing and driver KRAS-mutated cells are involved in the further development of lung cancer following tobacco carcinogen exposure.^83^ Similarly, scRNA-seq of NSCLC showed that poor prognosis is highly correlated with a subset of regulatory T cells expressing IL-1R2.^84^ However, gene variation markers for a specific cell type (or cell state) are almost never exclusive to those cells. Instead, they are also expressed in multiple other cell types/states, albeit at lower levels. Consequently, when applied individually, these 2D deconvolution methods may not be able to predict clear heterogeneity.^85^

### Metastasis

Metastasis, characterized as the growth of cancer cells in organs distant from the one in which they originated, is the ultimate manifestation of cancer, and is the major reason for treatment failure in NSCLC patients.^86^ Consistent with most solid tumors, the metastatic cascade of NSCLC collectively requires three phases that can overlap in time – dissemination, dormancy, and colonization – during which cancer cells undergo a succession of steps to invade tissues, survive in transit, and colonize organs, as well as importantly escape immunosurveillance.^86^ The multiple steps of metastasis are regulated by many factors, including the intrinsic signaling pathways that regulate EMT (epithelial-mesenchymal transition), angiogenesis and the interaction between the cancer cells and various components within TME. Notably, current research suggests that metastasis is not merely a simple point-to-point transfer, but rather a systemic mobilization process involving multiple tissues or organs. This process likely involves communication between tumor cells and bone marrow lymphocytes, as well as vascular endothelial cells.^87^ In the following, we summarized the studies on the mechanisms of NSCLC metastasis, and sought to provide guidance for the exploration of more efficient schemes for metastasis control.

#### Intrinsic pathways regulate cancer cell EMT formation

As an epithelial cancer, primary NSCLC invades the basement membrane and stroma at the first stage of metastasis. Through EMT, the epithelial-like cancer cells lose their polarity and convert into mesenchymal phenotype, thus acquiring metastatic abilities.^88^

During this process, the expression of epithelial markers such as E-cadherin and cytokeratin decreases, while the expression of mesenchymal markers, such as N-cadherin and vimentin increases. This leads to the loss of adhesion between epithelial-like tumor cells,^89^ and transformation into mesenchymal-like cells. Crucially, in the colonized organ, the aforementioned process may be reversed, manifesting as MET, which promotes the survival and proliferation of cancer cells.

Hypoxia, a status of oxygen deprivation of cancer cells and one of the hallmark features of solid tumors, plays a vital role in the regulation of metastasis. The best understood mechanism of hypoxic regulation of cancer cells is through the transcriptional activity of HIF-1/2. HIF-1α and HIF-2α are induced by hypoxia and coordinate the expression of numerous downstream genes that promote cancer cell invasion and angiogenesis, thereby shifting the cancer cells towards a metastatic phenotype.^90^ Specifically for the regulation of EMT, HIF-1α is reported to upregulate EMT-related transcription factors, such as Slug, TWIST and Snail in lung cancer,^90^ encoding them as repressors that block the expression of E-cadherin, which promote a flexible cytoskeleton and the characteristics of a mesenchymal phenotype (Fig. 3).

Pathogenic mutations in NSCLC patients also affect the metastasis of the tumor cells. Numerous studies have implied that EMT transmission is closely related to EGFR TKI resistance in the EGFR-mutant cell lines and patient tumors of NSCLC. Downstream signaling pathways, such as PI3K/AKT and RAS/MAPK, can be activated by EGFR and subsequently promote tumor progression and metastasis.^91^ EGFR has also been reported as a hypoxia-independent driver of HIF expression. Alteration of HIF by EGFR signaling could further promote the EMT transformation.^92^ The overexpression of EML4-ALK in NSCLC cell line induces the EMT phenotype, and upregulates the expression of EMT-related transcription factors, which could be reversed by an inhibition of ERK1/2. CRKL has also been identified as a key downstream effector of ALK-induced EMT. Knockdown of CRKL decreases cell migration ability through mediating the downstream ALK signaling pathways, such as RAS/Rac1 (Fig. 3).

Recently, non-coding RNAs such as miRNAs have also been identified as potent modulators and biomarkers of EMT. By investigating the expression of 207 miRNAs in various cancer cell lines, Park SM et al. identified the miR-200 family as representative markers for cells with epithelial phenotype, which could directly target the mRNA of ZEB1 and ZEB2 (E-cadherin transcriptional repressors), leading to the up-regulation of E-cadherin, thus inhibiting the EMT process.^93^ In EGFR-mutated cancers, members in miR-200 family have also been reported to be downregulated, thus enhance the drug resistance and EMT characteristics of NSCLC. Z-M Shi et al. reported that miR-218 was significantly downregulated in lung cancer tissues, which contributed to EMT process by mediating Slug/ZEB2 signaling. Numerous miRNAs, such as miR-15b, miR-200b/c, miR-140, miR-224, miR-34c, etc. ^94^ have also been identified as EMT-related signatures in NSCLC cell lines.

#### Pathways priming the tumor microenvironment

TEM is composed of malignant cells, and various stromal cells, such as cancer associated fibroblast (CAFs), immune cells and endothelial cells, along with extracellular matrix (ECM), all of which support tumor survival and progression. The coordination between cancer cells and TME forms the foundation of the metastatic process.^86^ A deeper understanding of the complex interplay between the tumor cells and their microenvironments during the progression of NSCLC will be helpful in developing effective treatments against tumor metastasis.

##### CAFs

CAFs, the predominant cells within NSCLC stromal component, are closely associated with poor outcome in NSCLC patients.^95^ TGF-β and exosomes carried abundant non-coding RNAs, such as miRNAs and lncRNAs are secreted from NSCLC cancer cells, and modulate the functions of CAFs (Fig. 3). Acting as the output ports, the activated CAFs could promote tumorigenesis and metastasis of cancer cells via the secretion of biologically active substances that stimulate the cancer cell invasion and angiogenesis, recruit tumor associated macrophages, suppress T-cell antitumor immunity and remodel the ECM. CAF-derived cytokines, such as IL-6, IL-10, could activate the JAK/STAT pathways in cancer cells. Besides, MAPK, PI3K/mTOR and Wnt/β-catenin signaling are also activated in cancer cells in response to the secretion of growth factors and cytokines, both of which promote cancer cell proliferation or EMT transformation.^96^

CAFs also participate in the synthesis of structural proteins of the ECM, such as type I and type IV collagen, and secrete proteases, such as MMP2 and MMP9, to degrade and reshape the ECM,^97^ resultantly promoting cancer cell survival, invasion and metastasis. One possible mechanism is that altered mechanical stimuli and forces within the TME activate the most prevalent mechanosensitive molecular signaling pathways, including Yes-associated protein (YAP), Wnt-β-catenin, and PIEZO1, as well as other oncogenes and metabolic pathway genes, such as ENO2, KCNG1, and PFKFB3.^98^

##### ECM

ECM is the non-cellular component that provides the architecture around cancer cells. The progression and metastasis of malignant tumors are often accompanied by the alterations of ECM content and structure. ECM degradation is essential for the early steps of the metastatic cascade. Genetic polymorphisms of the genes related to ECM regulation (MMP 2,3, 9) have been reported to be correlated with the risk and survival of lung cancer. Except for the regulation mediated by CAFs described above, other cell components within TEM also involved in the remodeling of ECM during metastasis. Cancer cells and TAMs contribute to the degradation of ECM via secretion of cytokines and protease expression alterations.^99^ The quantitative proteomics analysis of ECM protein composition in primary lung tumors and metastases revealed specific signatures of tumor ECM associated with metastatic process, such as fibronectin and tenascin-C are significantly accumulated.^100^ Moreover, TNC, S100A10 and S100A11 showed prominent potential in the prediction of patient survival, which may serve as diagnostic biomarkers and therapeutic targets in the future.

##### Immune cells

In order to grow progressively and develop metastases in distant sites, cancer cells must develop immune escape from the immune cells in TME. As the major killers fighting against cancer cells, the presence of cytotoxic CD8^+^ T lymphocytes is correlated with better NSCLC patient outcomes.^101^ The loss or downregulation of antigen-presenting machinery, such as MHC-I, along with the secretion and expression of immunosuppressive factors, like TGF-β, IL-6, IL-10, IDO and PD-L1, are often described as mechanisms through which metastatic cancer cells avoid T cell recognition and killing or compromise T cell activation and proliferation.^102^

TAMs, dendritic cells (DCs), and myeloid-derived suppressor cells (MDSCs) are classical innate immune cells in TME. It is widely known that TAMs are highly dynamic, and their polarization induces switching between antitumorigenic M1 and pro-tumorigenic M2. M1 TAMs could directly kill tumor cells by secreting cytokines such as TNF-α, IL-6, and IFN (interferon)-γ (Fig. 3), or by inducing the production of ROS and NO. Highly-expressed IL-17 and PGE2 in cancer tissues are involved in the recruitment and differentiation of TAMs, thus creating an M2 Macrophage-dominant and immunosuppressive TME in NSCLC (Fig. 3). M2 TAMs secrete a series of growth factors and chemokines that facilitate the metastasis of cancer cells in multiple different ways. They can express high levels of Cathepsin K, COX-2, MMP-9, PDGF-B, VEGFA, and HGF, which contribute to the ECM remodeling, promote angiogenesis and affect EMT transformation of cancer cells. In general, the extensive cross-talk among TAMs, tumor cells and other components within TME, such as monocytes, neutrophils, NK and other innate lymphoid cells, provide a favorable tissue environment (or pre-metastatic niche) that supports the persistence of disseminated tumor cells within a foreign tissue, and ultimately promotes invasion and metastasis of NSCLC.^87^

#### Pathways regulate angiogenesis

It is now widely accepted that angiogenesis is a critical process during tumor expansion and metastasis, which provides essential nutrients and oxygen to cancer cells. After decades of research, an amount of signal molecules promoting tumor angiogenesis has been discovered. Among all the identified regulators, the VEGF family and their receptors (VEGFR1/2/3) seem to be the most critical ones^103^ (Fig. 3). Either the cancer cells or the other cell components in TME could secrete VEGF to promote endothelial cell migration and blood vessel formation. The involvement of other important molecules has also been revealed to work together with VEGF/VEGFR signaling during the angiogenesis process, including FGFRs and their ligands, particularly FGF1 and FGF2. FGF/FGFR signaling enhances the proliferation and migration of endothelial cells. Besides, the PDGFR-related pathways and the Ang-Tie-2 system are also tightly associated with tumor vascularization in NSCLC.^104^

In summary, tumors influence multiple organs and systems either directly through tumor-derived mediators (including associated growth factors, cytokines, and chemokines, which are partly released by extracellular vesicles, as well as through platelet-CTC interactions), or indirectly via disruptions of circadian rhythms and gut dysbiosis, which perturb specific organ functions, metabolic systems, and immunosuppressive myelopoiesis. This widespread physiological disruption can manifest as pro-metastatic conditioning in the early stages and may eventually lead to the proliferation or dormancy of disseminated cancer cells.^87^ However, the control of metastatic tropism is multifactorial and is not necessarily determined solely by the intrinsic properties of the cancer cells, meanwhile diet- and lifestyle-induced conditioning (such as nicotine-induced inflammation) and aging-induced conditioning can also influence metastatic progression. Moreover, whether organ-specific metastases are stochastic or deterministic phenomena may be related to both the characteristics of the cancer cells themselves, such as altering aspects of their metabolism,^105^ and the host organ’s suitability,^106^ although the exact mechanisms remain unclear.^102^

### Spatial features within TME involving NSCLC

The TME is a complex and dynamic ecosystem composed of diverse cell types and extracellular components. Within this environment, immune cells, such as T cells, typically cluster around endothelial cells, while macrophages display varied distribution patterns, ranging from uniform dispersion to aggregation. Tumor-associated TLS (tertiary lymphoid structures) or vasculature development has been shown to influence the efficacy of cancer immunotherapies.^107^ However, traditional 2D IMC (immunohistochemistry) fails to capture cells positioned above or below the slice plane, obscuring the intricate cellular relationships and regional heterogeneity that define the tumor landscape. Thus, leveraging multimodal spatial omics technologies to visualize the heterogeneity and spatial architecture of the TME using generative AI, like machine learning or DL^108^ is critical for uncovering the underlying mechanisms driving tumor progression and therapeutic responses,^109^ especially for immunotherapy closely related to the spatial positioning of cells.^110^

In the spatial distribution analysis of molecular structures within individual cells, spatial genomics (SG) and spatial chromatin organization (SCO) analyses, such as sequential DNA FISH, RNA seqFISH,^111^ and CHi-C, capture Hi-C, chromatin immunoprecipitation (ChIP), and Cut&Tag (cleavage under targets and tagmentation), enable the mapping of sequencing data to spatial positions within cells and subcellular compartments. This not only facilitates the identification of specific genomic sequences, including copy number alterations (CNAs) and somatic mutations,^112^ but also the spatial proximity of discontinuous DNA regions, such as the configuration of topological domains (TADs) and DNA-chromatin protein interactions.^113^ Given their pivotal roles in tumor initiation and progression,^114^ these spatial features have been proposed as prognostic markers and predictive biomarkers. New broad-spectrum drugs targeting epigenetic enzymes and chromatin remodelers are entering clinical trials, however, reliable biomarkers predicting responses to these drugs remain limited. More comprehensive analyses of chromatin conformation can facilitate identify tumors that are sensitive to these novel therapeutic strategies.^115^

The characterization of cellular spatial structures within the TME sticks to a multi-step process, beginning with extensive serial sectioning of valuable tissue samples (formalin-fixed paraffin-embedded or frozen),^116^ followed by various staining, imaging, sequencing, mass spectrometry, and radiological techniques. Finally, the spatial arrangements, proximities, and relationships among various cells of interest are indexed, grouped^117^ and interpreted quantitatively.^118^

For spatial transcriptomic characterization, one approach utilizes sequencing-based spatial indexing methods, such as non-invasive DNA barcoding,^119^ ZipSeq by printed spots, XYZeq by microwells, and Stereo-Seq by DNA nanoballs, alongside Slide-seq, Drop-seq, and HDST (high-definition spatial transcriptomics) by beads.^116,120^ Another involves imaging-based methods, including FISSEQ (fluorescence in situ sequencing), PLISH (proximity-ligation in situ hybridization), BOLORAMIS (barcoded oligonucleotides ligated on RNA amplified for multiplexed and parallel in situ analyses), BaristaSeq (barcode in situ targeted sequencing), SCRINSHOT (single-cell-resolution in situ hybridization on tissues), ExSeq (expansion sequencing), and STARmap (spatially resolved transcript amplicon readout mapping).^116^ For spatial proteomic characterization, methods include immunofluorescence and cyclic microscopy, such as mIHC (multiplexed immunohistochemistry), MxIF (multiplexed fluorescence microscopy), IBEX (iterative bleaching extends multiplexity), MELC (multi-epitope ligand cartography), t-CyCIF (tissue-based cyclic immunofluorescence), or MICS (MACSima imaging cyclic staining).^116^ Additionally, mass spectrometry techniques, such as time-of-flight (TOF) mass spectrometry imaging (MSI), MIBI (multi-ion beam imaging), high-energy mass spectrometry imaging cytochemistry,^121^ Orbitrap (like OrbiSIMS), and NanoSIMS mass spectrometric analysis, have been employed. However, there is an inverse relationship between the number and speed of captured markers and the resolution/biological scale of capture. As the biological scale shifts towards extreme granularity, the ability of multiplexing decreases, and both acquisition time and costs tend to increase.

While maintaining the integrity of entire organ systems, machine learning techniques like CODA (a method for 3D visualization of tissue structures in large tissue volumes >1 cm^3^),^122^ combined with cell morphology-based real-time cell sorting (COSMOS) or scGPT,^123^ EcoTyper,^124^ and Live-seq,^125^ enable the delineation of interactions among specific tissue components, such as various cell types as seen in TLS, microbes and metabolites, as well as subcellular organization of proteins,^126^ and also facilitate tracking and localization of lineage relationships among immune cells to infer developmental trajectories.^127^ Deep learning applied to spatial proteomics IMC datasets from early-stage NSCLC patients can predict individuals at high risk of recurrence with over 95% accuracy or acquired resistance to anti-PD1 therapy,^128^ offering potential for developing more targeted therapeutic strategies. Combining DL with other spatialomics datasets (e.g., radiographic images) enhances cancer detection accuracy by identifying subtle characteristics often overlooked by humans, leading to more efficient and effective cancer treatments.

Notably, each technology or specific platform has its unique advantages, as well as inherent limitations, characterized by their labeling ranges, precautions, proprietary reagents, optimization efficiency, sample running efficiency, operating costs, and analytical complexity. Despite considerable progress, current SO, SG, and SCO analytical tools have limited direct clinical applicability, while ST and SP technologies are more advanced in terms of clinical translation.^129^ One major obstacle in spatial omics advancement is the limited accessibility of these analyses to routine research laboratories, necessitating specialized bioinformatics expertise. Moreover, many of these techniques require live samples, posing significant challenges in generating comprehensive patient datasets efficiently. Furthermore, there is a need to integrate the spatial-omics characteristics of both tumor and non-tumor cells with the TME, host-wide microbiome, neuronal, and hormonal signals.

In conclusion, at present, we are in an exciting period of understanding the mechanisms of complex biological systems in NSCLC, through the integration of multiple spatial or omics methods. This is because of the rapid evolution and decreasing costs of biological analytics and the explosive growth in AI,^130^ coupled with collaborative efforts between biologists and computer scientists,^131^ aiming to push forward a comprehensive understanding of cancer biology, drive drug development, and improve treatment precision for NSCLC.^132^

## Biomarkers in NSCLC

The cornerstone of precision medicine lies in leveraging molecular biomarkers for diagnostic and therapeutic decision-making to optimize efficacy while minimizing unnecessary toxicity. Tumor markers, as biochemical indicators, have evolved from their historical role in cancer diagnosis, such as the detection of Benjamin Jones proteins in urine indicating myeloma in 1846, and cancer screening, to present-day applications including guiding drug selection, predicting treatment outcomes, and assessing patient prognosis^133^ etc., and even have profoundly enhanced our understanding of mechanisms underlying therapeutic resistance.^134^ The scope of tumor markers has expanded from detectable proteins or nucleic acids (DNA or RNA)^135^ in plasma or other bodily fluids to encompass a range of genetic mutations, cellular function states, and even spatial distributions of cells and structures.

### Detection of biomarkers

Next generation sequencing (NGS), is currently the most widely used platform for detecting genomic aberrations, and is capable of identifying a variety of mutations, gene fusions, and copy number variations, with some minor content discrepancies existing among different commercial or institutional laboratory testing platforms. Gene rearrangements or amplifications can be detected through methods such as fluorescence in situ hybridization (FISH) or others.^136^ Based on the pathogenicity, of gene variants are typically categorized as having strong clinical significance, possibly clinical significance, uncertain clinical significance, benign, or very likely benign. Laboratories usually do not report non-pathogenic variants.

In scenarios where tissue sampling is inaccessible, circulating tumor DNA (ctDNA) from free DNA, or exosomes and CTCs^137^ in blood or pleural/abdominal fluids can be useful as a minimally invasive diagnostic approach, particularly for late-stage patients, although the standards or guidelines for somatic variant/mutation detection in blood-derived ctDNA have not been established. Moreover, ctDNA-based NGS can detect a broader range of cell clones, enhancing the chance of capturing rare mutations, yet these ctDNA signatures might not solely pertain to NSCLC, but also indicate clonal hematopoiesis of indeterminate potential (CHIP), commonly observed in older patients, or following chemotherapy or radiotherapy.^138^ Conversely, the false negative rate in ctDNA testing is strikingly high at 30%, supported by instances where a ctDNA TF value under 1% results in a negative test outcome, often subsequently revealing the presence of driver mutations through tissue testing, highlighting the necessity for careful consideration when interpreting ctDNA results.^139^ However, some studies have suggested that ctDNA methods analyzing low-frequency single nucleotide variants (SNVs) down to 0.1% can identify cancers with low disease burden before metastasis, indicating that ctDNA may serve as an effective screening tool.^140^ Similarly, in solid tumor, ctDNA is being employed to accurately gauge minimal residual disease (MRD) and monitor a variety of therapeutic responses,^141^ while the introduction of the AI-guided detection platform (MRD-EDGE), has significantly broadened its applicability within the field of solid tumors.^142^ Compared to ctDNA, proteomics and transcriptomics or epitranscriptomics^78^ based on liquid biopsy are still in their early stages of application for NSCLC, where monitoring dynamic changes in PD-L1 levels within CTCs can predict treatment response,^143^ but they are likely to assume a more prominent role in the future.

### Biomarkers in the diagnosis of NSCLC

Historically, in diagnosing NSCLC, it was common to observe a pronounced increase in CEA levels and occasional slight elevations in NSE, with a specificity not exceeding 40%,^1^ particularly in early-stage patients. Currently, NGS leverages DNA methylation patterns to not only determine whether an unknown primary site (CUP) cancer is NSCLC^144^ and inform on treatment decisions,^144,145^ but to also assess if the multiple lung cancers or lesions in the lungs originate from the same clone,^146^ particularly when they share the same histological type without lymph node involvement or distant metastasis. In the future, with the advancement of NGS for DNA or non-coding RNAs,^147^ mass spectrometry analysis and AI, the efficiency and specificity of diagnosing NSCLC will be our primary focus areas, such as using ctDNA for real-time monitoring of MDR.^148^

### Biomarkers in the treatment of NSCLC

#### Biomarkers for targeted therapy

For all appropriate NSCLC patients with non-squamous or unclassified histological types and occasionally including LUSC, specific molecular and immunological biomarkers should be tested, although the size of the panel may vary across different stages of the disease.^149^

**Fig. 4:**
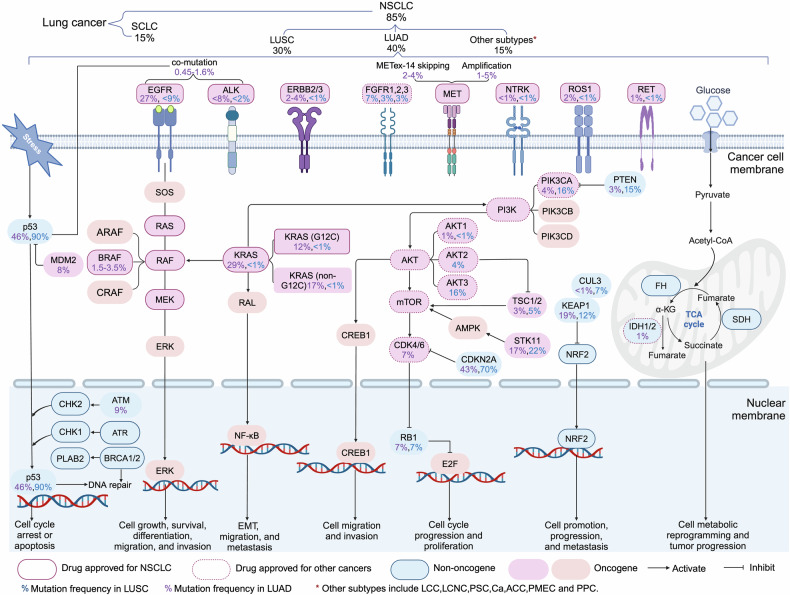
Alternations in oncogenic and anti-oncogenic pathways involving drug targets or biomarkers in NSCLC. In NSCLC, numerous activated or upregulated intracellular oncogenic and non-oncogenic protein kinase signaling pathways have been identified. However, we primarily focus on targets that are or will become druggable, highlighting their mutation frequencies. Although mutation frequencies reported in various literatures or databases may vary, the overall differences are not significant. The illustration may not fully capture the diversity and functional complexity of these signaling pathways and their interactions in vivo. Additionally, due to space limitations, extracellular or microenvironment features such as the dependence of cancer cells on VEGF/VEGFR signaling, other metabolic pathways (beyond glucose), and hypoxia are not depicted. LCC large cell carcinoma, LCNC large cell neuroendocrine carcinoma, PSC pulmonary sarcomatoid carcinoma, ASC adenosquamous carcinoma, ACC adenoid cystic carcinoma, PMEC pulmonary mucoepidermoid carcinoma, PPC pulmonary pleomorphic carcinoma

The biomarkers for targeted therapies in NSCLC predominantly serve a predictive function, shedding light on therapeutic outcomes, while a smaller subset acts as prognostic indicators, gauging overall survival prospects (such as KRAS mutations), though the delineation between the two is not absolute. Predictive molecular biomarkers are our primary focus, encompassing ALK, RET, ROS1, and NTRK1-3 rearrangements, along with BRAF^V600E^, EGFR, KRAS and ERBB2 mutations, MET exon 14 skipping mutations, and genetic amplifications (Fig. 4), since they are all clinically actionable targets (which already are discussed in Section 3 and will be discussed in detail in later sections). Nevertheless, for selected patients, it is strongly recommended to undergo comprehensive molecular sequencing to uncover rare driver mutations, which could qualify them for participation in specific drug clinical trials.

#### Biomarkers for immunotherapy

A defining yet challenging aspect of immunotherapy, including ICIs, is its selective efficacy, hence, our overarching aim is to identify patient populations that respond to treatment.^150^ Current immunotherapy biomarkers include those related to tumor cells, such as tissues PD-L1 and soluble PD-L1 expression, TMB, dMMR/MSI-H, the quality and quantity of neoantigens, antigen presentation pathways, or specific genes (like TROP2 expression^151^) and chromosomal arm alterations,^152^ which can be evaluated through tissue biopsies, circulating cell-free DNA,^141,153^ or CTCs. Another category is biomarkers related to non-tumor cells, for example, tumor-infiltrating lymphocytes (TILs), circulating antigen-specific T cells, TLS,^154^ the microbiome, circulating L-arginine^155^ or L-alanine, PD-L1 expression on non-cancerous cells or platelets, and host factors such as the diversity of HLA gene types, gender, and smoking history.^133^

Nonetheless, currently, the only biomarkers that have been incorporated into clinical practice are the expression of PD-L1 in tissue and dMMR/MSI-H, despite their imperfections, primarily because of their reduced specificity.^156^ This heterogeneity in PD-L1 expression over time and space, which is influenced by interferons and diverse immune signaling pathways during treatment, plays a key role here. Furthermore, the use of different diagnostic platforms and evaluation methodologies may have set varying positive thresholds, contributing to the discrepancies in the identification of PD-L1 positivity.^157^ PD-L1 expression derived from CTCs^153^ and DCs^158^ exhibit high predictive potential, albeit constrained by detection techniques (with a half-life of about 25–30 min for a single CTC^131^). Soluble PD-L1, as an independent biomarker, might offer the necessary resolution and rich data for relevant analyses, yet its positive threshold requires further determination.^159^ Similarly, dMMR/MSI-H, owing to pronounced inconsistency and lower prevalence in lung cancer, has limited predictive value in NSCLC. Higher TMB can facilitate the production of putative neoantigens, correlating with better outcomes for ICIs, however there are also mixed predictive results. Post-transcriptional events, such as alternative splicing, intron retention, non-classical translation initiation, and codon misreading, along with long non-coding RNAs and pseudogenes, can generate unconventional antigens that stimulate T-cell responses (also referred to as alternative, occult, or dark matter antigens).^160^ Bacterial or viral remnants may also possess the capability to trigger antitumor T-cell responses through molecular mimicry or cross-reactivity with other tumor-associated antigens. Another reason is the inconsistency in defining high or low TMB standards and the inherent heterogeneity of the tumor itself,^161^ along with the variability in the materials used for detection, including circulating tumor DNA-based TMB (bTMB) versus tissue-based TMB (tTMB).^162^ CD8^+^TILs, a widely researched biomarker, poses difficulties in distinguishing responsive patients through a singular high/low parameter, as these cells are categorized into early-exhausted T cells and late-exhausted T cells, with only the former having a relevant association with the effectiveness of immunotherapies.^163^ Furthermore, biomarkers such as mutations in DNA repair genes or oncogene genes, and the dNLR (derived neutrophil to lymphocyte ratio), also provide significant insights into the probability of a patient’s response to immunotherapy.

Currently, it is beyond doubt that a singular parameter cannot accurately predict the therapeutic efficacy for a specific drug category, hence, an ideal biomarker must possess multifaceted and diversified attributes to enhance its diagnostic discernment capability.^164^ Indeed, several biomarker scoring systems have been developed that integrate various factors including TMB, tissue PD-L1 expression, circulating CD8^+^ T cell scores, ctDNA levels, HLA variants, neoantigen landscapes, and aneuploidy levels, and partially leveraging AI models such as Immunoscore,^165^ DIREct-On (estimating durable immune therapy response based on immunophenotype and ctDNA),^166^ multi-gene expression scores, and the CODEFACS (COnfident DEconvolution For All Cell Subsets) system.^167^

#### Biomarkers for radiotherapy

Radiotherapy is a pivotal treatment modality for NSCLC, with treatment decisions primarily hinged on patient factors (such as age, health status, or comorbidities) and tumor biological characteristics (including location, size, staging, and subtype).^168^ Unfortunately, as of now, there is no broadly accepted tumor or radiological biomarker,^169^ despite the roles played by DNA damage, hypoxia, proliferation, stem cell phenotypes, and immune regulation in modulating radiation sensitivity.^170^

##### Biomarkers related to inherent tumor cell characteristics

Radiotherapy universally triggers atomic excitation and ionization within targeted tissues, resulting in vital DNA double-strand breaks (DSBs). The primary mechanisms for repairing these DSBs involve non-homologous end joining (NHEJ) or high-fidelity homologous recombination repair.^171^ Consequently, mutations in DNA damage repair pathways, including TP53, KEAP1, NFE2L2, KMT2C, and KMT2D, are significantly correlated with notable radioresistance in NSCLC. Similarly, the high expression of proteins involved in DNA damage repair within tumors, such as ERCC1/2, MRN, and MRE11, theoretically enhances radiation resistance, yet contradictory results have been observed.^169^

On the other hand, deleterious mutations in BRCA1/2 or ATM, or both, as well as in RAD51 or PTEN, have been confirmed to be associated with increased radiosensitivity. Driver mutations, such as KRAS mutations and ALK rearrangements, typically are correlated with higher sensitivity to radiotherapy, although the research findings have been inconsistent. An increasing number of RNA-based classifications, such as the Radiosensitivity Index (RSI) based on a 10-gene signature, also can predict a tumor’s radiosensitivity, although formal validation of these methods in NSCLC has not been conducted.^172^ Radioresistance may also be associated with highly hypoxic tumors characterized by high levels of HIFs, genomic instability involving interactions between the unfolded protein response (UPR) and mTOR, and ferroptosis-associated genes,^173^ as well as upregulated lactate metabolism. Indeed, in NSCLC, the activation of HIF-1α and EGFR, which has been shown to induce a CSC (cancer stem cell) phenotype, and the activation of the RAS-RAF-MEK-ERK or lipoyltranferase 1^174^ signaling pathway, along with the presence of markers for tumor regeneration such as high Ki-67 expression and the overexpression and enhanced activity of indoleamine 2,3-dioxygenase (IDO), are all associated with radioresistance.^175^

##### Biomarkers related to non-tumor cell characteristics

Multiple immunogenetic signatures associated with radiotherapy response have been developed, including TMB, PD-L1 status, and absolute lymphocyte counts prior to treatment, and high intratumoral density of CD8^+^ T cells post-radiotherapy, which may be used to predict the benefit of radiotherapy.^176^ Other potential biomarkers include gender, with evidence suggesting higher radio-sensitivity in female patients, as well as HPV infection^177^ or the presence of certain gut bacteria that can enhance the post-radiotherapy immune response. Conversely, the upregulation of HIF-1α increases the production of CXCL12, VEGF and FOXP3, activates T_reg_ cells, myeloid-derived suppressor cells, and tumor-associated fibroblasts,^178^ as well as promotes the polarization of macrophages and monocytes from an antitumor phenotype (M1) to a pro-tumor phenotype (M2), leading to radioresistance.

#### Biomarkers for antibody-drug conjugates (ADCs)

ADCs are special drugs that combine targeted drug properties with the effects of chemotherapy. Currently, over 13 ADCs are being applied in cancer treatment.^25,179^ Note that, clinical ADCs are not always target-driven, and there is still controversy regarding whether the target antigen expression decides the main activity of ADCs. Indeed, T-DM1 (trastuzumab emtansine) and T-DXd were initially approved only for HER-2 positive breast cancer patients,^180^ however, in the DESTINY-Lung01 trial, only NSCLC patients with HER-2 mutations, rather than those with HER-2 amplification or overexpression, showed significant benefit, leading to the accelerated approval by FDA.^181^

The unexpected toxic effects of ADCs, including on-target/off-tumor and off-target/off-tumor toxicity, are influenced by both the differential expression of the ADC targets in tumor tissues compared to healthy tissues, and the characteristics of the payloads, highlighting the need for careful consideration.^182^ A special concern regarding the administration of T-DXd is drug-induced interstitial lung disease,^183^ which might be associated with factors such as dosages of T-DXd exceeding 6.4 mg/kg weekly, Japanese ancestry, pre-existing pulmonary complications, diminished renal function, a disease diagnosis of more than 4 years post-diagnosis, and a baseline oxygen saturation below 95%, however, the exact mechanisms are not yet fully elucidated.

In summary, looking towards the future, advancements in various sequencing technologies, such as spatial transcriptomics and proteomic barcode techniques,^184^ coupled with improvements in bio-synthetically derived metabolic analysis, combined with clinical radiology data (like metabolic tumor volume assessed by PET-CT),^185^ pathological image datasets, and real-world data, when integrated with the application of AI for multimodal dynamic analysis,^186^ like clinical histopathology imaging evaluation foundation (CHIEF) model,^187^ may represent the optimal solution for significantly enhancing the precision in predicting NSCLC treatment efficacy.

## Therapies for resectable or non-advanced stage NSCLC

Within NSCLC, 20% of cases are categorized at stages I or II, 30% at stage III (indicative of localized advanced disease), and 50% at stage IV.^188^ Notably, locally advanced NSCLC, characterized by large tumor volume, invasion of adjacent structures, or regional lymph node metastasis, represents a highly heterogeneous disease, necessitating multidisciplinary decision-making in treatment selection to ultimately enhance survival rate.^189^

### Diagnosis and staging

For all patients, initial thoraco-abdominal CT scans should be performed, along with contrasted brain magnetic resonance imaging (MRI) or positron emission tomography (PET)-CT, even invasive biopsy methods, to obtain information on occult or distant metastases.^190^ At the initial diagnosis stage, assessing PD-L1 levels,^191^ EGFR, and ALK mutations to guide neoadjuvant or adjuvant therapies^149^ is always recommended. However, current consensus guidelines do not prioritize the performing of comprehensive genomic sequencing analysis.

The definition of risks for thoracic surgery is continuously evolving, yet preoperative assessments primarily focus on cardiac function and lung function testing, as they represent the highest risk for adverse post-surgery outcomes. In a recent publication by the American association for thoracic surgery, the three most critical parameters are highlighted for patients with reduced lung function, including testing common lung function (including measurements such as forced vital capacity and systemic assessments of carbon monoxide diffusion capacity, calculating the anticipated postoperative lung function, and conducting exercise tests.^192^ Notably, over half of the candidates for NSCLC resection are found to be frail or pre-frail. Fortunately, the emergence of neoadjuvant immunotherapies and targeted therapies may offer opportunities for patients to undergo rehabilitation prior to surgery.

### Neoadjuvant or perioperative therapy

In clinical practice, the conventional protocol for patients within stages I-II typically emphasizes administering surgery first, succeeded by adjuvant chemotherapy, as opposed to the reverse order. However, the optimal therapeutic strategies for stage III diseases (most equivalent to locally advanced) have long been a subject of debate over the past three decades, particularly concerning how to integrate surgical or radiation therapy with systemic treatments.^193^ Based on the following facts, firstly, the efficacy for stage III patients is unsatisfactory. Among locally advanced NSCLC patients who undergo complete resection, more than 50% still experience recurrence. While recent data from high-throughput centers suggests that patients with III A-N2 disease treated with surgery after induction chemotherapy achieve a slight increase in their 5-year survival rate, reaching approximately 40%,^194^ neoadjuvant chemotherapy only can boost the 5-year OS by about 4–5%. Secondly, the efficacy of local treatments such as surgery and radiotherapy has reached a plateau, failing to achieve significant improvement, despite with high-level precision – associated lower side effects. Finally, various immunotherapy drugs have achieved notable success in advanced patients. Moreover, recent preclinical and clinical evidence suggests that stronger immune responses may occur when the primary tumors and their lymph nodes coexist – a hypothesis that forms the basis for neoadjuvant immunotherapy strategies.^195^

**Fig. 5:**
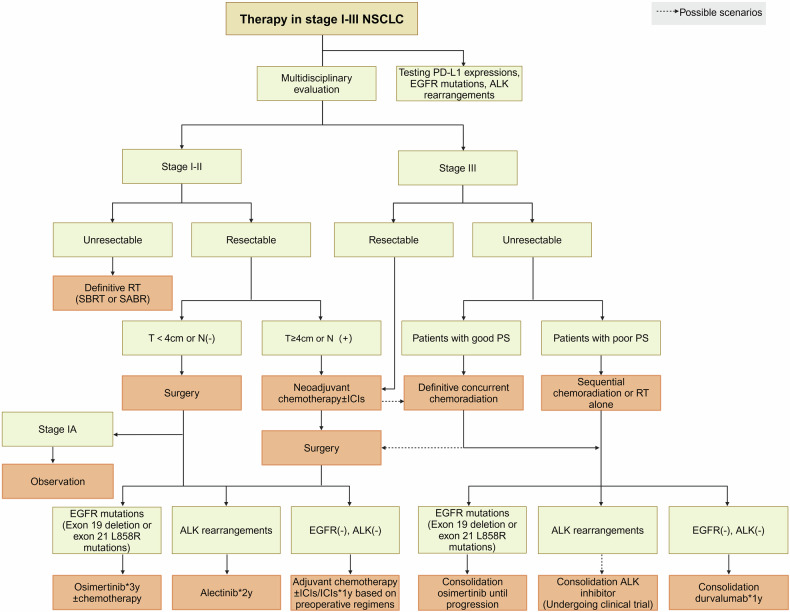
An algorithm of evidence-based management for stage I-III NSCLC. This flowchart comprehensively illustrates the treatment options for early-stage lung cancer, maintaining flexibility through the use of dashed lines to indicate alternative choices. However, this illustration focuses primarily on multi-level drug options and does not detail more technical treatment options such as radiotherapy and surgery, which may be more crucial for certain patients. For neoadjuvant regimens, particularly the combination of chemotherapy with ICIs, the recommendation level remains consistent across different options

Therefore, researches on the (neo)adjuvant treatment of immunotherapy or targeted therapy used alone or in combination with chemotherapy in non-advanced patients, particularly stage III patients, are tremendously proliferating (Fig. 5). With regard to the application of neo-adjuvant ICIs alone in early-stage diseases, the results of multiple clinical trials, such as phase II CHECKMATE-159, LCMC3, PRINCEPS, IONESCO, and ChiCTR-OIC-17013726 (Table 1), in which surgeries were performed after single-agent therapy, demonstrate significant clinical benefit, as evidenced by major pathologic remission (MPR) rates of 11-45% and pathologic complete response rates of 4-29%. Furthermore, the neoadjuvant trials involving ICIs combined with chemotherapy such as NADIM,^196^ NADIM II,^197^ NEOSTAR, NeoCOAST, CHECKMATE-816,^198^ SAKK 16/14, ChiCTR1900023758,^199,200^ KEYNOTE-671,^201^ NEOTORCH,^202^ CHECKMATE-77T,^203^ NEOpredict-Lung, EAST ENERGY and AEGEAN,^204^ among others (Table 1), have demonstrated an enhancement in clinical efficacy with treatment, which is also supported by the real-world results from a retrospective analysis.^205^ Consequently, for patients with resectable stages III (occasionally stages II) without EGFR or ALK mutations, a standard treatment approach at this juncture involves neoadjuvant chemoimmunotherapy followed by surgery, and then by either observation or the subsequent adjuvant ICIs.^206^ Another viable strategic option is neoadjuvant chemotherapy followed by surgery, subsequently complemented with adjuvant ICIs (Table 1). In addition, neoadjuvant treatment concurrently combined with chemoradiotherapy (CCRT) for an entire inoperable stage III patient cohort has failed to show improved survival times in clinical trials during pre-immunotherapy era, including INT0139, SAKK16/00, and WJTOG9903. Therefore, whether to incorporate immunotherapy into induction CCRT may potentially innovate the current treatment landscape, and poses an area of interesting and active clinical investigation.

**Table 1:** Drug therapies for non-advanced stage NSCLC

Therapeutic settings	Trial names	Assessed regimes	Trial interventions	Patient stages	PD-L1 status/Gene mutation status	Primary endpoints	Regulatory status
Perioperative therapy	LCMC3 Phase II (NCT02927301)^802,803^	Atezolizumab	Atezolizumab (neoadjuvant, and then optionally adjuvant)	IB-IIIB(T3N2, not T4 or N3)	Regardless of PD-L1 levels	MPR: 20.0%	
	KEYNOTE-671 Phase III (NCT03425643)^804^	Pembrolizumab + Chemotherapy	Pembrolizumab + Chemotherapy vs. Placebo + Chemotherapy (neoadjuvant) and Pembrolizumab vs. Placebo (adjuvant)	II-IIIB (N2)	Regardless of PD-L1 levels	EFS: 47.2 months vs. 18.3 months 36-month OS: 71.0% vs. 64.0%	FDA-approved/2023
	AEGEAN Phase III (NCT03800134)^204^	Durvalumab + Chemotherapy	Durvalumab + Chemotherapy vs. Placebo + Chemotherapy (neoadjuvant) and Durvalumab vs. Placebo(adjuvant)	IIA-IIIB (≥4 cm)	Regardless of PD-L1 levels	month EFS: 73.4% vs. 64.5% pCR: 17.2% vs. 4.3%	FDA-approved/2024
	CHECKMATE-77T Phase III (NCT04025879)^203^	Nivolumab + Chemotherapy	Nivolumab + Chemotherapy vs. Placebo + Chemotherapy (neoadjuvant) and Nivolumab vs. Placebo (adjuvant)	IIA-IIIB (≥4 cm)	Regardless of PD-L1 levels	18-month EFS: 70.2% vs. 50.0%	FDA-approved/2024
	NEOTORCH Phase III (NCT04158440, China-only)^202^	Toripalimab + Chemotherapy	Toripalimab + Chemotherapy vs. Placebo + Chemotherapy (neoadjuvant) and Toripalimab vs. Placebo (adjuvant)	II-IIIB	Regardless of PD-L1 levels	EFS: not reached vs. 15.1 months MPR: 48.5% vs. 8.4% pCR: 24.8% vs. 1.0%	NMPA-approved/2024
	RATIONALE-315 Phase III (NCT04379635)^805^	Tislelizumab + Chemotherapy	Tislelizumab + Chemotherapy vs. Placebo + Chemotherapy (neoadjuvant) and Tislelizumab vs. Placebo (adjuvant)	II-IIIA	Regardless of PD-L1 levels	MPR: 56.0% vs. 15.0% pCR: 41.0% vs. 6.0%	NMPA-approved/2024
	NADIM Phase II, Single-arm (NCT03081689)^196,242^	Nivolumab + Chemotherapy	Nivolumab + Chemotherapy (neoadjuvant) and Nivolumab (adjuvant)	IIIA	Regardless of PD-L1 levels	24-month PFS: 77.1% 5-year PFS: 65.0% 5-year OS: 69.3%	
	NADIMII Phase II (NCT03838159)^197^	Nivolumab + chemotherapy	Nivolumab + Chemotherapy vs. Placebo + Chemotherapy (neoadjuvant) and Nivolumab vs. Placebo (adjuvant)	IIIA/IIIB	Regardless of PD-L1 levels	pCR: 37.0% vs. 7.0%	
	SAKK16/14 Phase II, Single-arm (NCT02572843)^245^	Durvalumab + Chemotherapy	Chemotherapy and durvalumab sequentially (neoadjuvant), and Durvalumab(adjuvant)	IIIA(N2)	Regardless of PD-L1 levels	1-year EFS: 73.0%	
	TD-NeoFOUR Phase II, Single-arm (NCT05400070)^806^	Sintilimab + Anlotinib + Chemotherapy	Sintilimab + Anlotinib + Chemotherapy (neoadjuvant) and Sintilimab (adjuvant)	III	Regardless of PD-L1 levels	pCR: 57.8% MPR: 66.7%	
Combined curative chemoradiotherapy	Phase II, Single-arm (NCT03102242)^807^	Atezolizumab	Atezolizumab followed by Chemoradiotherapy + consolidated Atezolizumab	Unresectable III	Regardless of PD-L1 levels	Disease control rate at 12 weeks: 74.2% PFS: 30.0 months	
Neoadjuvant therapy	PRINCEPS Phase II, Single-arm (NCT02994576)	Atezolizumab	Atezolizumab	I-IIIA	Regardless of PD-L1 levels	Rate of without major toxicities within 1 month post-surgery: 10.0%	
	CHECKMATE-159 Phase II, Single-arm (NCT02259621)^247^	Nivolumab	Nivolumab	I-IIIA	Regardless of PD-L1 levels	MPR: 45.0%	
	IONESCO (IFCT-1601) Phase II, Single-arm (NCT03030131)^808^	Durvalumab	Durvalumab	IB (≥4 cm)-IIIA	Regardless of PD-L1 levels	Complete resection rate: 89.0%	
	Phase Ib, Single-arm (CHICTR-OIC-17013726)^809^	Sintilimab	Sintilimab	I-IIIB	Regardless of PD-L1 levels	AEs: 52.5% ≥grade 3 AEs: 10.0%	
	CHECKMATE-816 Phase III (NCT02998528)^191^	Nivolumab + Chemotherapy	Nivolumab + Chemotherapy vs. Placebo + Chemotherapy	IB (≥4 cm)-IIIA	Regardless of PD-L1 levels	EFS: 31.6 months vs. 20.8 months pCR: 24.0% vs. 2.2%	FDA-approved/2022
	Phase II, Single-arm (NCT02716038)^200^	Atezolizumab + Chemotherapy	Atezolizumab + Chemotherapy	IB-IIIA	Regardless of PD-L1 levels	MPR: 57%	
	Phase II, Single-arm (CHICTR1900023758)^199^	Sintilimab + Chemotherapy	Sintilimab + Chemotherapy	IIIA	Regardless of PD-L1 levels	Grade 3 to 5 AEs: 8.0% MPR: 43.3%	
	NEOPREDICT-LUNG Phase II (NCT04205552)^215^	Nivolumab + Relatlimab	Nivolumab + Relatlimab vs. Nivolumab	IB-IIIA	Regardless of PD-L1 levels	Curative resection rate: 95.0%	
	EAST ENERGY Phase II, Single-arm (NCT04040361)^810^	Pembrolizumab + Ramucirumab	Pembrolizumab + Ramucirumab	IB-IIIA	PD-L1 ≥ 1%	MPR: 50.0%	
	NEOSTAR Phase II (NCT03158129)^688^	Nivolumab + Ipilimumab + Chemotherapy	Nivolumab + Ipilimumab + Chemotherapy vs. Nivolumab + Chemotherapy	I-IIIA	Regardless of PD-L1 levels	MPR: 50.0% vs. 32.1%	
	INCREASE Phase II, Single-arm (NCT04245514)^811^	Ipilimumab + Nivolumab + Chemotherapy	Ipilimumab + Nivolumab + Chemotherapy + Radiotherapy	II-IIIB	Regardless of PD-L1 levels	MPR: 63.0% pCR: 50.0%	
	NEOCOAST Phase II (NCT03794544)^812^	Durvalumab, Oleclumab (anti-CD73), Monalizumab (anti-NKG2A) or Danvatirsen (anti-STAT3)	Durvalumab + Oleclumab vs. Durvalumab + Monalizumab or vs. Durvalumab + Danvatirsen vs. Durvalumab alone	IA3-IIIA	Regardless of PD-L1 levels	MPR: 19.0% vs. 30.0% vs. 31.3% vs. 11.1%	
Neoadjuvant targeted therapy	Phase II, Single-arm (NCT03433469)^813^	Osimertinib	Osimertinib	I-IIIA	EGFR L858R or exon 19 deletion	MPR: 14.8% ORR: 52% DFS: 40.9 months	
Adjuvant therapy	IMPOWER010 Phase III (NCT02486718)^246^	Atezolizumab	Atezolizumab vs. Best supportive care	IB (≥4 cm)-IIIA	PD-L1 ≥ 1%	3-year DFS: 60.0% vs. 48.2%	FDA-approved/2021
	KEYNOTE-091/PEARILS Phase III (NCT02504372)^228^	Pembrolizumab	Pembrolizumab vs. Placebo	IB-IIIA	Regardless of PD-L1 levels	DFS: 53.6 months vs. 42.0 months	FDA-approved/2023
	Phase II Single-arm^814^	Pembrolizumab	Pembrolizumab for up to 2 years following complete resection and neoadjuvant CCRT	IIIA/N2	Regardless of PD-L1 levels	DFS: 22.4 months year DFS: 29.0% 2-year OS: 86.0%	
Adjuvant targeted therapy	ADAURA Phase III (NCT02511106)^815^	Osimertinib	Osimertinib + Chemotherapy vs. Placebo + Chemotherapy	IB (3–5 cm)-IIIA	EGFR 19del/L858R	year DFS: 70.0% vs. 29% (II-IIIA) 4-year DFS: 73.0% vs. 38.0% (IB-IIIA)	FDA-approved/2020
	EVIDENCE Phase III (NCT02448797)^816^	Icotinib	Icotinib vs. Chemotherapy	II-IIIA	EGFR 19del/L858R	DFS: 47.0 months vs. 22.1 months	NMPA-approved/2021
	ALINA Phase III (NCT03456076)^225^	Alectinib	Alectinib vs. Chemotherapy	IB (≥4 cm)-IIIA	ALK Positive	2-year DFS: 93.8% vs 63.0% (II-IIIA); 93.6% vs 63.7% (IB -IIIA)	FDA-approved/2024
Adjuvant immunotherapy after CCRT	PACIFIC Phase III (NCT02125461)^817^	Durvalumab	Durvalumab vs. Placebo	III	Regardless of PD-L1 levels	PFS: 16.9 months vs. 5.6 months OS: 47.5 months vs. 29.1 months	FDA-approved/2018
	GEMSTONE-301 Phase III (NCT03728556)^818^	Sugemalimab	Sugemalimab vs. Placebo	III	Regardless of PD-L1 levels	PFS: 9.0 months vs. 5.8 months	NMPA-approved/2022
	KEYNOTE-799 Phase II (NCT03631784)^819^	Pembrolizumab + Chemotherapy	Cohort A (squamous/non-squamous): Pembrolizumab + Chemotherapy (dose fractionation) + Radiotherapy and Pembrolizumab Cohort B (non-squamous): Pembrolizumab + Chemotherapy + Radiotherapy and Pembrolizumab	IIIA-C	Regardless of PD-L1 levels	ORR: 70.5%vs. 70.6% Grade 3 to 5 pneumonitis: 8.0% vs. 6.9%	
Adjuvant targeted therapy after CCRT	LAURA Phase III (NCT03521154)^214^	Osimertinib	Osimertinib vs. Placebo	Unresectable III	EGFR L858R or exon 19 deletion	PFS: 39.1 months vs. 5.6 months	FDA-approved/2024
Perioperative therapy	IMPOWER030 Phase III (NCT03456063)	Atezolizumab + Chemotherapy	Atezolizumab + Chemotherapy vs. Placebo + Chemotherapy (neoadjuvant) and Atezolizumab vs. Placebo (adjuvant)	II-IIIB	Regardless of PD-L1 levels	EFS, MPR	ongoing
	Phase III (NCT05157776)	Sintilimab + Chemotherapy	Sintilimab + Chemotherapy vs. Placebo + Chemotherapy (neoadjuvant) and Sintilimab + chemotherapy vs. Placebo (adjuvant)	IIIA	Regardless of PD-L1 levels	pCR	ongoing

*PFS* progression free survival, *AEs* adverse events, *OS* overall survival, *FDA* Food and Drug Administration U.S., *NMPA* National Medical Products Administration, China, *ORR* objective response rate, *DFS* disease free survival, *EFS* event free survival, *MPR* major pathological response, *pCR* pathological complete response, *CCRT* concurrent chemoradiotherapy

### Local curative therapy

The selection of a surgical strategy for early-stage NSCLC is a collaborative decision influenced by both the individual patient’s characteristics and the surgeon’s clinical judgement. According to the AJCC 8th Edition classification, patients with resectable lesions who may benefit from neoadjuvant therapy include: primary tumors with a diameter of 1–5 cm (T1, T2a, T2b), accompanied by involvement of single or multiple N2 lymph nodes (stage IIIA), primary tumors with a diameter of 5–7 cm (T3), accompanied by involvement of N1 lymph nodes (stage IIIA), primary tumors larger than 7 cm (T4), accompanied by involvement of N0 or N1 lymph nodes (stage IIIA), or primary tumors with a diameter of 5–7 cm (T3), accompanied by involvement of N2 lymph nodes (stage IIIB). For selected T4N0 patients (based on size or proximity to anatomic structures involved), where surgical resection is feasible from both a medical and surgical perspective following a multidisciplinary assessment, surgical intervention may be provided.^207^

Typically, surgical approaches are executed by posterior lateral thoracotomy with priority on muscle-sparing rib expansion, or more minimally invasive techniques such as video-assisted thoracic surgery (VATS) or robotic-assisted thoracoscopic surgery (RATS), being that – these minimally invasive procedures can result in milder postoperative pain, fewer complications and shorter hospital stays.^208^ With the advancement of surgical techniques, and the integration of enhanced optical or near-infrared imaging and AI, more intricate and pre-inductive therapy cases can be addressed through comprehensive tumor removal using minimally invasive methods. As observed in recent years, the use of VATS and RATS has significantly increased, from 28% and 1.0% in 2008 to 40.5% and 16.9% in 2015, respectively,^209^ while the proportion of open lobectomies declined from 71% to 42.6%.

The recommendation for lobectomy as a first-choice treatment – based on evidence from the Lung Cancer Study Group comparing lobectomy to limited resection for stage I NSCLC in the 1980s, may not fully align with the current practices today. A variety of large database studies, meta-analyses, and retrospective institutional studies have demonstrated that sublobar resection (such as segmentectomy) might be the preferred surgical option for patients with compromised lung function or comorbidities,^210^ who are considered high-risk, whereas patients with tumors characterized by higher histological grading may be better suited for lobectomy.^211^ Given the increased risk of complications, the prevalent perspective on pneumonectomies is generally conservative, nevertheless, there is new data indicating that the need for a complete removal of lung should not be considered an absolute contraindication to surgical intervention, as shown in the CHECKMATE-816 trial where EFS rates were 67% in the experimental group versus 48% in the control group undergoing pneumonectomies.^191^ Another advancement is the implementation of enhanced recovery after surgery (ERAS) pathways or protocols, which are associated with reduced use of opioid medications, decreased fluid overload, and fewer cardiovascular and pulmonary complications.^212^

For patients with medically or surgically inoperable stage I-IIINSCLC presenting in good performance status (PS), concurrent chemoradiation therapy is considered the standard therapy and preferred over sequential treatment modalities^213^ which only is provided to individuals who are not suitable candidates for concurrent therapy, followed by the administration of durvalumab consolidation therapy for the subsequent 12-month period. For unresectable stage III NSCLC with EGFR mutations, osimertinib treatment after chemoradiation significantly prolonged PFS, yet there was no notable difference in 3-year overall survival rates.^214^ Currently, various alternative strategies, such as single-agent and dual-agent ICIs,^215^ or ICIs combined with anti-angiogenic drugs, used either concurrently^216–218^ or sequentially^218,219^ with radiation therapy alone or concurrent chemoradiotherapy, are under investigation to determine if these treatments bring about benefits.

Patients with stage III NSCLC undergoing concurrent chemoradiotherapy typically receive a dose of 60 Gy, where the incremental radiation does not yield additional benefits, furthermore, immunotherapy may potentially address the limitations of this radiation dose-insufficient patient subgroup. Remarkably, for specific patients, consideration may be given to administering doses above 60 Gy, with special caution to manage the radiation to the heart, lungs, and esophagus.^220^

### Adjuvant therapy for NSCLC

Since 1995, both clinical trials and meta-analyses have shown that the addition of platinum-based chemotherapy leads to a mere 5% increase in 5-year OS in NSCLC patients with diseases at stagesIB, II, and IIIA.^221^ Currently, for stageIB-IIIA patients with sensitive EGFR mutations (Ex19 del or L858R), based on the ADAURA trial outcomes,^222^ three years of osimertinib adjuvant therapy is recommended, regardless of PD-L1 status. For more severe stages, such as IIA, IIB, and IIIA, adjuvant icotinib which is only approved in China,^223^ or chemotherapy followed by osimertinib treatment^190,224^ is also advised to provide, however, based on the subgroup results, this combination therapy did not significantly improve OS. Furthermore, in patients with fully resected in stage IIIA NSCLC, the introduction of third-generation EGFR-TKI osimertinib did not yield significantly greater survival benefits compared to first-generation drugs. For ALK-positive patients, in the ALINA trials alectinib showed a significant enhancement in disease-free-survival (DFS) compared to platinum-based chemotherapy (Table 1),^225^ furthermore, the comprehensive OS benefits remained to be fully uncovered with extended follow-up periods. The data for adjuvant targeted therapies against other actionable oncogenic drivers such as ROS-1 and RET mutations is limited, suggesting a need for large-scale phase III clinical trials to validate their efficacy.

For resectable NSCLC patients with non-driver mutation and PD-L1 expression above 1%, adjuvant chemotherapy followed by atezolizumab treatment is recommended^226^ (the recommendation is referenced from the 7th edition lung cancer staging in the study, rather than the current 8th edition), grounded on the IMpower-010 trial which was the first to suggest that post-surgical adjuvant immunotherapy improves DFS, although the majority of benefits appear to stem from patients with PD-L1 expression above 50%.^227^ Similarly, the recent phase III PEARLS/KEYNOTE-091 trial was the only one to demonstrate that pembrolizumab as adjuvant therapy following surgical operation significantly improves overall survival, regardless of PD-L1 expression, however, subgroup analyses failed to show more pronounced benefits in the group with PD-L1 expression greater than 50%.^228^ Therefore, for patients with PD-L1 levels ≥50%, treatment with atezolizumab may be prioritized, although the mechanism behind the contrasting conclusions on the two drugs remains unclear. Following concurrent chemoradiotherapy, durvalumab or sugemalimab consolidation therapy is also a standard option for non-surgical patients.^229^ Multiple ongoing trials are assessing the impact of adding ICIs to adjuvant chemotherapy after complete resection of NSCLC,^230,231^ yet they have yielded mixed outcomes.

Postoperative radiation therapy (PORT) for patients with completely resected stage III or N2 disease remains controversial. In 2007, the ANITA study suggested that PORT was linked to enhanced survival rates among patients with stageIB-IIIA NSCLC who had undergone complete resection.^226^ However, preliminary findings from the recent randomized trial suggest that for N2 NSCLC patients who underwent complete resection, the therapy with PORT did not demonstrate a significant advantage over that without PORT in terms of DFS (47.1% vs 43.8%) or OS rate (66.5% vs 68.5%).^232,233^ Consequently, adjuvant radiation therapy is only recommended under circumstances where the risk of local recurrence is considered, particularly following R1 resection.

### Evaluation of therapeutic outcomes

While accurate long-term survival prediction is crucial for effective treatment, in clinical trials, assessing the efficacy of neoadjuvant therapy relies on surrogate endpoints – various imaging and molecular pathology or stage-based methods for evaluating tumor response,^200^ all of which may not strongly correlate with survival. Furthermore, in treatments with ICIs, patients might experience the phenomenon of initial tumor growth (pseudoprogression),^234^ but still show significant improvement in OS rather than PFS.

The pathology reporting should follow the multidisciplinary recommendations of the IASLC for evaluating resected lung specimens after neoadjuvant therapy, taking into account whether the patient has received neoadjuvant therapy and the type of treatment, the presence of multiple tumors within the sample (correctly annotating the lobes or multiple lobes removed), and whether there is involvement of structures such as the pericardium, diaphragm, or chest wall.^235^ Major pathologic response (MPR), defined as having less than 10% residual viable tumor area, has been suggested as an alternative endpoint for predicting survival in patients treated with neoadjuvant chemotherapy, potentially indicating better survival outcomes.^190,192^ However, the optimal threshold for remaining viable tumor may vary by histopathological type.^236^

An increasing body of evidence suggests that real-time monitoring approaches based on ctDNA can track intratumoral heterogeneity, evolution, and response to therapy, particularly in patients who are likely to harbor dominant resistance mutations.^237^ Indeed, ctDNA is increasingly being utilized to monitor for the presence of MRD in NSCLC patients after surgery or chemoradiotherapy followed by ICIs,^238^ exemplified as ctDNA positivity is capable of signaling the disease recurrence approximately 5.2 months prior to the appearance of radiological evidence, thus enabling the duration of adjuvant treatment to be gauged.^239,240^ Other researchers have also reported that multi-parametric approaches, such as the combination of genomic and epigenomic sequencing of ctDNA along with barcode techniques, and the integration of ctDNA from both germline and somatic mutations, could enhance the sensitivity of liquid biopsy methods. However, not all tumors secrete ctDNA, and current detection technologies might not be sufficiently sensitive in their depth of detection,^140^ on the contrary, high sensitivity can lead to false positive detection results.^241^

Regarding the relationship between PD-L1 and treatment efficacy, unlike that in the later-stage setting, opinions in the neoadjuvant setting are divided. In trials such as NADIM,^242^ NADIM II,^197^ LCMC3,^243^ NEOSTAR,^244^ Sakk 16/14,^245^ and CHECKMATE-816,^191^ higher levels of PD-L1 are enriched in patients who achieve a pathological complete response (pCR), however, PD-L1 levels showed no correlation with PFS or OS (seen in NADIM and Sakk 16/14). In an adjuvant setting, the IMpower-010 trial demonstrated an improvement in DFS among patients in stages II to IIIA with a tumor proportion score (TPS) for PD-L1 greater than 1%, with this group showing a significant benefit particularly when PD-L1 exceeded 50%.^246^ Contrary to that, there have been studies indicating no significant association between PD-L1 and MPR.^247^ Furthermore, the PEARLS trial recently showed in the subgroup analysis that there was no significant benefit from the experimental treatment for the subpopulation with PD-L1 levels greater than 50%.^228^

Similarly, like PD-L1, the value of TMB as a biomarker may be limited.^191,242^ Following neo-adjuvant immunotherapy, an increase in T-cell clonality or special TIME subtypes^248^ has been observed to be correlated with a lower percentage of residual tumor at surgery, supported by the NEOSTAR^244^ and NADIM study^249^ findings. Analogously, in the SAKK 16/14 study, T-cell receptor clonality in preoperative peripheral blood samples, rather than preoperative tissue samples, was associated with one-year EFS amelioration.^250^ Regarding the above trial data, several key issues should be considered, such as patient heterogeneity caused by the TNM staging edition, inclusion criteria (including EGFR or ALK mutations), and the threshold definition for PD-L1 expression, and the heterogeneity of efficacy outcomes defined by EFS, DFS, pCR, MPR, and OS.^251^

### Challenges

The therapies for non-advanced NSCLC, in particular stage III and characterized as marked heterogeneity, still face significant challenges due to the absence of randomized trials defining treatment for the entire III stage disease spectrum. Herein, we focus on several predominant aspects that we deem important.

Firstly, patient selection for surgical or non-surgical treatments has not been clearly defined based on high-quality evidence.^252^ The INT0136 trial and subsequent retrospective studies defined N2 disease as unsuitable for surgery, particularly in cases of multicentric metastases. Nevertheless, the progress made in chemoimmunotherapy and minimally invasive surgical techniques has empowered surgeons to apply a more pronounced curative intent in treating unresectable stage III diseases, supported by a recent study with a 25% conversion rate to surgery.^253^ Therefore, positing that only N2 cases featuring solitary lymph node metastases are suitable for surgical intervention would be an oversimplification.

Secondly, the relationship between definitive chemoradiotherapy and surgery is not entirely adversarial.^252,254^ Considering the strikingly positive clinical outcomes in 54% of patients with multifocal N2 disease from the NADIM trial, a question has arisen on whether surgery is needed post-chemoimmunotherapy. In early studies of neoadjuvant and perioperative immunotherapies, up to 20% of patients may not undergo surgery due to disease progression, treatment-induced worsening, or unresectable tumors.^198,255^ Therefore, for both scenarios, PD-L1-positive patients might consider either radiotherapy alone^256^ or concurrent chemoradiotherapy, followed by durvalumab or other ICIs as consolidation therapy, based on the results of trials like PACIFIC (Fig. 5).^229,257^

Thirdly, when it comes to determining whether adjuvant or neoadjuvant chemotherapy yields a higher survival benefit, a meta-analysis making an indirect comparison indicated that both approaches have a comparable effect on survival rates (a 4–5% absolute increase at 5 years). Furthermore, whether perioperative chemotherapy through combining both approaches leads to more pronounced benefits is unclear, although such a practice has been occasionally adopted in clinical settings, particularly for patients with locally advanced disease.

Fourthly, the sequence of immunotherapy – whether patients should undergo neoadjuvant chemoimmunotherapy followed by surgery (or radiotherapy) or vice versa, then followed by adjuvant chemotherapy or ICIs – is still not entirely clear. The multiple trials^242^ have demonstrated that neoadjuvant immunotherapy, compared to adjuvant immunotherapy, may have better correlation with better pathological responses and survival,^242,244,247^ where the pathological complete response rate was 24% in the chemoimmunotherapy group, significantly higher than only 2.2% in the chemotherapy group.^258^ However, contrary to this, trials such as the IMpower-010 and KEYNOTE-091 trials support the approach of upfront surgery followed by adjuvant chemotherapy and one year of adjuvant immunotherapy, which also holds promise in terms of curative potential^228,246^ (Fig. 5).

A further issue is that how to select a postoperative adjuvant therapy, such as immunotherapy alone or combined with chemotherapy or observation when surgery is selected following neoadjuvant chemoimmunotherapy, although indirect comparisons of the efficacy data from the neoadjuvant CHECKMATE-816 trials and the perioperative CHECKMATE-77T trials suggest that administering adjuvant immunotherapy may not result in clinical benefits.^191^ Overall, non-pCR patients with PD-L1 expression below 1% may not require adjuvant immunotherapy. Furthermore, what constitutes an optimal duration for adjuvant immunotherapy is not yet clear. Phase III trials typically offer one year of adjuvant ICI treatment,^259^ though compliance rates are often lower.^201,246^ In contrast, shorter durations of adjuvant therapy, such as for six-month period investigated in the NADIM II trial,^197^ could provide comparable efficacy with fewer side effects. Undeniably, these inevitable controversies highlight the critical need to identify precise molecular biomarkers and the role of multidisciplinary tumor boards.^189^

Another concerning issue is the lack of clear guidance on neoadjuvant treatment for patients with driver mutations. For potentially curable patients, neoadjuvant TKIs can be considered, based on extrapolation from ADAURA trial. Although the pCR rate may be low, it still creates the possibility of a cure. However, neoadjuvant treatments that include ICIs are not recommended, based on subgroup analyses from AEGEAN trial.

Lastly, oncologists treating patients with ICIs (whether in combination with chemotherapy or not), should be vigilant on the potential long-term side effects of ICIs, particularly the cardiovascular side effects in elderly patients or reproductive impairment in young patients.^260^ This also highlights the imperative requirement for a comprehensive strategy that integrates both clinical data and multi-omics profiling to improve individual responsiveness to immunotherapeutic interventions.

## Systemic therapy for advanced or metastatic NSCLC

For advanced-stage NSCLC, a complex continuum, treating patients when tumor burden and clonal heterogeneity are low is considered a wise strategy, as this may facilitate easier tumor elimination and potentially prevent drug resistance.^261^ Specifically, employing more potent, tailored therapies as first-line treatments could help extend survival. Additionally, treatment efficacy can be enhanced by using drugs with a high therapeutic index (Table 2), optimizing dosages and timing, and combining them with other drugs that have distinct but complementary pharmacological actions.^261^ Another approach to improving response efficacy involves early monitoring of the dynamic and global clonal evolution of cancer cells,^262^ which can allow for the timely identification of patients developing resistance.

**Table 2:** Frontline targeted therapies for advanced-stage NSCLC

Targets	Trial names	Assessed regimes	Trial interventions	Primary endpoints	Regulatory status
EGFR (High therapeutic index)	IDEAL 1 IDEAL 2 Phase II^820^	Gefitinib (ZD1839)	Gefitinib 500 mg vs. 250 mg	PFS: 2.8 months vs 2.7 months OS: 8.0 months vs 7.6 months PR: 19.0% vs 18.4%	Japan-approved/2002 FDA-approved/2003 (later-line, irrespective of EGFR mutations)
	IPASS WJTOG3405 Phase III (NCT00322452) (UMIN000000539)^821^	Gefitinib	Gefitinib vs. Chemotherapy	month PFS: 24.9% vs. 6.7% (IPASS) PFS: 9.2 months vs. 6.3 months (WJTOG3405)	FDA-approved/2015 (first-line, EGFR mutations based on WJTOG3405)
	EURTAC Phase III (NCT00446225)^822^	Erlotinib	Erlotinib vs. Chemotherapy	PFS: 9.7 vs. 5.2 months	FDA-approved/2013 FDA-approved/2004 (later-line)
	LUX-LUNG 3 LUX-LUNG 6 Phase III (NCT00949650)^823^ (NCT01121393)	Afatinib	Afatinib vs. Chemotherapy	PFS: 11.7 months vs. 6.9 months OS: 28.2 months vs. 28.2 months (LUX-LUNG 3) PFS: 11.0 months vs. 5.6 months OS: 23.1 months vs. 23.5 months (LUX-LUNG 6)	FDA-approved/2013 FDA-approved/2018 (extending to uncommon EGFR mutations)
	LUX-LUNG 8 Phase III (NCT01523587)^824^	Afatinib	Afatinib vs. Erlotinib	OS: 7.8 months vs. 6.8 months	FDA-approved/2016(squamous NSCLC progression after chemotherapy)
	ARCHER 1050 Phase III (NCT01774721)^273^	Dacomitinib	Dacomitinib vs. Gefitinib	PFS: 14.7 months vs. 9.2 months OS: 34.1 months vs. 27.0 months	FDA-approved/2018
	FLAURA Phase III (NCT02296125)^279^	Osimertinib	Osimertinib vs. Gefitinib	PFS: 18.9 months vs. 10.2 months	FDA-approved/2018
	FLAURA2 Phase III (NCT04035486)^280^	Osimertinib + Chemotherapy	Osimertinib + Chemotherapy vs. Osimertinib	PFS: 25.5 months vs. 16.7 months	FDA-approved/2024
	MARIPOSA Phase III (NCT04487080)^293^	Amivantamab + Lazertinib	Amivantamab + Lazertinib vs. Osimertinib	PFS: 23.7 months vs. 16.6 months	FDA-approved/2024
	CONVINCE Phase III (NCT01719536)^825^	Icotinib	Icotinib vs. Chemotherapy	PFS: 11.2 months vs. 7.9 month	NMPA-approved/2011
	AENEAS Phase III (NCT03849768)^281^	Aumolertinib	Aumolertinib vs. Gefitinib	PFS:19.3 months vs. 9.9 months	NMPA-approved/2021
	FURLONG Phase III (NCT03787992)^283^	Furmonertinib	Furmonertinib vs. Gefitinib	PFS: 20.8 months vs. 11.1 months	NMPA-approved/2022
	IBIO 103 Phase III (NCT04206072)^285^	Befotertinib	Befotertinib vs. Icotinib	PFS: 22.1 months vs. 13.8 months	NMPA-approved/2023
	BPI-7711-2015-001 Phase IIa (NCT03386955)^826,827^	Rezivertinib	Rezivertinib	ORR: 83.7% DOR: 19.3 months PFS: 20.7 months	NMPA-approved/2024
	LASER 201 Phase I/II (NCT03046992)^828^	Lazertinib	Lazertinib (EGFR T790M mutations)	ORR: 57.9% DCR: 89.5% PFS: 13.2 months OS: 38.9 months	Southern Korean-approved/2021 (second-line)
	LASER 301 Phase III (NCT04248829)^284^	Lazertinib	Lazertinib vs. Gefitinib	PFS: 20.6 months vs. 9.7 months	
	SHC013-III-01 Phase III (NCT04239833) (2024 WCLC. OA02.04.)	Rilertinib	Rilertinib vs. Gefitinib	PFS 19.3 months vs. 9.8 months	NMPA-approved/2024
ALK (High therapeutic index)	PROFILE 1014 Phase III (NCT01154140)^829^	Crizotinib	Crizotinib vs. Chemotherapy	PFS: 10.9 months vs. 7 months OS: not reached vs. 47.5 months	FDA-approved/2011
	ALEX Phase III (NCT02075840)^830^	Alectinib	Alectinib vs. Crizotinib	PFS: 34.8 months vs. 10.9 months OS: not reached vs. 57.4 months	FDA-approved/2017 FDA-accelerated approval/2015 (later-line)
	ASCEND-4 Phase III (NCT01828099)^302^	Ceritinib	Ceritinib vs. Chemotherapy	PFS: 16.6 months vs. 8.1 months	FDA-approved/2017 FDA-accelerated approval/2014 (later-line)
	CROWN Phase III (NCT03052608)^308^	Lorlatinib	Lorlatinib vs. Crizotinib	year PFS: 60% vs. 8% PFS: not reached vs. 9.1months	FDA-approved/2021 FDA-accelerated approval/2018 (later-line)
	ALTA-1L Phase III (NCT02737501)^831^	Brigatinib	Brigatinib vs. Crizotinib	PFS: 24.0 months vs. 11.0 months	FDA-approved/2020
	eXalt3 Phase III (NCT02767804)^306^	Ensartinib	Ensartinib vs. Crizotinib	PFS: 25.8 months vs. 12.7 months	NMPA-approved/2022 FDA approved/2024
	INSPIRE Phase III (NCT04632758)^832^	Iruplinalkib	Iruplinalkib vs. Crizotinib	PFS: 27.7 months vs. 14.6 months	NMPA-approved/2023
	TQ-B3139-III-01 Phase III(NCT04009317)^305^	Envonalkib	Envonalkib vs. Crizotinib	PFS: 24.9 months vs. 11.6 months ORR: 81.7% vs. 70.7%	NMPA-approved/2024
ROS1 (High therapeutic index)	PROFILE 1001 Phase I (NCT00585195)^50^	Crizotinib	Crizotinib	ORR: 72.0% PFS: 19.2 months	FDA-approved/2016
	STARTRK-1 and 2 ALKA-372-001 Phase I/II (NCT02568267) (NCT02097810) (EndraCT, 2012-000148-88)^310^	Entrectinib	Entrectinib	ORR: 78.0% DOR: 24.6 months	FDA-approved/2019
	TRIDENT-1 Phase I/II (NCT03093116)^311^	Repotrectinib	Repotrectinib	ORR: 79.0% DOR: 34.1 months PFS: 35.7 months	FDA-approved/2023
	TQ-B3101-1-0001 Phase I/II (NCT03019276)^313^	Unecritinib	Unecritinib	ORR: 80.2% PFS: 16.5 months	NMPA-approved/2024
	TRUST-I Phase II (NCT04395677)^314^	Taletrectinib	Taletrectinib	DOR: 10.6 months PFS: 7.6 months	NMPA-approved/2024 (later-line)
	Phase I/II (NCT01970865)^312^	Lorlatinib	Lorlatinib	ORR: 62.0% (first-line) ORR: 35.0% (second-line)	
EGFR 20ins (Medium therapeutic index)	CHRYSALIS Phase I (NCT02609776)^301^	Amivantamab	Amivantamab	ORR: 40.0% DOR: 11.1 months PFS: 8.3 months	FDA-approved/2024 (later-line)
	PAPILLON Phase III (NCT04538664)^297^	Amivantamab + Chemotherapy	Amivantamab + Chemotherapy vs. Chemotherapy	PFS: 11.4 months vs. 6.7 months	FDA-approved/2024
	WU-KONG8 Phase II (NCT05712902)^401^	Sunvozertinib	Sunvozertinib	ORR: 61.0%	NMPA-approved/2023(later-line)
RET (Medium therapeutic index)	ARROW Phase I/II (NCT03037385)^833^	Pralsetinib	Pralsetinib	ORR: 78.0% DOR: 13.4 months (first-line) ORR: 63.0% DOR: 38.8 months (second-line)	FDA-approved/2023 (first-line)
	LIBRETTO-431 Phase III (NCT04194944)^329^	Selpercatinib	Selpercatinib vs. Chemotherapy +/- Pembrolizuma	PFS: 24.8 months vs. 11.2 months	FDA-approved/2022
KRAS^G12C^ (Low therapeutic index)	KRYSTAL-1 Phase I/II (NCT03785249)^834^	Adagrasib	Adagrasib	ORR: 43.0% DOR: 8.5 months OS: 12.6 months	FDA-accelerated approval/2022(later-line)
	CodeBreaK100 Phase II (NCT03600883)^835^	Sotorasib	Sotorasib	ORR: 36.0% DOR: 11.1 months OS: 12.5 months	FDA-accelerated approval/2021(later-line)
	GFH925X1101 Phase I/II (NCT05005234)^836^	Fulzerasib	Fulzerasib	ORR: 49.1% DCR: 90.5%	NMPA-approved/2024 (later-line)
BRAF^V600E^ (Low therapeutic index)	BRF113928 Phase II (NCT01336634)^837^	Dabrafenib + Trametinib	Dabrafenib + Trametinib	ORR: 64.0%	FDA-approved/2017
	PHAROS Phase II (NCT03915951)^318^	Encorafenib + Binimetinib	Encorafenib + Binimetinib	ORR: 75.0% (first-line) and 46% (second-line) PFS: NE (first-line) and 9.3 months (second-line)	FDA-approved/2023
	Phase II (NCT02304809)^319^	Vemurafenib	Vemurafenib	ORR: 44.9% PFS: 5.2 months OS: 10.0 months	
NTRK fusion (Low therapeutic index)	STARTRK-1 and 2 ALKA-372-001 Phase I/II (NCT02568267) (NCT02097810) (EndraCT,2012-000148-88)^310,838^	Entrectinib	Entrectinib	ORR: 57.0% DOR: 10.0 months	FDA-accelerated approval/2019 (later- line)
	LOXO-TRK-14001 SCOUT NAVIGATE Phase I/II (NCT02122913) (NCT02637687) (NCT02576431)^839^	Larotrectinib	Larotrectinib	ORR: 75.0%	FDA-accelerated approved/2018 (later- line)
	TRIDENT-1 Phase I/II (NCT03093116)	Repotrectinib	Repotrectinib	ORR: 58.0% (first-line) or 50.0% (second-line)	FDA-accelerated approved/2024 (later- line)
MET 14 skipping (Low therapeutic index)	GEOMETRY Mono-1 Phase II (NCT02414139)^57^	Capmatinib	Capmatinib	ORR: 68.0% DOR: 12.6 months	FDA-approved/2022
	VISION Phase II (NCT02864992)^323,840^	Tepotinib	Tepotinib	ORR: 57.0% DOR: 46.4 months	FDA-approved/2024
	2016-504-00CH1 Phase II (NCT02897479)^324^	Savolitinib	Savolitinib	ORR: 49.2%	NMPA-approved/2021 (later-line)
	Phase IIIb^841^ (NCT04923945)	Savolitinib	Savolitinib	ORR: 62.0%	
	Glory Phase Ib/II (NCT04270591)^325^	Glumetinib	Glumetinib	ORR: 66.0%	NMPA-approved/2023
	KUNPENG Phase II (NCT04258033)^326^	Vebreltinib	Vebreltinib	ORR: 75.0% DOR: 15.9 months OS: 20.7 months	NMPA-approved/2023
	METROS Phase II (NCT02499614)^842^	Crizotinib	Crizotinib	ORR:27.0% PFS: 4.4 months OS: 5.4 months	
HER-2 (Low therapeutic index)	DESTINY-LUNG02 Phase II (NCT04644237)^181,333^	Trastuzumab + deruxtecan	Trastuzumab + deruxtecan	ORR: 58.0% DOR: 8.7 months	FDA-accelerated approval/2022 (second-line)
	CTONG1702 Phase II (NCT03574402)^576^	Pyrotinib	Pyrotinib	ORR: 35.7% PFS: 7.3 months OS: 14.3 months	
	ETOP NICHE Phase II (NCT02369484)^843^	Afatinib	Afatinib	PFS: 15.9 weeks OS: 56.0 weeks	
	Phase II (NCT00818441)^844^	Dacomitinib	Dacomitinib	OS: 9.0 months	
	Phase II (NCT02675829)^845^	Ado-Trastuzumab emtansine	Ado-Trastuzumab emtansine	ORR: 44.0% PFS: 5.0 months	
VEGF (Low therapeutic index)	RELAY Phase III (NCT02411448)^274^	Ramucirumab + Erlotinib	Ramucirumab + Erlotinib vs. Placebo+ Erlotinib	PFS: 19.4 months vs. 12.4 months DOR: 18.0 months vs. 11.1 months	FDA-approved/2020
	Phase III (NCT00021060)	Bevacizumab	Bevacizumab + Chemotherapy vs. Chemotherapy	PFS: 6.2 months vs. 4.5 months OS: 12.3 months vs. 10.3 months	FDA-approved/2006
NRG1 fusion (Low therapeutic index)	eNRGy Phase I/II (NCT02912949)^405^	Zenocutuzumab	Zenocutuzumab (anti-HER2xHER3 bispecific antibody)	ORR: 33% DOR: 7.4 months	FDA-accelerated approval/2024 (later-line)

The table includes important later-line treatment results, for instance, for EGFR mutation or NRG1 fusion

### Frontline targeted therapy

**Fig. 6:**
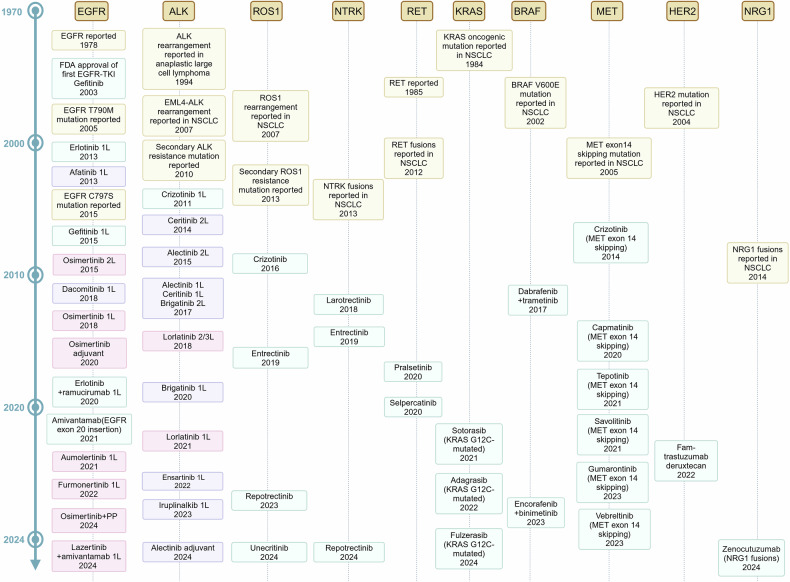
Timeline of the research history and milestone events in targeted therapy for NSCLC. The annotation times in this illustration refer to the initial FDA or NMPA approval dates for each drug, whether under accelerated or regular approval. This timeline showcases the significant advancements in NSCLC treatments over the last five decades, featuring nine targetable biomarkers and over 30 drugs. These developments represent some of the most impressive progress across all cancer types. While this growth underscores the advancement of personalized medicine, the abundance of data also presents challenges in treatment selection, especially when our knowledge is limited. It is important to note that our data sources, the FDA and NMPA, do not include drugs approved in other regions. Progress in drug iterations has been particularly notable for EGFR and ALK TKIs, while advancements in other targets have been less prominent, highlighting the challenges in developing treatments for less common targets, which continue to represent unmet clinical needs

For patients with advanced or metastatic NSCLC harboring specific oncogenic driver mutations such as ALK, ROS1, NTRK, and RET fusions, as well as mutations in HER-2, BRAF, KRAS, EGFR, and MET exon 14, frontline targeted therapies can be considered irrespective of PD-L1 levels (Fig. 6).^263^ Certainly, aiming to alleviate symptoms with early palliative treatments,^264^ such as nutritional support, radiotherapy, surgery, cryotherapy, microwave, or radiofrequency ablation, can enhance the quality of life in patients with metastatic NSCLC. In the event NGS outcomes are unclear or pending, patients may initially be given a cycle of platinum-based chemotherapy without immune medications to manage their symptoms, as the concurrent use of first to third-generation EGFR-TKIs or other TKIs alongside immunotherapy may fail to yield synergistic benefits and could potentially impose excessive toxicity on patients (Table 2).^265^

#### Targeting EGFR mutations

EGFR has long been recognized as a therapeutic target due to the significant upregulation of EGFR expression in NSCLC.^266^ Following the IPASS study, which demonstrated that gefitinib was superior to double-platinum chemotherapy, six randomized and controlled trials confirmed that the objective response rate (ORR) across various EGFR TKIs ranged from 62% to 83%, median PFS from 9.2 to 13.1 months, and median OS from 21.6 to 36 months. These findings solidified TKIs as the cornerstone of treatment for patients with classical sensitive EGFR mutations.^267–271^ While first-generation EGFR TKIs were initially approved for use in patients with EGFR overexpression, showing only mild activity, it was subsequently discovered that only those with EGFR mutations derive clinical benefit,^272^ which marked the advent of precision medicine. However, most patients still experience recurrence within a year, with the most common mechanism (60%) being the acquisition of the T790M mutation at the EGFR ATP binding site, which hinders the opportunity for erlotinib and gefitinib to bind. Second-generation irreversible pan-EGFR inhibitors, afatinib and dacomitinib,^273^ have been proven to be effective in suppressing the T790M mutation, despite receiving FDA approval as first-line treatments (owing to a longer median overall survival compared to first-generation EGFR TKIs). Multiple studies have indicated that as first-line treatment, TKIs combined with ramucirumab^274^ or bevacizumab (also FDA-approved biosimilar of bevacizumab^275,276^) are viable treatment options, albeit with little to no significant improvement in efficacy.^277^

Osimertinib, a third-generation irreversible EGFR TKI, exhibits greater potency and selectivity against both typical EGFR activating mutations and T790M resistance mutation. At present, osimertinib is recognized as the frontline standard of care for patients with advanced EGFR-mutated NSCLC (as illustrated in Fig. 1),^278^ supported by the pivotal FLAURA trial demonstrated that osimertinib prolongs median PFS (18.9 months vs. 10.2 months) and OS (38.6 months vs. 31.8 months), as compared to gefitinib or erlotinib.^279^ Although osimertinib is generally considered the first-line treatment option, in many countries, especially in areas where osimertinib is not reimbursed or unavailable, first and second-generation TKIs can still be used, and then switched to osimertinib when patients develop T790M resistance. We must acknowledge that NSCLC harboring EGFR mutations is a heterogeneous disease, where different patients may benefit from distinct therapeutic strategies.^280^ Following the positive outcomes of FLAURA, four additional third-generation EGFR TKIs were developed as alternative first-line treatment options for classical sensitive EGFR-mutated NSCLC, based on the AENEAS trial with aumolertinib,^281^ the FURLONG trial with furmonertinib,^282,283^ the LASER301 trial with lazertinib^284^ compared against gefitinib, and finally, befotertinib^285^ compared with another first-generation EGFR TKI, icotinib, with data showing significantly improved PFS (22.1 months vs.13.3 months). Notably, aumolertinib, furmonertinib, and befortinib have been exclusively studied in mainland China, and their efficacy indicators seem to align with those observed in the FLAURA study, although no direct comparison to osimertinib has been made. Other third-generation TKIs, including rezivertinib and rilertinib,^285^ are currently in development, showcasing encouraging outcomes.

For patients with less common EGFR mutations such as S768I, L861Q, and/or G719X (X represents multiple possible amino acids) in the context of advanced or metastatic NSCLC, first-line treatment failed to clearly establish until now yet, although afatinib or osimertinib alone or combined chemotherapy is the typical choice, akin to the standard of care for more prevalent mutations.^286,287^ However, retrospective data suggest that the clinical response to osimertinib and afatinib might vary depending on the precise EGFR mutation identified, for instance, NSCLC harboring the L861Q mutation, potentially favor Osimertinib for a better response.^288^

To enhance the activity of EGFR TKIs, several combinations have been evaluated as first-line therapy for EGFR-mutated advanced NSCLC. The first approach involves a combination with chemotherapy or ramucirumab,^289^ however, there are contradictory conclusions regarding the impact of combinations containing first-generation EGFR TKIs on OS. Recently, a phase III trial indicated that first-line gefitinib plus anlotinib acquired a median PFS of 14.8 months, compared to 11.2 months with gefitinib plus placebo (HR = 0.64, *P* = 0.003).^290^ FLAURA2 assessed the combination of osimertinib with chemotherapy in untreated patients with EGFR-mutated advanced NSCLC,^280^ who showed a significantly improved median PFS (25.5 months vs 16.7 months, *P* < 0.001) and intracranial PFS (30.2 months vs. 27.6 months, *P* = 0.05).^291^ Contrarily, a subgroup analysis from a larger randomized study conducted in Japan did not demonstrate superior efficacy when 3 cycles of chemotherapy were added to osimertinib compared to osimertinib alone.^292^ However, in the phase III MARIPOSA trial,^293^ first-line treatment with amivantamab-lazertinib demonstrated a significantly longer median PFS of 23.7 months compared to 16.6 months in the osimertinib group (*P* < 0.001), while OS results were immature at the current analysis, resulting in FDA approval on August 19, 2024, moreover, subcutaneous amivantamab also offers a consistent safety profile with increased convenience, and prolonged survival.^294^ MET overexpression has been found to predict responses to amivantamab and lazertinib in patients refractory to osimertinib,^295^ but whether this applies to the setting of untreated first-line treatment requires prospective validation. In addition, the addition of local radiotherapy may improve OS in specific patients with oligo-organ metastatic NSCLC.^296^

Typically, EGFR exon 20 insertions lead to reduced or lack of sensitivity to currently approved EGFR TKIs, with a median PFS duration not exceeding four months; however, there exist racial differences in this context, yet the A763_Y764insFQEA mutation stands out as an exception to this norm. Recently, amivantamab, a bispecific antibody targeting both EGFR and MET, in combination with chemotherapy has also received FDA approval as a first-line treatment for patients with exon 20 insertion, based on the findings of the phase III randomized trial, PAPILLON,^297^ which showed a longer median PFS in the combination group compared to the group with single-agent chemotherapy (11.4 months vs. 6.7 months, *P* < 0.001). High dose furmonertinib also shows promising clinical activity in a phase Ib trial. Mobocertinib is an EGFR TKI specifically designed for patients with EGFR exon 20 insertion mutations, previously receiving accelerated approval from the FDA.^298^ However, in 2023, mobocertinib was withdrawn from the US market based on the data from the phase III EXCLAIM-2 trial,^299^ which failed to validate the primary endpoint that first-line mobocertinib is superior to platinum-based chemotherapy. Similarly, platinum-based chemotherapy in the first line setting (such as carboplatin combined with paclitaxel± immunotherapy) rather than single ICIs,^300^ is recommended as a treatment option,^301^ but the response rates (0–25%) remained relatively low, and varied depending on the specific state of the 20 exon insertion mutation.

Currently, the front-line treatment landscape for advanced NSCLC harboring EGFR mutations is becoming increasingly congested, leading to treatment decisions more complex.^280^ Beyond the goal of extending survival, treatment recommendations should be personalized to strike a balance between the risk of increased toxicity from novel therapies and their potential benefits. Several adverse prognostic factors, such as brain metastasis, co-mutation with TP53, and high PD-L1 scores, may support intensified therapy, as demonstrated in trials like ACROSS 1 and 2 – which evaluate whether adding chemotherapy to aumolertinib in comparison to aumolertinib alone could improve PFS in tumors with co-mutations in TP53 or RB1.

#### Targeting ALK rearrangements

Crizotinib, approved by the FDA in 2011 for advanced NSCLC with ALK rearrangements, showed notable efficacy in I/II clinical trials with an ORR of about 60%, despite its initial development as a MET inhibitor. Subsequently, more potent second-generation ALK TKIs, including ceritinib,^302^ alectinib,^303^ brigatinib,^304^ envonalkib^305^ and ensartinib,^306^ had been developed to overcome resistance to crizotinib and enhance central nervous system (CNS) efficacy, thereby supplanting them as the standard first-line therapy for NSCLC patients harboring ALK rearrangements (often resulting in 12-month PFS rates of approximately 65%, compared to 45%).^304^

Lorlatinib, a third-generation, highly-effective, and selective ALK/ROS1 TKI against a broad range of ALK kinase domain-resistant mutations, and known for its significant central nervous system penetration,^307^ was granted accelerated FDA approval in 2018 for NSCLC patients with ALK rearrangements who have progressed on first-line alectinib, brigatinib, or crizotinib therapy. Following this, in March 2021, the FDA authorized the use of lorlatinib as first-line treatment for patients with advanced NSCLC, grounding this decision on findings from the recent phase III CROWN trial which revealed a 72% reduction in the risk of disease progression or death, alongside a markedly prolonged time to CNS progression (no CNS progression in 12 months, 96% vs. 60%) and more recently a 5-year PFS of 60% vs. 8%.^308^

#### Targeting ROS1 rearrangements

Although ROS1 is an independent receptor tyrosine kinase, several (though not all) targeted therapies designed for the treatment of ALK-positive metastatic NSCLC have also been recommended for use in the treatment of ROS1-positive metastatic diseases, like ceritinib, crizotinib, and lorlatinib^309^ with the median PFS ranged from 15.9 to 20 months across multiple trials. Updated results from the PROFILE 1001 study indicate that crizotinib achieved an objective response rate of 72%, with a median OS of 51.4 months. Entrectinib, an oral tyrosine kinase inhibitor, demonstrated efficacy in treating ROS1-positive metastatic NSCLC patients as first-line therapy in phases I or II of the STARTRK-2, STARTRK-1, and ALKA-372-001 trials,^310^ with a median DOR at 24.6 months and an intracranial overall response rate of 55%,^310^ surpassing crizotinib but at the cost of a higher incidence of adverse effects. In the TRIDENT-1 trial, repotrectinib, a next-generation tyrosine kinase inhibitor targeting ROS1, TRK, and ALK, demonstrated a median PFS of 35.7 months as first-line treatment for NSCLC patients, and a median PFS of 9 months for those who had previously been treated with crizotinib, entrectinib, or ceritinib (without chemotherapy).^311^ Among 69 patients with ROS1-positive metastatic NSCLC, lorlatinib demonstrated a higher first-line response rate of 62%, compared to a second-line response rate of 35%.^312^ Recently, unecritinib was approved in China as the first-line treatment of metastatic NSCLC harboring ROS1 rearrangement, based on promising results, among which the median PFS was 16.5 months (Table 2).^313^

Similarly, in the TRUST-I study,^314^ taletrectinib showcased superior efficacy in treating TKI-naïve patients, with the median DOR and PFS in crizotinib-pretreated patients recorded at 10.6 months and 7.6 months, respectively. Impressively, 67% (8 out of 12) of patients carrying the G2032R mutation responded to this treatment. Additionally, promising data have been reported for foritinib in a phase II study, particularly in those with CNS metastases.^315^

In summary, for ROS1-positive patients with metastatic NSCLC, the priority first-line treatments encompass entrectinib, taletrectinib, or repotrectinib, with crizotinib, ceritinib, and lorlatinib as alternative options. Particularly for those with brain metastases, entrectinib or repotrectinib might be more appropriate.

#### Targeting KRAS mutations

The prioritization of targeted therapy or chemoimmunotherapy (for specific details, see the section on immunotherapy) for treating patients with advanced NSCLC harboring KRAS^G12C^ mutations has not been clearly defined, however, PD-L1 levels can serve in guiding the selection of the appropriate first-line systemic treatment plan.^300^

For patients experiencing disease progression following first-line systemic therapy, who have not previously received KRAS^G12C^-directed treatments, sotorasib or adagrasib are subsequent treatment options, however, this recommendation does not apply to patients with mutations other than KRAS ^G12C^. The evidence is derived from a phase II study, which found that in KRAS^G12C^-positive patients after platinum-based chemotherapy ± immunotherapy, sotorasib resulted in a median OS of 12.5 months. A phase III clinical trial further revealed that the median PFS in the sotorasib group was 5.6 months, compared to 4.5 months in the docetaxel group (*P* = 0.0017), however, there was no significant difference reported in OS between the two groups. Another phase II study with adagrasib as a subsequent treatment, revealed a median OS of 12.6 months, with a brain response rate of 33.3%. However, sequential use of anti-PD-(L)1 and sotorasib therapy is associated with increased risks of significant hepatic toxicities and an elevation in the occurrence of non-hepatic severe adverse events, underscoring the necessity for a 30-day gap between treatments. However, early evidence has shown that the combination of adagrasib and pembrolizumab is a safe and effective regimen for newly diagnosed NSCLC harboring a KRAS^G12C^ mutation.^316^

#### Targeting BRAF mutations

For BRAF^V600E^-mutated NSCLC patients with metastasis, dabrafenib combined with trametinib or encorafenib with binimetinib can be considered as the preferred first-line treatment option based on phase II trials, in which dabrafenib with trametinib acquired a median OS of 18.2 months as a first-line therapy (36 patients) and 17.3 months as second-line therapy (57 patients),^317^ in parallel encorafenib and binimetinib caused median PFS of no reach and 9.3 months in first-line therapy and second-line therapy setting, respectively.^318^ For patients who are intolerant to dabrafenib combined with trametinib, single-agent dabrafenib or vemurafenib may be considered as a treatment option.^319^ Retrospective studies suggest that patients with advanced NSCLC with BRAF mutations might also benefit from PD-1/PD-L1 inhibitor therapies.^300^ Consequently, immunotherapy regimens based on ICIs, in combination with chemotherapy, can be considered as first-line treatments, particularly for patients with a low disease burden or high PD-L1 levels, and can also represent viable subsequent treatment options for patients whose disease progresses after first-line therapy with BRAF inhibitors.

#### Targeting NTRK1/2/3 fusions

For NTRK1/2/3 fusion NSCLC patients, the current preferred first-line therapy is either entrectinib or larotrectinib,^320^ howbeit the recommendation of larotrectinib is grounded in data from all solid malignancies. In several phase I and II trials, entrectinib demonstrated an overall response rate ranging from 64.5% to 75% for patients with metastatic NSCLC harboring NTRK gene fusions (including but not limited to the STARTRK-2 phase II trial, the STARTRK-1 phase I trial, and the ALKA-372-001 phase I trial), with a slightly lower intracranial objective response rate around 60%,^321^ along with median PFS and OS of 40.7 months and 35.4 months respectively. Other systemic therapies, such as chemotherapy alone or in combination with immunotherapy, can also serve as first-line treatment options, or as subsequent treatments if larotrectinib or entrectinib is inaccessible as the initial therapy. However, relying solely on ICIs may yield poorer outcomes.^300^

#### Targeting MET exon 14 skipping mutations

For patients with advanced or metastatic NSCLC featuring MET exon 14 skipping mutation, capmatinib and tepotinib are currently the preferred first-line treatments, whereas crizotinib is considered useful in specific cases.^322^ Based on the phase II GEOMETRY study, capmatinib as a first-line treatment resulted in a median PFS of 12.4 months,^57^ and in the phase II VISION study,^323^ tepotinib as a first-line treatment led to a median DOR of 46.6 months for previously untreated patients, though this duration reduced to 12.6 months for those who had previously received treatment. Similarly, the NMPA has authorized savolitinib,^324^ gumarontinib^325^ and vebreltinib^326^ for treating metastatic NSCLC with MET exon 14 skipping, based on the promising improvements in ORR and PFS, albeit with enrollment numbers being less than 90 for each study. Moreover, other systemic treatment options (either chemotherapy alone or combined with immunotherapy) are also recommended for first-line treatment and can be used alternatively with capmatinib, tepotinib, or crizotinib for subsequent treatments,^300^ nevertheless the efficacy of monotherapy with ICIs might be lower. In contrast, for metastatic NSCLC with MET amplification, where no FDA-approved targeted therapies are available, immunotherapy-based approaches may be more appropriate.

#### Targeting RET rearrangements

Advanced or metastatic NSCLC patients carrying RET rearrangement have selpercatinib or pralsetinib as the preferred first-line treatment options, with cabozantinib serving as an alternative in specific circumstances for initial therapy.^327^

The evidence is grounded in the phase I/II Libretto-001 study,^328^ which demonstrated the excellent clinical activity of selpercatinib, with a median PFS of 24.9 months. Further support comes from the phase III Libretto-431 trial,^329^ where the median PFS was significantly longer in the selpercatinib group compared to the control group (24.8 months vs. 11.2 months, *P* < 0.001), showcasing a higher rate of intracranial response among patients in the selpercatinib group (82% vs. 58%).^330^ Similarly, in the phaseI/II ARROW study, the overall response rate from pralsetinib treatment was 70% as first-line (19 out of 27) and 61% as second-line (53 out of 87), and the updated data corroborate this efficacy.^331^ Other systemic therapies such as immunotherapies or immunochemotherapies, serving as first-line treatment choices, are appropriate for patients with metastatic RET-positive conditions, and thereby they may be alternated with selpercatinib, pralsetinib or cabozantinib as subsequent treatment options.^300,332^

#### Targeting HER-2 mutations

For patients with advanced or metastatic NSCLC harboring HER-2 mutation, chemotherapy should be employed, either in combination or without immunotherapy,^300,332^ however, single-agent ICIs are generally not recommended. Trastuzumab deruxtecan delivered at lower doses, is advised as a preferred second-line treatment option based on the results of the phase II DESTINY-Lung01 study, with a median OS of 17.8 months.^333^ Other data indicate that T-DM1 is also effective for patients with HER-2 mutation in metastatic or recurrent NSCLC, which is an active area of research at present.

### Frontline immunotherapy

**Fig. 7:**
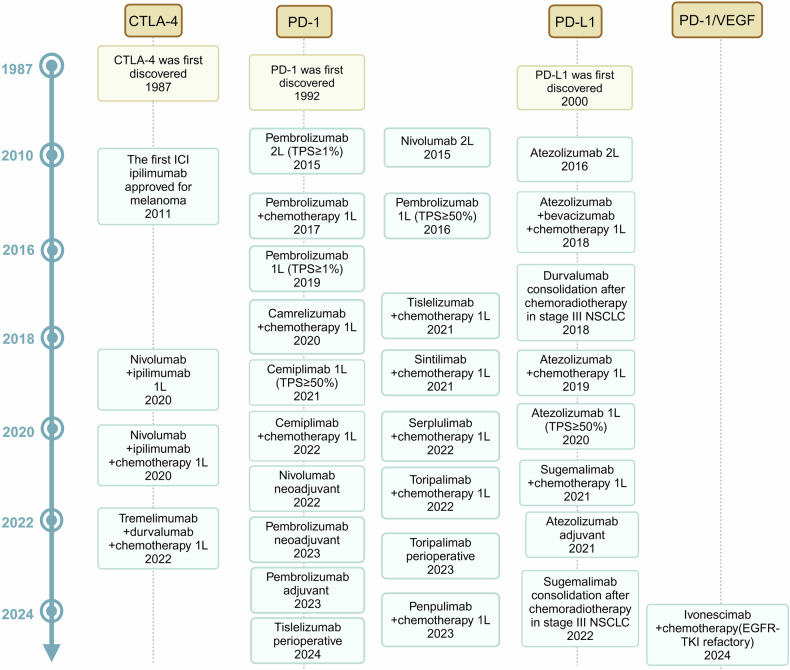
Timeline of the research history and milestone events in immunotherapy for NSCLC. The annotation times refer to the initial FDA or NMPA approval dates, whether under accelerated or regular approval. Many drugs are approved by regional regulatory agencies, and thus their availability may be limited. The illustration shows that immunotherapy has rapidly evolved from a later-line to a front-line treatment for advanced NSCLC and has expanded into both neoadjuvant and adjuvant settings, compared to targeted therapies

In the span of over a decade, the treatment strategy for stage IV NSCLC without specific driver mutations has witnessed substantial transformation, primarily centered around the various applications of immunotherapies (Fig. 7). Currently, in clinical practice, while the choice of immunotherapy for patients does consider PD-L1 expression for single-agent therapies, which is supported by various guidelines, this factor is not typically regarded as a decision-making criterion in the context of combination therapies, despite the level of PD-L1 expression potentially correlating with response depth.^334^ For a more structured review, we delve into the various modalities of combining different drugs with ICIs, but in the realm of clinical practice, the selection of these treatment protocols can be dictated by healthcare providers based on the oncological and biologic characteristics of the tumor and the patient, along with economic considerations.

#### Single-agent immunotherapy

For patients with metastatic NSCLC who harbor no operable driver mutations, pembrolizumab (TPS ≥ 1%),^335^ atezolizumab (TPS ≥ 50%),^336^ or cemiplimab (TPS ≥ 50%)^337^ are recommended, based on three open-label phase III randomized trials – KEYNOTE-042, IMpower-110, or EMPOWER-Lung 1 respectively – which collectively demonstrate significant improvements in PFS and/or OS when compared to chemotherapy (Table 3). The KEYNOTE-024 and EMPOWER-Lung 1 trials permitted a crossover from chemotherapy to ICI therapy, whereas this was not the case for the IMpower110 trial. Overall, these three studies might not adequately represent the squamous cell histology subgroup, which constitutes 18.3%, 43.2% and 24.3% of the total number of patients across KEYNOTE-024,^338^ EMPOWER-Lung 1, and IMpower-110, respectively. In the follow-up, the KEYNOTE-042 trial^339^ demonstrated a significantly longer OS with pembrolizumab monotherapy in the first-line setting compared to chemotherapy across all three PD-L1 TPS groups (≥50%, ≥20%, and ≥1%), recording 20.0 vs. 12.2 months, 17.7 vs. 13.0 months, and 16.7 vs. 12.1 months, respectively. Notably, the five-year OS rate with pembrolizumab was 16.6%, markedly higher than the 8.5% observed with the chemotherapy.^340^

**Table 3:** Frontline immunotherapies for advanced-stage NSCLC

Therapeutic settings	Trial names	Assessed regimes	Trial interventions	PD-L1 status	Primary endpoints	Regulatory status
Single-agent immunotherapy	KEYNOTE-024 Phase III (NCT02142738)^335^	Pembrolizumab	Pembrolizumab vs. Chemotherapy	PD-L1 TPS ≥ 50%	PFS: 10.3 months vs. 6.0 months	FDA-approved/2016
	IMPOWER110 Phase III (NCT02409342)^336^	Atezolizumab	Atezolizumab vs. Chemotherapy	PD-L1 TPS or IC ≥ 1%	OS: 17.5 months vs. 14.1 months (PD-L1 ≥ 1%); 20.2 months vs. 13.1 months (PD-L1 ≥ 50%)	FDA-approved/2020 (only PD-L1 ≥ 50%)
	EMPOWER-Lung 1 Phase III (NCT03088540)^437^	Cemiplimab	Cemiplimab vs. Chemotherapy	PD-L1 TPS ≥ 50%	OS: 26.1 months vs. 13.3 months PFS: 8.1 months vs. 5.3 months	FDA-approved/2021
	KEYNOTE-042 Phase III (NCT02220894)^339^	Pembrolizumab	Pembrolizumab vs. Chemotherapy	PD-L1 TPS ≥ 1%	OS: 16.7 months vs. 12.1 months	FDA-approved/2019
Immunotherapy in combination with chemotherapy	KEYNOTE-021 Phase II (NCT02039674)^846^	Pembrolizumab + Chemotherapy (Non-squamous)	Pembrolizumab + Chemotherapy vs. Chemotherapy	Regardless of PD-L1 levels	ORR: 55% vs. 29% PFS: 13.0 months vs. 8.9 months	FDA-accelerated approval/2017
	KEYNOTE-189 Phase III (NCT02578680)^847,848^	Pembrolizumab + Chemotherapy (Non-squamous)	Pembrolizumab + Chemotherapy vs. Chemotherapy	Regardless of PD-L1 levels	OS: 22.0 months vs. 10.7 months PFS: 9.0 months vs. 4.9 months	FDA- traditional approval /2018
	KEYNOTE-407 Phase III (NCT02775435)^849^	Pembrolizumab + Chemotherapy (Squamous)	Pembrolizumab + Chemotherapy vs. Chemotherapy	Regardless of PD-L1 levels	OS: 15.9 months vs. 11.3 months PFS: 6.4 months vs. 4.8 months	FDA-approved/2018
	IMPOWER130 Phase III (NCT02367781)^343^	Atezolizumab + Chemotherapy (Non-squamous)	Atezolizumab + Chemotherapy vs. Chemotherapy	Regardless of PD-L1 levels	OS: 18.6 months vs. 13.9 months PFS: 7.0 months vs. 5.5 months	FDA-approved/2019
	IMPOWER131 Phase III (NCT02367794)^850^	Atezolizumab + Chemotherapy (Squamous)	Atezolizumab + Chemotherapy vs. Chemotherapy	Regardless of PD-L1 levels	OS: 14.2 months vs. 13.5 months PFS: 6.3 months vs. 5.6 months	
	EMPOWER-Lung 3 Phase III (NCT03409614)^851^	Cemiplimab + Chemotherapy	Cemiplimab + Chemotherapy vs. Chemotherapy	Regardless of PD-L1 levels	OS: 21.9 months vs. 13.0 months PFS: 8.2 months vs. 5.0 months	FDA-approved/2022
	ORIENT-11 Phase III (NCT03607539)^852^	Sintilimab + Chemotherapy (Non-squamous)	Sintilimab + Chemotherapy vs. Chemotherapy	Regardless of PD-L1 levels	PFS: 8.9 months vs. 5.0 months	NMPA-approved/2021
	ORIENT-12 Phase III (NCT03629925)^853^	Sintilimab + Chemotherapy (Squamous)	Sintilimab + Chemotherapy vs. Chemotherapy	Regardless of PD-L1 levels	PFS: 5.1 months vs. 4.9 months	NMPA-approved/2021
	CAMEL Phase III (NCT03134872)^854^	Camrelizuab + Chemotherapy (Non-squamous)	Camrelizuab + Chemotherapy vs. Chemotherapy	Regardless of PD-L1 levels	PFS: 11.3 months vs. 8.3 months	NMPA-approved/2020
	CAMEL-sq Phase III (NCT03668496)^855^	Camrelizuab + Chemotherapy (Squamous)	Camrelizuab + Chemotherapy vs. Chemotherapy	Regardless of PD-L1 levels	PFS: 8.5 months vs. 4.9 months	NMPA-approved/2021
	CHOICE-01 Phase III (NCT03856411)^352^	Toripalimab + Chemotherapy	Toripalimab + Chemotherapy vs. Chemotherapy	Regardless of PD-L1 levels	PFS: 8.4 months vs. 5.6 months	NMPA-approved/2022
	RATIONALE 304 Phase III (NCT03663205)^856^	Tislelizumab + Chemotherapy (Non-squamous)	Tislelizumab + Chemotherapy vs. Chemotherapy	Regardless of PD-L1 levels	PFS: 9.7 months vs. 7.6 months	NMPA-approved/2021
	RATIONALE 307 Phase III (NCT03594747)^348^	Tislelizumab + Chemotherapy (Squamous)	Tislelizumab + Chemotherapy vs. Chemotherapy	Regardless of PD-L1 levels	PFS: 7.6 months vs. 5.5 months	NMPA-approved/2021
	ASTRUM-004 Phase III (NCT04033354)^351^	Serplulimab + Chemotherapy (Squamous)	Serplulimab + Chemotherapy vs. Chemotherapy	Regardless of PD-L1 levels	PFS: 8.3 months vs. 5.7 months	NMPA-approved/2022
	AK105-302 Phase III (NCT03866993)^353^	Penpulimab + Chemotherapy (Squamous)	Penpulimab + Chemotherapy vs. Chemotherapy	Regardless of PD-L1 levels	PFS: 7.6 months vs. 4.2 months	NMPA-approved/2023
	GEMSTONE-302 Phase III (NCT03789604)^857,858^	Sugemalimab + Chemotherapy	Sugemalimab + Chemotherapy vs. Chemotherapy	Regardless of PD-L1 levels	PFS: 7.8 months vs. 4.9 months OS: 25.4 months vs. 16.9 months	NMPA-approved/2021
Immunotherapy with dual ICIs	CHECKMATE-227 Phase III (NCT02477826)^859^	Nivolumab + Ipilimumab	Nivolumab + Ipilimumab vs. Chemotherapy	PD-L1 ≥ 1%	OS: 17.1 months vs. 14.9 months	FDA-approved/2020
	NEPTUNE Phase III (NCT02542293)^359^	Durvalumab + Tremelimumab	Durvalumab + Tremelimumab vs. Chemotherapy	Regardless of PD-L1 levels	OS: 11.7 months vs. 9.1 months (TMB ≥ 20 mut/Mb); 10.9 months vs. 12.1 months (ITT population)	
	MYSTIC Phase III (NCT02453282)^358^	Durvalumab + Tremelimumab	Durvalumab + Tremelimumab vs. Chemotherapy	PD-L1 ≥ 25%	OS: 11.9 months vs. 12.9 months PFS: 3.9 months vs. 5.4 months	
Enhanced combination therapy (four-drug regimen)	IMPOWER150 Phase III (NCT02366143)^364^	Atezolizumab + Carboplatin + Paclitaxel + Bevacizumab (Non-squamous)	Atezolizumab + Carboplatin + Paclitaxel + Bevacizumab (ABCP) vs. Atezolizumab + Carboplatin + Paclitaxel (ACP) vs. Bevacizumab + Carboplatin + Paclitaxel (BCP)	Regardless of PD-L1 levels	OS: 19.2 months (ABCP) vs. 14.7 months (BCP) PFS: 8.3 months (ABCP) vs. 6.8 months (BCP)	FDA-approved/2018
	ORIENT-31 Phase III (NCT03802240)^424^	Sintilimab + IBI305 (bevacizumab biosimilar) + Chemotherapy (EGFR-TKI failure)	Sintilimab + IBI305 + Chemotherapy vs. Sintilimab + Chemotherapy vs. Chemotherapy	Regardless of PD-L1 levels	PFS: 7.2 months vs. 5.5 months vs. 4.3 months	NMPA-approved/2023 (EGFR-TKI failure)
	CHECKMATE 9LA Phase III (NCT03215706)^860^	Nivolumab + Ipilimumab + Chemotherapy	Nivolumab + Ipilimumab + two cycles of Chemotherapy vs. Chemotherapy	Regardless of PD-L1 levels	OS: 14.1 months vs. 10.7 months PFS: 6.8 months vs. 5.0 months	FDA-approved/2020
	POSEIDON Phase III (NCT03164616)^354^	Durvalumab + Tremelimumab + Chemotherapy	Durvalumab + Tremelimumab + Chemotherapy vs. Chemotherapy	Regardless of PD-L1 levels	OS: 14.0 months vs. 11.7 months PFS: 6.2 months vs. 4.8 months	FDA-approved/2022
	CCTG BR34 Phase II (NCT03057106)^861^	Durvalumab + Tremelimumab + Chemotherapy	Durvalumab + Tremelimumab + Chemotherapy vs. Durvalumab + Tremelimumab	Regardless of PD-L1 levels	OS: 16.6 months vs. 14.1 months PFS: 7.7 months vs. 3.2 months	

Refer to Tables 1 and 2 for abbreviations

#### Immunotherapy in combination with chemotherapy

While monotherapy with ICIs has shown superior efficacy in tumors with high PD-L1 expression (>50%) as compared to platinum-based doublet chemotherapy, the number of patients with such advantage status remains limited. Furthermore, chemotherapy not only eliminates the targets of T cells, the tumor cells, leading to immunogenic cell death of these cancer cells, but also results in the depletion of myeloid-derived suppressor cells (MDSCs), tumor-associated neutrophils (TANs), macrophages, and T_reg_s, as well as the inhibition of angiogenesis. Consequently, to enhance the efficacy of ICIs, multiple studies have evaluated their combination with chemotherapy, including NSCLC patients with low PD-L1 expression.

In several phase III trials – the KEYNOTE-189 trial^341^ (pembrolizumab plus chemotherapy in non-squamous NSCLC), the KEYNOTE-407 trial^342^ (pembrolizumab plus chemotherapy in squamous NSCLC), the IMpower-130 trial^343^ (atezolizumab plus chemotherapy in non-squamous NSCLC),^344^ and the ORIENT-11 trial^345^ (sintilimab plus chemotherapy in non-squamous NSCLC) (Table 3), the patients showed improved OS compared to chemotherapy alone. However, comparing the outcomes across these studies is challenging due to substantial variations in follow-up times. In the past four years, several additional ICIs such as cemiplimab,^346,347^ sugemalimab, tislelizumab,^348^ camrelizumab,^349,350^ serplulimab,^351^ and toripalimab^352^ have also been approved for use in combination with chemotherapy by various regulatory agencies due to their proven ability to improve survival, while others are still awaiting approval.^353^ However, in the durvalumab+ chemotherapy subgroup of POSEIDON trial^354^ and the nivolumab+ chemotherapy arm of CHECKMATE-227 Part 2 trial,^355^ neither approach resulted in improved OS compared to chemotherapy alone, likely because a large number of patients in the control group also received immunotherapy after their disease progressed.

#### Dual-ICI immunotherapy

As early as 2005, in multiple mouse tumor models, researchers discovered that simultaneously blocking the independent redundant pathways mediated by anti-CTLA-4 and anti-PD1 could induce and/or expand the repertoire of tumor-reactive T cell epitopes. Furthermore, the presence of CTLA-4/PD-1 double-positive T cells indicates a deeply exhausted phenotype in human tumors, suggesting that dual PD1/CTLA4 blockade may exhibit more immunostimulatory activity.^356^ Indeed, in 2015, this immunotherapeutic regimen was approved for the treatment of melanoma. Similarly, CHECKMATE-227 trial^357^ confirmed that, for stageIV or recurrent NSCLC nivolumab plus ipilimumab (an antibody blocking CTLA-4) as first-line treatment significantly improves the five-year OS rate compared to chemotherapy (for PD-L1 ≥ 1%, 24% vs. 14%; and for PD-L1 < 1%, 19% vs. 7%, respectively). However, nivolumab plus ipilimumab did not receive FDA approval for patients with PD-L1 TPS < 1%. Recently, specific co-mutations, such as KRAS, STK11, and KEAP1 mutations, have also been found to be suitable for this dual ICI (or combined chemotherapy) treatment. Conversely, the phase III MYSTIC trial^358^ and the NEPTUNE trial^359^ failed to demonstrate that, as first-line treatment of NSCLC, combination therapy with durvalumab or tremelimumab (an antibody blocking CTLA-4) could improve OS or PFS over standard chemotherapy.

#### Enhanced combination therapy (Four-drug regimen)

Given the significance of chemotherapy in NSCLC, there are reports of trials involving chemotherapy in conjunction with dual immunotherapy. In the CHECKMATE-9LA study,^360^ for patients with stageIV or recurrent NSCLC, with a total survival follow-up of at least 47.9 months, the combination of nivolumab plus ipilimumab with short-course chemotherapy (two cycles) significantly prolonged OS for all randomized participants, compared to single-agent chemotherapy (5-year OS rate: 18% versus 11%).^361^ This treatment strategy shows impressive significance as patient cohorts with PD-L1 expression below 1% or particularly those with squamous histology, have critically unmet medical needs.^362^ Similarly, in the POSEIDON study,^354^ when compared to chemotherapy alone, the combination of tremelimumab + durvalumab + chemotherapy significantly extended PFS (6.2 months vs. 4.8 months) and OS (14.0 months vs. 11.7 months; 5-year survival rate, 15.7% vs. 6.8%), including the PD-L1 negative subgroup, although the benefit appeared to be more moderate in patients with squamous histology.^363^ Likewise, in the IMpower-150 study for metastatic non-squamous wild-type NSCLC patients, regardless of PD-L1 expression levels, the median PFS and OS in the ABCP (atezolizumab combined with BCP) arm were longer than in the BCP (bevacizumab combined with carboplatin and paclitaxel) arm (8.3 months vs. 6.8 months, *P* < 0.001; and 19.2 months vs. 14.7 months, *P* = 0.02).^364^

However, not all studies universally endorse that the intensified regimens of four-drug therapies are perceived as more efficacious than treatments involving two or three drugs, exemplified as outcomes from the Japan registry trial,^365^ the ONO-4538-52/TASUKI-52 trial and CCTG BR34 trial.

Nowadays, the available data highlight at least five frontline immunotherapy options for NSCLC in real-world practice, thereby clinicians must carefully weigh out the tumor-specific attributes like PD-L1 expression, as well as treatment cost, and safety concerns, given the absence of superior biomarkers and survival data to guide their selection among these therapies. Typically, different levels of combination therapy remain the primary option, especially when considering factors like high tumor burden, immediate symptom relief, specific oncogene mutations, and good chemotherapy tolerance, as well as a slightly better OS improvement, although almost all guidelines and consensus proposing single immunotherapy for patients with PD-L1 TPS above 50%.^335,339^

Nevertheless, regarding how to conduct combination therapy, we believe there are several overarching considerations that need to be clarified. Primarily, the selection of drugs, the dosages of chemotherapy drugs, the sequence of administration, and the schedule of treatment in current regimens might not be optimal. The development of chemotherapy has been anchored on the concept of the maximum tolerated dose (MTD), therefore a suppressive effect of immunotherapies is an inevitable concern.^366^ As a consequence, at least in certain scenarios particularly for patients who are immunocompromised, treatments involving low-dose dual-drug chemotherapy, standard-dose single-agent chemotherapy, or the use of intermittent treatment cycles with holidays,^170^ combined with immunotherapy, might prove to be more efficacious. As proposed in the CHECKMATE-9LA trial^362^ and studies evaluating sintilimab,^367^short-course chemotherapy can be sufficient when combined with immunotherapy, nevertheless, this requires confirmation through clinical trials. Lastly, in general, exceeding simultaneously four-drug therapy is not advisable yet, because most combination strategies do not exhibit the so-called synergistic effects, and even additive effects are less likely to occur as well.

### Chemotherapy

Ideally, for patients with metastatic NSCLC, efforts should be directed towards targeted therapy or immunotherapy, however, there are instances where patients may not qualify for targeted or immunotherapies due to the absence of driver mutations or the unavailability of drugs, along with contraindications to PD-1 or PD-L1 inhibitors. In such cases, platinum-based combination regimens are typically employed, which have been shown to yield a survival rate of 30% to 40% after one year, often outperforming monotherapies.^368,369^ For non-squamous NSCLC, bevacizumab in combination with chemotherapy is also an option, based on the findings from the phase II/III ECOG 4599 trial^370^ which showed a significant improvement in median OS compared to chemotherapy alone (12.3 months vs. 10.3 months, *P* = 0.003). Similarly, the POINTBREAK trial^371^ suggested that chemotherapy combined with bevacizumab is a reasonable option, although patients over the age of 75 did not benefit. However, the AVAil study, a phase III randomized trial did not find that adding bevacizumab to cisplatin plus gemcitabine might increase survival rates.^372^ Based on clinical data and FDA approval, biosimilar versions of bevacizumab can be utilized in systemic treatment regimens for metastatic NSCLC.^373^ Previously, patients with brain metastases were excluded from bevacizumab treatment due to concerns about cerebral hemorrhage, but data indicate that bevacizumab can be used in patients with controlled brain metastases.^374^ Additionally, for patients with metastatic squamous cell lung cancer, the SQUIRE trial demonstrated that incorporating necitumumab into chemotherapy extended median OS by 1.6 months (with a hazard ratio of 0.84), however, the addition of necitumumab might not be advantageous due to the increased toxicity and costs involved.^375^ Moreover, for patients with poor performance status (PS ≥ 2) and elderly patients with advanced NSCLC, using non-platinum regimens such as gemcitabine plus docetaxel or gemcitabine plus vinorelbine, or treating with single-agent chemotherapy,^376^ is a reasonable approach. However, a phase III randomized trial focusing on elderly (70-89 years) patients with advanced NSCLC demonstrated that weekly paclitaxel, combined with monthly carboplatin, resulted in improved survival (10.3 months vs. 6.2 months), compared to gemcitabine or vinorelbine monotherapy.^377^

### Immunotherapy in special populations

In fact, driver gene-positive NSCLC exhibits various the biodiversity characteristics – displaying heterogeneous sensitivity to cancer immunotherapy, however, regrettably, clinical data on the efficacy of ICIs in this population is currently lacking, and even if any data available, they also would stem from subgroup analyses of retrospective studies.^378^ The scarcity of prospective data is mainly attributed to the systematic exclusion of NSCLC driven by oncogenes like EGFR, ALK or ROS1 from clinical trials, and/or the lack of standardized comprehensive genomic analyses in most studies.^379^ Consequently, for genetic alterations beyond EGFR mutations, ALK and ROS1 fusions, if drugs, such as KRAS^G12C^ inhibitor,^380^ might be unavailable, conventional first-line treatment comprising chemoimmunotherapy or with bevacizumab is also a viable option. Moreover, considering the genetic mutation status is not necessary when making treatment decisions in a second-line setting.

Secondly, patients with multiple chronic disease states (MCC) or autoimmune disorders,^381^ who exhibit poorer PS (2-3) or brain metastases, or are elderly at 70 years or older,^382^ face a lack of prospective data to optimally guide their treatment for advanced cancer,^383^ primarily due to their routine exclusion from or insufficient enrollment into clinical trials.^383^ However, the results from the phase III IPSOS study^384^ evaluating atezolizumab, the SAKK 19/17 study^385^ evaluating durvalumab, and the CHECKMATE-817 study^386^ evaluating nivolumab and ipilimumab, along with the finding from a meta-analysis, all indicate clinical survival benefits for this patient population, despite potential differences in disease conditions.^387^ Notably, immunotherapy remains contraindicated for patients who have undergone organ transplantation.

Another important consideration is regarding re-challenge of immunotherapy in patients following recovery from toxicity. There is substantial evidence indicating that selected patients may benefit from a re-challenge with ICIs after recovery,^388^ though generally, for grade 4 toxicities, immunotherapy is advised to be permanently discontinued unless the toxicity appears in endocrine organs which are amenable to treatment with hormone replacement. In all cases, decisions should be personalized and supported by multidisciplinary teams including experts from medical fields beyond oncology, ideally through switching to alternative ICIs or using immunosuppressants to prevent toxicity, as well as early identification of AEs.^388^

### Maintenance therapy after first-line systemic treatment

**Fig. 8:**
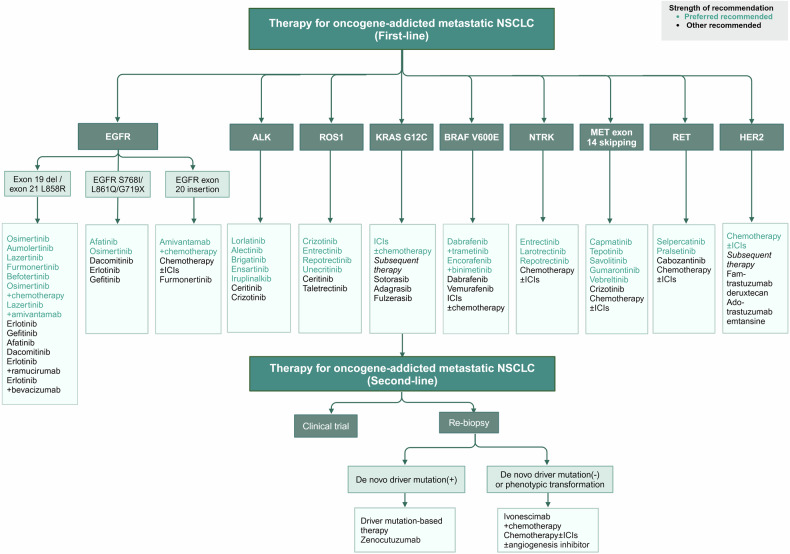
Recommended therapy algorithm for oncogene addicted metastatic NSCLC. The flowchart provides a comprehensive view of clinical decision-making for targeted therapies in metastatic NSCLC. It highlights the importance of flexibility in treatment decisions, such as choosing between combining osimertinib with chemotherapy or using osimertinib as a single agent, based on tumor burden and patient preferences. For EGFR and ALK mutations, third-generation TKIs are generally the preferred choice. The decision to combine ICIs with chemotherapy should consider the specific characteristics of the tumor per se, similar to non-mutated tumors (as shown in Fig. 9), although the efficacy may be compromised

During the era of chemotherapy, patients with metastatic NSCLC received four cycles of initial systemic chemotherapy, followed by maintenance therapy. The data from the PARAMOUNT trial suggested that four cycles of platinum-based therapy might not be optimal, and in fact, tumors could continuously shrink after 4 to 6 cycles of chemotherapy. A meta-analysis suggested that continuing the initial regimen beyond 4 to 6 cycles might increase PFS, but at the cost of more side effects. While a phase III randomized trial posited that continuing chemotherapy beyond 4 to 6 cycles offers no benefit,^389^ this might be due to the premature termination of treatment for neurotoxicity associated with taxane-based chemotherapy, therefore, arguments against prolonging first-line chemotherapy beyond 6 cycles may be limited to taxane-based regimens^390^ (Fig. 8).

For patients undergoing immunotherapy (alone or in conjunction with chemotherapy), where the first-line fixed-cycle treatment leads to either partial or complete response (PR or CR), or even stabilization, continuation of immunotherapy alone, or combined with single-agent chemotherapy, olaparib or anti-angiogenic targeted therapy can serve as a maintenance treatment to further prolong PFS or control the diseases effectively, despite lacking high-quality evidence.^371,391,392^ Notably, prior to the introduction of immunotherapy, sustained anti-angiogenic treatments could be continued until disease progression or the onset of unmanageable side effects. In comparison, for immunotherapy, the consensus suggests a minimum effective treatment period of two years, as evidenced by studies such as KEYNOTE-010, KEYNOTE-024, KEYNOTE-042, KEYNOTE-189, and CHECKMATE-227, however, the IMpower-110, IMpower-130, and IMpower-150 trials^393^ endorse the practice of maintaining immunotherapy without a set termination date. Therefore, at present, given this clear inconsistency, it is necessary to have full communication with the patient to establish treatment decisions. This also highlights the need for further biomarker development, such as sensitive nucleic acid or protein profiles, to stratify patients. The duration of maintenance therapy with anti-angiogenic agents and immunotherapy, extended up to 2 years or more, hinges on individual patient variables or the strategy of combined therapy.^394^ Nevertheless, the enduring impacts of chronic, albeit mild, toxicities, particularly in the realm of immunotherapy, should not be discounted.

### Later-line therapy

**Fig. 9:**
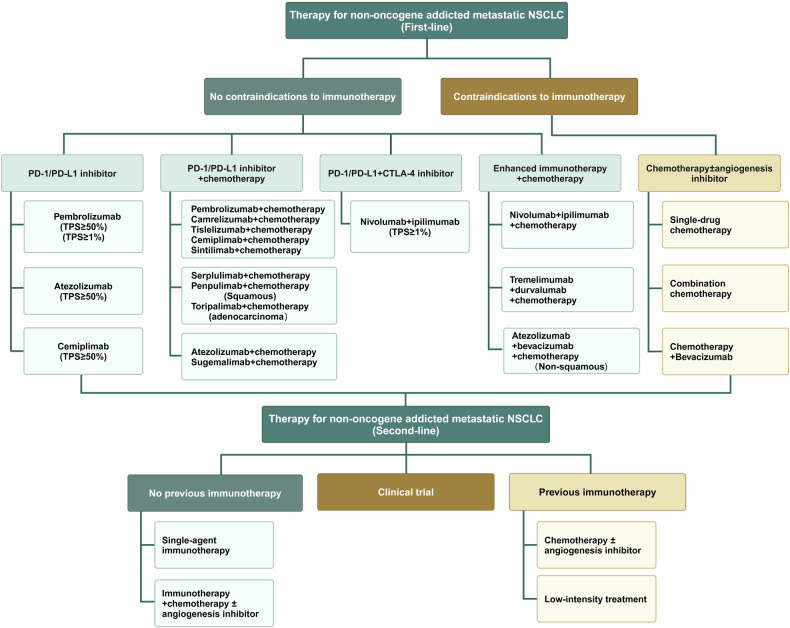
Recommended therapy algorithm for non-oncogene addicted metastatic NSCLC. This flowchart categorizes immunotherapy options primarily based on the characteristics of the drugs and the features of the tumor, which are currently numerous and should be selected based on availability. While standard regimens exist for chemotherapy combined with immunotherapy, special considerations, such as the neurotoxicity associated with nab-paclitaxel, may necessitate alternative treatment strategies. Anti-angiogenic biosimilars can serve as parallel substitutes

For NSCLC patients whose disease progresses during or following first-line therapy, the choice of second-line and subsequent systemic treatments hinges on specific symptoms, genetic mutations, histopathological subtypes, and PS status (Fig. 9).

#### Addressing local progression

In scenarios of indolent and/or asymptomatic disease progression, patients still may continue to receive systemic TKI or ICI therapies if such treatment is deemed to offer clinical benefit. Subsequently, for patients experiencing oligoprogression^395^- characterized by a single, smaller metastasis or a limited number of lesions rather with other aspects effectively controlled – localized treatments such as stereotactic radiosurgery (SRT),^396^ palliative surgery or image-guided thermal ablation are considered feasible options because of offering more precise local control and potential for long-term survival opportunities,^397^ although the efficacy data often originates from studies with small cohort sizes.

#### Addressing systemic progression

For patients experiencing systemic progression with diffuse, widespread diseases, there is a need to alter the approach to systemic therapy, yet which has generally been less than satisfactory, and remains an area of active investigation.^398^ And indeed, in subsequent-line treatments, the key consideration will be the necessity to combine drugs from different pathways to prevent the overlap of resistance mechanisms and toxicity profiles.

#### Later-line treatment in NSCLC with positive driven-mutation

##### Continuing targeted treatment

For metastatic NSCLC patients who already harbored mutations prior to treatment, such as BRAF,^317^ cMET and KRAS^G12C^ ^399^ and HER-2 mutations,^400^ treatment for their respective targets can be provided. For EGFR exon 20 insertion mutations, amivantamab^301^ and sunvozertinib,^401^ have been approved by the FDA and NMPA, respectively, for second-line treatment of locally advanced or metastatic NSCLC, based on the data from the CHRISTYS trial and a series of WU-KONG trials. Additionally, mobocertinib^299^ can also be considered when other drugs are not available.

Performing a re-biopsy for comprehensive genetic testing, and excluding small cell lung cancer transformation (a phenomenon observed in roughly 5% of EGFR TKI-resistant tumors), represents an optimal procedural step.^402^ If a new target arises, such as ALK or EGFR^T790M^ mutation in patients with resistance to EGFR TKIs, one proceeds with the cycle of first-line therapy. When C797S appears in cis, first-generation EGFR TKIs targeting C797S can be combined with third-generation TKIs, although they offer only transient clinical benefits. Likewise, some mutations that confer resistance to lorlatinib in ALK-rearranged NSCLCs, can be re-sensitized to earlier-generation ALK inhibitors, for instance, ALK^C1156Y+L1198F^ mutation to crizotinib, ALK^L1256F^ mutation to alectinib.^403^

In ROS1-mutated NSCLC, with progression occurring after treatment with crizotinib or ceritinib, including cases with CNS progression, entrectinib or repotrectinib are potential alternatives^311^ and lorlatinib is also a viable option.^49^ In EGFR-mutated tumors that have acquired resistance to EGFR TKIs, HER3 expression is typically upregulated. Patritumab deruxtecan, an ADC targeting HER3 with a payload of a topoisomerase I inhibitor, has shown activity as monotherapy.^404^ Lastly, brigatinib, an ALK TKI that also shows activity against ROS1, FLT3 and EGFR in preclinical studies, has been found to overcome resistance to osimertinib in conjunction with cetuximab based on preclinical studies and case reports. For NSCLC patients harboring an NRG1 gene fusion, based on the eNRGy study showing an ORR of 33% and a median DOR of 7.4 months, zenocutuzumab is recommended as a second-line treatment until disease progression or unacceptable toxicity in December 2024.^405^

Certainly, for patients without emerging driver mutations, selecting TKI dose escalation to attempt to increase blood or central nervous system (CNS) drug concentrations has been supported by various small sample reports, such as doubling osimertinib dosage (intracranial PFS = 3.8–7.0 months)^406^ and alectinib dosage (median duration = 7.7 months), which has improved response and treatment duration. Conversely, for patients experiencing disease progression after initial use of lorlatinib, administering lorlatinib at doses exceeding 100 mg daily may fail to offer therapeutic benefits for ALK-rearranged NSCLC patients with CNS diseases, highlighting the complexity of managing CNS progression after third-generation TKI therapy. However, for patients harboring ROS1, RET or other mutations, the efficacy and safety of escalated dosages remain uncertain, and cost-benefit analysis does not justify this approach, at least at present.

For patients with squamous cell carcinoma who progress after chemotherapy, second-line afatinib is also an appropriate option. However, with the emergence of immunotherapy, questions have been raised on whether this appropriateness is still persisting, given that the PFS associated with this treatment is just 2.4 months.^407^

##### Combinations with anti-angiogenic therapy

The joint use of EGFR or ALK TKIs with anti-angiogenic agents has yielded mixed results so far. While early phase II and III trials have shown that adding anti-angiogenic agents to first-line EGFR TKIs in untreated EGFR-mutated NSCLC patients improves PFS, there was no significant improvement in OS. Similarly, in the second-line setting, only two studies comparing osimertinib plus bevacizumab versus single-agent osimertinib failed to demonstrate any significant improvements in PFS or OS,^408^ with these findings supported by meta-analyses. In contrast, in ALK-rearranged NSCLC patients, the addition of anti-angiogenic drugs like alectinib combined with bevacizumab has shown potential benefits, but further confirmation is still required. For multi-target angiogenesis inhibitors, the scenario might differ slightly. Indeed, small-sample studies and a pooled analysis indicate that for NSCLC patients harboring EGFR mutations, the addition of anlotinib following progression on EGFR TKI therapy could safely overcome resistance, though the efficacy might be modest.

##### Combined with chemotherapy

Combination chemotherapy remains a primary strategy for overcoming resistance and achieving survival improvement goals,^261^ although there is no high-level evidence.^409^ Regarding first-generation EGFR-TKIs, unlike data from their first-line treatments which showed that the addition of gefitinib with chemotherapy, compared to gefitinib alone^410^ notably extended PFS and OS, the IMPRESS study^411^ failed to demonstrate any benefit of chemotherapy and continued gefitinib over chemotherapy alone. With the advent of third-generation TKIs, the landscape has shifted, endorsed by the phase III MARIPOSA-2 study in which in EGFR-mutant advanced NSCLC following osimertinib resistance,^412^ amivantamab-chemotherapy and amivantamab-lazertinib-chemotherapy can improve the median intracranial PFS, compared to chemotherapy alone, while no statistically significant difference in OS was reported in the preliminary analysis. Therefore, given this result and extrapolations from the FLAURA2 study,^280^ chemotherapy (at least single-agent) in combination with osimertinib or other TKIs is a suitable choice for patients with resistance and holds potential for extending PFS, if without intolerable toxicity. The ongoing randomized phase III COMPEL trial (NCT04765059) aims to determine, following osimertinib resistance in extracranial disease progression during first-line therapy, whether chemotherapy should be continued concurrently with osimertinib. In ALK-rearranged NSCLC, there is a lack of prospective data on this issue. However, data from a small retrospective study suggest that continuing an ALK TKI with chemotherapy after progression on second-generation ALK TKI might extend PFS. On a separate note, there is a significant question, regarding whether combining an EGFR or ALK TKI with chemotherapy as initial therapy versus sequential TKIs followed by chemotherapy alone (for non-squamous cancers, also combined with anti-angiogenic therapy), has advantages in terms of PFS and/or OS.

##### Chemoimmunotherapy based treatment

Owing to the significant clinical and biological diversity of NSCLC subtypes driven by their genomic profiles, the response to cancer immunotherapies can exhibit variations. For example, genetic variants associated with smoking, such as RAS mutations, are associated with high antitumor immune responses, despite the lack of confirmatory evidence. Consequently, if immunotherapy has not been utilized as first-line treatment, the use of single-agent immunotherapies, including nivolumab,^413^ atezolizumab,^414^ and pembrolizumab,^415^ has demonstrated survival benefits compared to conventional chemotherapies^416^ such as docetaxel. Moreover, the concurrent administration of anti-vascular targeted therapies is feasible and supported by evidence from studies on hepatocellular carcinoma and renal cell carcinoma. Nevertheless, given the data from the CAURAL trial^417^ and CHECKMATE-370,^418^ which do not advocate for concurrent or short-term sequential ICIs with specific EGFR or ALK TKIs,^419^ the use of such combinations outside well-designed clinical trials is not recommended. Of course, for NSCLC that undergoes a conversion to small cells, immunotherapy in conjunction with chemotherapy is an option, although its efficacy falls short as compared to that for primary SCLC.

In order to further enhance efficacy, combining ICIs with chemotherapy and/or anti-angiogenic treatments^420^ have been explored, leading to notable advancements albeit with an unevenly progressed trajectory. In the phase III ATTLAS trial, for patients with NSCLC harboring EGFR mutations or ALK rearrangements, the combination of atezolizumab and bevacizumab with chemotherapy (ABCP) showed significantly higher objective response rates (69.5% vs. 41.9%, *P* < 0.001) and median PFS (8.48 vs 5.62 months, *P* = 0.004) compared to the chemotherapy (PC) arm. The benefit in PFS was more pronounced with increasing levels of PD-L1 expression, with hazard ratios of 0.47, 0.41, and 0.24 for PD-L1 ≥ 1%, ≥10%, and ≥50%, respectively. However, OS was similar between the ABCP and PC arms (20.63 vs. 20.27 months, *P* = 0.975).^421^ Likewise, the updated final exploratory analysis of the IMpower-150 trial revealed no significant difference in OS between all EGFR mutation-positive NSCLC patients treated with ABCP versus BCP (chemotherapy plus bevacizumab). Consequently, the IMpower-150 regimen did not receive approval from the FDA as a subsequent treatment following EGFR-TKI therapy.^422^ Similarly, CHECKMATE-722^423^ found that nivolumab plus chemotherapy compared to chemotherapy alone could not prolong PFS, akin to the outcomes observed in IMpower-151.^422^ The ORIENT-31 trial,^424^ evaluated a quadruple regimen comprising sintilimab, bevacizumab biosimilar, and chemotherapy, for treating NSCLC patients with EGFR mutations and preliminary data suggested a significant extension of median PFS rather than OS in the four drug group, compared to chemotherapy alone (6.9 months versus 4.3 months, *P* < 0.0001). Likewise, in patients with advanced NSCLC who had EGFR TKI treatment failure, toripalimab,^425^ ivonescimab^426^ (approved by the NMPA), sintilimab (approved by the NMPA) or tislelizumab^427^ combined with chemotherapy compared to chemotherapy has shown improvements in PFS of no more than 3 months, recently which was also endorsed by a meta-analysis.^420^ On the contrary, the phase III KEYNOTE-789 trial^428^ failed to demonstrate that the addition of pembrolizumab to chemotherapy in patients with TKI-resistant, EGFR-mutant, metastatic nonsquamous NSCLC can significantly prolong PFS or OS compared to chemotherapy alone.^429^

Similarly, there is potential for response to the PD-L1/PD-1 axis blockade in NSCLC patients with KRAS mutations, as evidenced in preliminary findings from the phase III CHECKMATE-057 trial where single-agent anti-PD-1 antibody treatment showed a significant improvement in OS, compared to docetaxel for the KRAS-mutated NSCLC subgroup.^380^ In addition, subgroup analyses from the IMpower-150 trial,^430^ suggested that the combination of chemotherapy and immunotherapy could benefit KRAS-mutated NSCLC patients, and notably, patients with KRAS, TP53, STK11, and/or KEAP1 co-mutations experienced the least benefit compared to those with KRAS mutation alone.^431^ Recently, chemotherapy combining PD-L1 and CTLA-4 dual inhibition has shown efficacy in mitigating resistance to PD-(L)1 inhibition in NSCLC patients with STK11 and/or KEAP1 alterations.^113^ Whether PD-L1/PD-1 axis blocking immunotherapies can be simultaneously combined with KRAS-mutated TKIs, such as sotorasib, for KRAS-mutated NSCLC subtypes remains unknown. Similarly, for NSCLC patients with BRAF^V600^ mutations, there have been reports of sustained benefits from immunotherapy, but whether their efficacy is enhanced when used in combination with BRAF and/or MEK inhibitors (including FDA-approved drugs vemurafenib, dabrafenib, and trametinib), similar to that against melanoma, might also require real-world data, as controlled studies may not be feasible. Moreover, for NSCLC patients harboring other driver gene mutations such as ROS1, NTRK and RET fusions, FGFR, HER-2, and METex14 mutation or overexpression, immunotherapy in combination with chemotherapy± bevacizumab may be considered an effective and tolerable second-line treatment option, regardless of PD-L1 expression levels,^431,432^ although concrete evidence is lacking.

Note that, in the era of immunotherapy, if the goal is merely to enhance the efficacy of immunotherapy, dose-dependent chemotherapy drugs or ADCs might be suitably utilized at reduced doses or as monotherapy,^433^ especially in patients with compromised liver, kidney or bone marrow function,^434^ being that high-dose chemotherapy can significantly impair the effector capabilities of T cells.

#### Later-line treatment in NSCLC with negative driven-mutation

Given that most NSCLC patients lack actionable driver mutations and have often undergone chemotherapy± immunotherapy, second-line treatment options and efficacy are typically limited, with specific recommendations hinging on previous treatments, tumor histology, and patient-specific factors including overall health, comorbidities, organ function, and preferences.

The initial step involves molecular testing of the patient to identify new therapeutic targets and phenotypic changes, enabling informed decisions on whether to add chemotherapy or switch to targeted therapy, or enroll a clinical trial. Of course, integrated comprehensive supportive and palliative care,^435^ are key components in managing patients with advanced NSCLC. In conclusion, the primary emphasis should be placed on sidestepping medications with overlapping mechanisms of action, meticulously tracking adverse effects, instituting concurrent localized treatments, and, in the long term, pioneering innovative therapeutic approaches.

##### Chemotherapy-based combination therapy

Firstly, if patients have progressed on first-line platinum-based combination therapy and have not previously received ICIs, for those with PD-L1 expression ≥50%, recommended subsequent systemic treatments include single-agent ICIs,^415,436^ or immunotherapy combined with single-agent chemotherapy± antiangiogenic therapy. These recommendations are supported by robust data from the initial entry of ICIs into clinical practice. Secondly, for patients who fail single-agent immunotherapy in the first line, and have not received chemotherapy, notwithstanding this scenario may not have been extensively studied in clinical trials, there is a strong consensus suggesting the use of chemotherapy with a platinum backbone, similar to first-line recommendations, alongside continued checkpoint inhibitor therapy^437^ or anti-angiogenic therapy,^438^ which represents an innovative and promising approach, and one that has been actively explored.

Nevertheless, in the current clinical practice and trials for metastatic NSCLC, over 90% of treatments are based on combination therapies involving ICIs in the first-line setting,^439^ consequently, leaving us to have to deal with a truly vexing group of patients who are resistant to treatment.^440^ And indeed, given that the prospective randomized controlled phase III trials for this patient cohort have failed to substantiate the efficacy of cabozantinib combined with atezolizumab,^438^ canakinumab (IL-1β blocking) combined with docetaxel,^441^ or sitravatinib plus nivolumab against docetaxel,^442^ the selection of optimal second-to-third line therapeutic regimens becomes a more complex and pressing clinical issue. For non-squamous cell carcinoma patients who have not received targeted treatment with anti-VEGF/VEGFR, bevacizumab^443^ in combination with taxanes, or pemetrexed,^444^ represents a suitable choice. Other taxanes, such as albumin-bound paclitaxel combined with bevacizumab,^445^ may also serve as alternatives, though the subjects in these studies were not specific to ICI-resistant patients.

Following the failure of first-line immunotherapy, chemotherapy in combination with anti-angiogenic agents such as ramucirumab or nintedanib continues to represent a reasonable and informed treatment option,^446^ although merely supported by evidence from smaller, single-center studies or retrospective analysis of the Flatiron Health database, and the ANSELMA meta-analysis.^447^ The combination of docetaxel, ramucirumab, and pembrolizumab is currently being evaluated in treating disease progression after response to platinum doublet therapy and PD-1/PD-L1 blockade.

##### Low-intensity treatment

For metastatic NSCLC patients experiencing disease progression after frontline ICI plus chemotherapy, the more common scenario involves patients with deteriorated PS conditions. Thus, low-intensity treatment options are necessary, guided by pre-immunotherapy era research findings. For patients with late-stage non-squamous NSCLC who have not been treated with pemetrexed previously, monotherapy with pemetrexed follows a high-evidence-based approach,^448,449^ alongside the use of liposome-bound paclitaxel or multi-target anti-angiogenic inhibitors administered singularly. Meanwhile, for those with squamous histology, gemcitabine, or docetaxel or de novo ICIs monotherapy,^415,436,450^ had also been demonstrated effectiveness. Recently, a multicenter phase II clinical trial^451^ demonstrated that nanoparticle albumin-bound paclitaxel as monotherapy can improve the ORR in NSCLC patients with ICI treatment failure.

Secondly, switching to alternative immunotherapies, exemplified by the combinations of atezolizumab and ipilimumab^452^ or durvalumab and ceralasertib (an ATR kinase inhibitor) in the phase II umbrella HUDSON study^453^ for treating metastatic NSCLC, have shown initial promise, whereas the use of durvalumab alone or in tandem with tremelimumab failed to notably enhance OS or PFS.^454^

Notably, a recent phase III international randomized controlled trial (DUBLIN-3) confirmed that plinabulin, an immunomodulator acting on microtubules, in combination with docetaxel as a later-line treatment for NSCLC patients without driver mutations, could be considered a new treatment option. This was supported by the OS being 15.1 months, compared to 12.8 months in the control group (*P* = 0.03), although grade 3 or 4 gastrointestinal disorders occurred more frequently in the plinabulin group.^455^

### Challenges

In the field of pharmacological treatment for advanced NSCLC, although significant progress has been made over the past 20 years, initially seen in adenocarcinoma, particularly among East Asian female patients, followed by a small subset of patients benefiting from immunotherapy, even with some achieving cures. Despite these, there remain significant gaps – low response breadth across a wide range of NSCLC patients and low response depth relative to the decades-long survival expectations of these patients. We believe that a primary focus on the design of clinical trials will be required in the future, as currently, most lack rigorous molecular biomarker screening, especially of most immunotherapies, leading to substantial differences in efficacy among patients, with extreme efficacy in a few individuals boosting statistical power. Fortunately, efforts to enroll patients based on biomarkers are increasing.^240^

Secondly, there is now an increasing trend toward using short-term survival endpoints such as PFS as surrogates for drug efficacy assessment, but this is a double-edged sword.^456^ The advantage is that it accelerates drug approval and reduces development costs, but critics argue that there is no inherent link between PFS and OS determination, and that tumors may later accelerate growth due to resistance, making up for previous deficits. In addition, another possibility is that patients crossover to the study drug treatment group, or they receive other effective treatments after exiting the study – after all, there are still many local and systemic treatment strategies available. However, for populations in low- and middle-income countries, the likelihood of receiving additional effective treatments is minimal, leading to a scenario where a single treatment strategy determines the length of life – one of the reasons why sponsors prefer these regions or populations for their studies.^457^ Therefore, the socioeconomic conditions of trial participants need to be considered during the randomization process, and also be reasonably matched in international studies.

Thirdly, most control groups in these studies consist of so-called standard-of-care treatments, such as chemotherapy or first-generation TKIs. Consequently, recently approved drugs do not appear to be optimal, although their availability can be improved through competition. However, there are also pioneers that are being compared against the latest treatments, such as osimertinib or pembrolizumab,^458^ and promising results were obtained. Fourthly, while biomarkers can guide the selection of the optimal treatment plan, there are significant differences in PFS survival benefits among drugs targeting the same mechanism, such as third-generation ALK inhibitors (Table 2) or ICIs. Therefore, addressing this dilemma requires head-to-head comparisons of different drugs with similar or identical targets, even though there may not be many at present.^459^ Moreover, there is often a delay in drug withdrawals in many regions.^460^

Finally, and perhaps most crucially, a deeper understanding of various resistance mechanisms can inform the expansion of the drug treatment arsenal to further maximize tumor eradication. This will be discussed in detail in the following sections.

## NSCLC treatment resistance and associated mechanisms

**Fig. 10:**
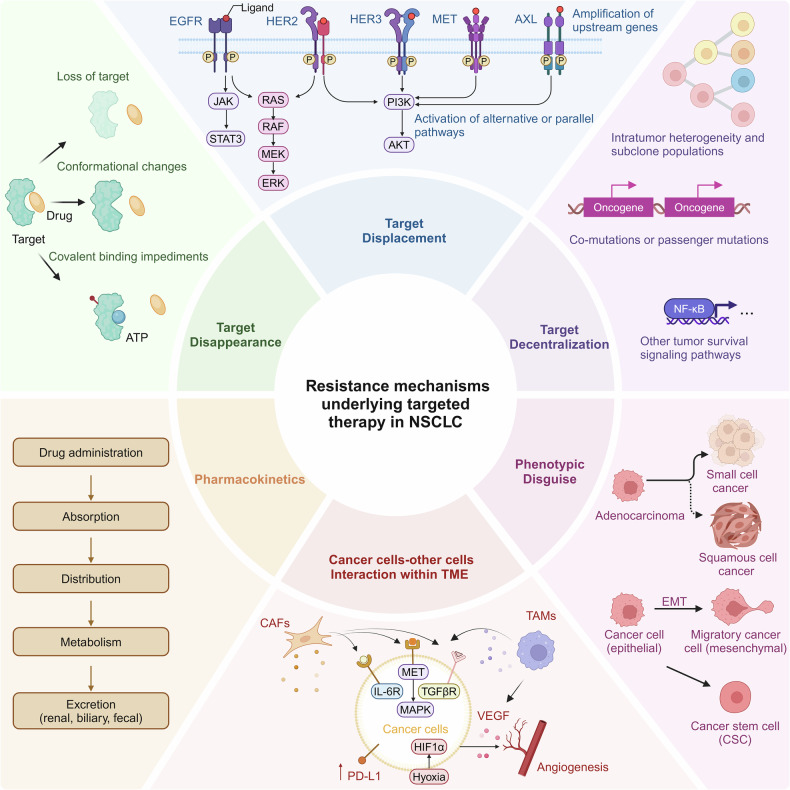
Resistance mechanisms underlying targeted therapy in NSCLC. The disappearance of target sites can be explained by two scenarios: the loss of target sites due to genetic mutations, or the loss of affinity between the drug and the target site due to genetic or epigenetic modifications. More importantly, the distinction between substitution and decentralization is blurred, as partial substitution can also lead to multiple pathways that provide survival signals to cancer cells – a strategy akin to how humans diversify investments to mitigate risks. The illustration lists several factors contributing to resistance, but it is crucial to acknowledge that many unknown mechanisms of resistance against targeted therapy remain

**Fig. 11:**
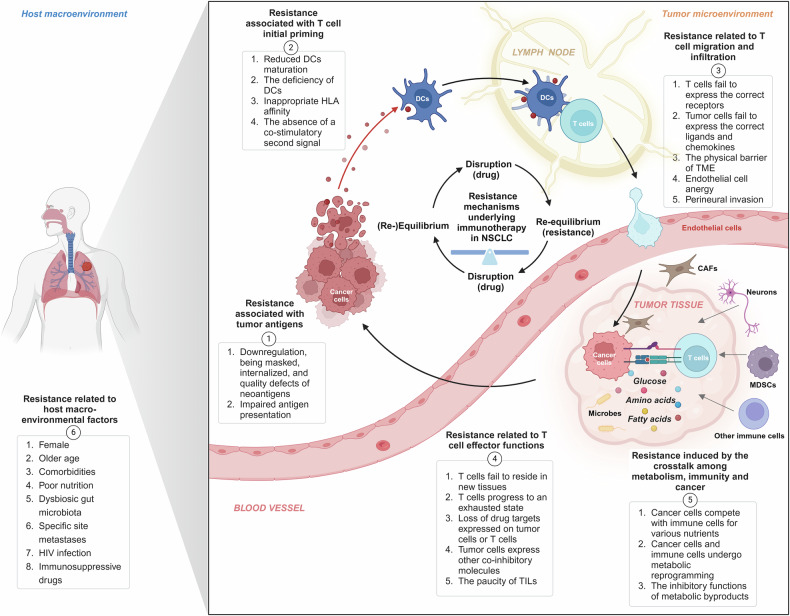
Resistance mechanisms underlying immunotherapy in NSCLC. Cancer immunotherapy may involve some of the most complex resistance mechanisms known, affecting nearly all cells, molecules, and pathways. The vast genetic and epigenetic abnormalities, estimated to involve approximately 1000 genes – including various cytokines, chemokines, protein kinases, and metabolic enzymes – result in T cells tolerating or coexisting with cancer cells. Therefore, we propose a new theoretical framework for understanding the mechanism of immunotherapy resistance, characterized by a dynamic and cyclical process: an equilibrium/balance favoring cancer cells (primary resistance), followed by treatment shifting the balance towards favoring immune cells, and eventually a re-establishment of equilibrium favoring cancer cells (secondary resistance) – which repeats over time

Over the past two decades, significant advancements have been made in the assessment and treatment of NSCLC, yet several challenges persist, with the most critical being drug resistance.^295^ In NSCLC, cancer cells within the TME act as evolving preys targeted by drugs, particularly where therapeutic selective pressures accelerate this evolution, inevitably leading to resistance against treatments. Drug resistance in cancer generally involves the intrinsic characteristics of drugs, the tumor cell population, and the specific TME, either individually or in complex combinations (Figs. 10 and 11).

### Resistance mechanism of TKIs

Clarifying the resistance mechanisms in NSCLC remains an unresolved challenge.^266,461^ The binary description of primary and acquired resistance to cancer is merely a clinical temporal concept and the definitions of intratumoral and extratumoral resistance lack depth in defining the underlying mechanisms. Hence, to elucidate the fundamental biological principles governing resistance to current and future cancer therapies, we propose a framework consisting of ‘disappearance’, ‘displacement’, ‘deconcentration’, and ‘disguisement’ to provide a detailed account of the biological determinants of resistance (Fig. 10). It must be acknowledged, however, that various mechanisms are artificially delineated, nevertheless this does not deny their intricate interconnections. Furthermore, substantial progress has been made in understanding TKI resistance, yet the resistance mechanisms in a significant number of patients remain unknown (up to 40-50% following first-line osimertinib treatment).^272^ The aforementioned resistance mechanisms predominantly arise in monotherapy settings. In combination therapy, however, there may be occasional minor deviations, though the majority remain similar.

#### Target disappearance

One scenario of target disappearance involves the cessation of expression, which is a major cause of acquired resistance to TKIs (and inevitably primary resistance as well, instead of the bull’s-eye hitting the arrow), exampled by the disappearance of T790M mutation in patients resistant to osimertinib treatment,^462^ often associated with a shorter survival time.^463^ Another scenario of target disappearance involves the loss of accessibility, which results from a series of mutations in the kinase domains, such as T790M mutation in EGFR, L1196M mutation in ALK and L2026M mutation in ROS1, leading to conformational changes – the disappearance of the original TKI binding pocket. The second challenge case involves covalent binding impediments, such as EGFR^C797S^ mutation, which leads to resistance against third-generation osimertinib. Other instances involve rare solvent-front mutations causing steric hindrance, including but not limited to G796X, L792X, L718X and G724S mutations^464^ in EGFR, or the G1202R, L1256F, G1269A, V1180L, I1171X, D1203N, S1206Y/C, E1210K, C1156Y, I1171T, and V1180L mutations, and the other co-mutations in ALK,^465^ as well as V573M, F589L, and G667C mutations in NTRK.

#### Target displacement

Survival resilience – the capacity of systems to maintain functionality in the face of both external and internal perturbations – is a core feature of cancer. Consequently, when components of critical cellular pathways like EGFR and ALK are targeted, cancer cells exhibit resistance through displacement responses, with a common mechanism being the activation of alternative pathways, which can involve reactivation of the pathway itself (through upstream or downstream events), or engagement with nodes facilitating bypass of oncogenic signaling effects. As a crucial example, the RAS-RAF-MEK-ERK, PI3K-AKT-mTOR, and JAK-STAT3 pathways are key downstream effectors of EGFR and ALK, which can be reactivated through various mechanisms such as KRAS mutations, NRAS mutations, MAP2K1 mutations, DUSP6 loss or gain, RAF mutations, BRAF fusions or the absence of neurofibromin1 (NF1), which lead to resistance against various RTK inhibitors. Another displacement involves the upregulation of upstream genes within intrachromosomal or extrachromosomal DNA,^466^ which can confer resistance against downstream molecular targets, such as MET amplification and RET rearrangements, observed in patients resistant to osimertinib or lorlatinib therapy,^467^ thus, overcoming such resistance can be achieved through combination therapies. Similarly, the displacement outside of targeted pathways, such as loss of PTEN or mutations in genes encoding PI3K, leading to constitutive activation of the PI3K pathway, provides alternative survival signals contributing to drug resistance.^468^ Furthermore, mechanisms of resistance to anti-angiogenic drugs can involve redundant angiogenic signal transduction and activation of compensatory angiogenic pathways, such as increased expression of factors like IL-8, IL-17, and apelin (APLN), activation of the IL-6/STAT3 signal cascade, or displacement through normal vessel borrowing or angiogenic mimicry.

#### Target decentralization

Genotypic features driven by mutations or incomplete repair and replication of chromosomal DNA or extrachromosomal DNA (ecDNA),^469^ along with epigenetic features modified by environmental stress, have been inherited in cancer cell populations over many years via branched clonal evolution or horizontal transfer.^470^ As a natural consequence, tumors composed of billions of cells exhibit spatial and temporal phenotypic diversity, which is a conceptually straightforward mechanism of drug resistance. A common example is a housekeeping or driver mutation within a single gene providing survival signaling, to which cancer cells become addicted, simultaneously or asynchronously accompanied by other genetic alterations, called as co-mutation or passenger mutation^471^ which also provide some level of survival signaling – a main mechanism of primary drug resistance. In patients with EGFR mutations, co-mutations or other genetic abnormalities minimally include TP53, RB1, CTNNB1, and PIK3CA, as well as amplifications involving EGFR, NKX2-1, and CDK4, or activation of AXL and CDCP1.^472^ Similarly, ALK, RET and ROS1 rearrangements, and BRAF, RAS, ERBB2^461^ and MET exon 14 skipping mutations in late-stage NSCLC, are also characterized by a high number of co-mutations, like in CDKN2A/2B, TP53, LKB1, KEAP1, NRAS, BRCA1/2^266^ and BRAF.^473^ So far, the spectrum of co-mutations in NRG1 or NTRK1 fusion events in LUAD has not been elucidated.

A more advanced perspective is that the traditional binary distinction between passenger mutation and driver mutation is also challenged, given that the former may also release primary survival signals. Therefore, the above or other considerable evidence indicates that during targeted therapy,^474^ intelligent cancer cells disperse survival pressures to be addicted to multiple low-functioning mutations rather than a single driver mutation – a process of decentralization, characterized by obvious intratumor heterogeneity,^475^ and various secondary subclone populations.^476^ Moreover, decentralization, which engages all mechanisms associated with drug resistance, presents the most formidable challenge to be overcome in a concentrated manner^477^ – due to multiple targets, although their occurrence is hardly synchronous in individual patients.

#### Phenotypic disguise

Adaptive mechanisms within cancer ecosystems, beyond signaling pathway regulation, can also involve epigenetic or non-genetic negative feedback rescue processes, leading to lineage plasticity and the emergence of new histological types, which are crucial contributors to drug resistance. The newly-emerged phenotypes act like disguises, characterized by the transformation of adenocarcinomas into small cell lung cancer (SCLC, comprising 3–14%, mainly related to epigenetic alterations^478^) or squamous cells or a transcriptional state (with mucinous histologic feature^479^) during progression,^266^ however, the primary driving mutations remain present, yet there is a loss of sensitivity to TKIs,^480^ or to chemotherapy. Whether the prevalence of tumor cell plasticity and histological transformation or disguise varies across different NSCLC molecular subgroups is currently unclear. However, NSCLC patients with baseline RB1 and TP53 inactivation, high mutational features of the apolipoprotein B mRNA editing enzyme, and alterations in PIK3CA^481^ have a significantly elevated (43-fold) risk of disguising as SCLC during TKI therapy.

Furthermore, cancer cells in response to unfavorable conditions within the TME, such as hypoxia, inflammation, or exposure to targeted therapies, may adopt phenotypic disguise through resembling basal-like/stem-like cells, drug-tolerant persister (DTP) and EMT, all of which contribute to the development of drug resistance in NSCLC patients.^482^ The mechanism might underlie the activation of signaling pathways like ATR-CHK1-Aurora B,^483^ APOBEC mutagenesis, epigenetic modifications including the upregulation of histone methyltransferase enhancer of zeste homolog 2 (EZH2) or the activation of RE1-silencing transcription factor,^461^ and the activation of HIF-1α related IGF1R.

Given these complexities, to perform repeated tissue biopsies (if feasible) is strongly recommended, due to the limitations of current liquid biopsy platforms, which often fail to detect such histological or phenotypic disguises or transformations.

#### Other factors

The dynamic interactions between tumor cells and non-malignant cells within the TME also influence resistance to TKI therapies. CAFs upregulating anti-apoptotic genes such as BCL2, SerpinB2 (a protein that inhibits peripheral tissue proteases), TAMs activating MAPK, PI3K, YAP, NF-κB, WNT, and RAS pathways, endothelial cells secreting EGF, TGF-α, heparin-binding EGF like growth factor (HB-EGF) and hepatocyte growth factor, and T cells highly expressing CTLA4,^484^ as well as upregulating ECM related adhesion molecules, such as N-cadherin and integrin β 1, C-X-C chemokine receptor 4 (CXCR4), and C-X-C chemokine ligand 12 (CXCL12), all of these are factors believed to be associated with TKI resistance.

The levels and exposure dynamics of drugs which are influenced by multiple patient-specific pharmacokinetic factors, encompassing absorption, distribution, metabolism, and excretion, can also impact the resistance to targeted therapies used in clinical settings. Additionally, drugs that induce cytochrome P450 3A4, can lead to an upregulation of genes encoding P-glycoprotein efflux pumps, potentially causing resistance.^485^ Abnormalities in pharmacological processes related to ADCs, such as the uptake of the drug into tumor cells, which depends on the endocytosis of the targets,^486^ and the lysosomal transport and release of the payload, may also contribute to drug resistance.

### Resistance mechanism of immunotherapy

Resistance to immunotherapies, mainly ICIs, is more intricate, unfolding through dynamic shifts in the delicate equilibrium between the tumor and the immune system^487^ and can vary spatially and temporally, compared to resistance to targeted therapies, which tends to be more closely tied to the cancer cells themselves (Fig. 11).^488^ We also propose a new perspective that essentially, the immunotherapy resistance is a rebalance process characterized by a cycle from balance/equilibrium formation to subsequent disruption – prior to treatment, the balance tends to favor cancer cells,and then treatment disrupts this balance, favoring antitumor immunity and again the balance re-establishes in favor of cancer cells (drug resistance emerges) – repeating over time.^489^

From a mechanistic standpoint, the comprehending resistance to cancer immunotherapy requires insight into how cancer cells are eliminated by the immune system, particularly by effector T cells. In simple terms, the process of antitumor immunity involves such steps as the release of tumor cell antigens, presentation of these antigens by DCs within lymph nodes to T cells, migration and infiltration of T cells into the TME, followed by survival, recognition, and killing of cancer cells.^490^ Moreover, within the TME, TILs also undergo sub-cycles of proliferation, exhaustion, and effector function.^491^

As a whole, within the TME of NSCLC, although immune-promoting cells such as CD8^+^T cells CD4^+^helper T cells, natural killer (NK) cells, invariant NK T (iNKT) cells, and γδ T cells, as well as DCs, form the cornerstone of antitumor immunity, other immunosuppressive cells,^492^ such as T_reg_s, mast cells, eosinophils, MDSCs and lymphatic endothelial cells (LECs), adipocytes, neurons, themselves or under hijack by cancer cells,^493^ are reprogrammed to produce nitric oxide and reactive oxygen species, release adenosine, induce immunosuppressive checkpoints, growth factors and cytokines, thereby disrupting T cell transport, proliferation, persistence and effector function, which lead to resistance against immunotherapies.^494^ Moreover, each cell can serve as an input and output, exerting regulatory functions, such as TAMs through upregulation of arginase I, secrete the plate factor 4 induced T_reg_ cell polarization into immune suppressive TH1-T_reg_ cells.^495^ Notably, our sub-classification of resistance mechanisms is not intended to deny the intertwined connections between different categories but rather to facilitate understanding. Furthermore, the various molecular, or cellular pathways involved in resistance can exhibit broad, pleiotropic, and even opposing immunomodulatory effects depending on their temporal and spatial states. For example, inert versus potent IL-33 signaling,^496^ acute versus chronic activation of STING-IFN signaling pathways,^102^ and the innate immune cell axis (namely, innate lymphoid cells and innate-like T cells, in parallel)^497^ or the well-known TAMs and tumor-induced B cells,^498^ can promote or inhibit antitumor immunity due to differential reprogramming regulation. Finally, apart from inter-individual variations, malignant cells in different tumor regions or at various time points might evade immune recognition and elimination through distinct mechanisms.^85^ This implies that, at least in some cases, simultaneously and highly flexible suppression of different immune escape mechanisms could be more efficacious than fixed targeting of a single mechanism, potentially leading to more effective therapeutics.

#### Resistance associated with tumor antigens

Genetic and epigenetic variations throughout the life cycle of cancer cells, driven by intrinsic evolution and drug-induced selective pressures,^499^ act as a double-edged sword in cancer immunotherapy.^493^ On one hand, variations that lead to defects in DNA repair genes, such as ATM, POLE, FANCA, ERCC2, and MSH1 and 6, and subsequently result in high TMB or MSI^high^, or dysregulated mRNA splicing and modifying create new epitopes^500^ that are associated with high sensitivity to immunotherapies. On the other hand, variations also contribute to the prevalence of cancer cell subclones, characterized by downregulation of neoantigens or neopeptides^499^ expression, neoantigens being masked and internalized, or quality defects of neoantigens induced by instability and a short lifespan, certainly, this could also be attributed to the early elimination of sensitive clones^499^– a process of immune editing.The process is the main reason for immunotherapy resistance in NSCLC. The mechanisms underlying the immune editing are not fully understood but could be attributed to the excessive methylation of gene promoters that code for neoantigens,^501^ or the loss of gene copies that encode core mutated segments or chromosomal deletions. Secondly, various HLA homo- or heterologous deletions, genetic and non-genetic HLA disruption,^502^ decreased HLA molecular diversity,^503^ loss of β-2 microglobulin (B2M), tapasin and transporters associated with antigen processing (TAP) expression by mutations along with epigenetic alterations, collectively contribute to insufficient or incomplete presentation, and potentially lead to resistance against immunotherapies. Additionally, the loss of drug targets, such as the absence of B7-H1 or PD-1/PD-L1 expression in either tumor cells or T cells, can hinder the effective operation of ICIs in treating tumors.

#### Resistance associated with T cell initial priming

DCs remain indispensable in anti-cancer immunity, being capable of initiating and amplifying specific antigen-specific CD4^+^ and CD8^+^T cell responses. DCs must receive pro-inflammatory or inflammatory stimulatory signals to prime the terminal differentiation process, transitioning DCs from an antigen accumulation into an antigen-presenting.^503^ Conversely, reduced DC maturation due to insufficient inflammation in the TME or decreased cDC1-CD8^+^T cell interactions resulting from ALCAM downregulation^504^ may induce immune tolerance through suppressing the activation or inducing exhaustion of T cells. Certainly, “immuno-desert” tumors, marked by a lack of T-cell infiltration, have demonstrated minimal response to immunotherapies, primarily due to the deficiency of DCs^491^ and importantly, an mRNA vaccine targeting DCs that encode claudin-6 has been found to enhance the immunological function of CAR-T cells.^505^

For the initial priming of T cells, sufficient antigen presentation (such as cross-presentation) by antigen-presenting cells (APCs) is required,^506^ which can also be blocked through upregulation of CD47 expression on the malignant cell membrane.^507^ However, appropriate affinity is necessary for effective T cell priming, so as to allow persistent contact and residence, as overly high affinity can lead to tolerance or anergy of T cells. Beyond affinity, further CD8^+^T cell priming and activation depends on the amount of TCR signaling resulting from TCR-pHLA interactions in equilibrium,^506^ followed by a co-stimulatory second signal and avoidance of T_reg_ cells.^508^ Consequently, breaking existing tolerances via antigen release and inflammation induced by immunogenic cell death (ICD), or/and ICI blockade, facilitates T cell priming or activation.^509^ In addition, tumor cells can evade NK cell elimination by downregulating various NK cell activation ligands such as MHC class I peptide-associated sequence A, or B (MICA or B), and UL16 binding proteins.^496^

#### Resistance related to T cell migration and infiltration

Primary T cells, anatomically constrained to secondary lymphoid organs (including draining lymph nodes) and circulation. Once activated within these organs, T cells undergo a dynamic, finely coordinated, and meticulously choreographed migration to the TME, leading to the formation of TILs, which are distinct from intratumoral resident T cells, and exert contact-dependent killing of cancer cells presenting peptide-MHC-I complexes.^107,510^ Thus, draining lymph nodes serve as a sanctuary for T cells, with substantial clinical evidence indicating that extensive lymphadenectomy is associated with worse prognosis, instead, ICIs administered as neoadjuvant therapy have been linked to higher response rates.

There are numerous impediments to the recruitment of T cells into tumors, which could be intrinsic to T cells, or attributable to vascular endothelial cells and/or tumor cells failing to express the correct ligands and/or chemokines,^511^ as well as inhibitory pathways that suppress the production of inflammatory factors (like the IFN signaling pathway). Indeed, in patients with LUAD, a notable rise in recurrence risk correlates with lower T cell infiltration and a higher ratio of T_reg_s to TILs, and the underlying mechanism could involve resistance to immunotherapies.

The first challenge is that T cells fail to upregulate integrins of αLβ2 (binding to ICAM) and α4β1 (binding to VCAM1), as well as chemokine receptor CXCR3 (a receptor for CXCL9, CXCL10, and CXCL11) and CCR5 (a receptor for CCL3, CCL4 and CCL5) after activation, and even tumor cells inhibit macrophage production of CXCL9. Secondly, several inhibitory cytokines, such as TGFβ and IL-33, released by malignant cells, hamper the establishment of CD8^+^T cell tissue residency and tissue-specific adaptation,^512^ instead of claudin 18 which orchestrates T cell infiltration. Thirdly, the physical characteristics of the TME primarily orchestrated by the quantity and hardness of CAFs and cancer cells, including increased extracellular matrix density, and interstitial pressure through matrix deposition and cross-linking, pose significant barriers to the infiltration of CD8^+^T cells. Moreover, CAFs can also express TGF-β, IL-6, CXCL1 and CXCL12, PGE2, and netrinG1, to recruit immune suppressive cell populations (such as TAMs, TANs and MDSCs), suppress the infiltration of cytotoxic T cells, and reduce NK activation, thereby promoting the formation of immune suppressive cycles and immune therapy failure. However, while various preclinical and clinical studies targeting CAFs are underway, results have been inconsistent because CAFs exhibit dual functions of promoting and inhibiting the immune system^513^ in different TME and throughout the evolution of cancers.^514^ Fourthly, disordered tumor vessel formation, lacking proper pruning, and resistance to upregulation of inflammatory chemokines and integrin ligands due to persistent VEGF stimulation -which impedes T cells from effectively binding to endothelial cells and penetrating into the TME (a phenomenon known as endothelial cell anergy),^511^ all serve as a physical barrier to cell migration. Lastly, newer studies have shown that sensory neural input can enhance macrophage anti-inflammatory effects via S1P, facilitating the egress of innate and effector T cells, leading to immunosuppression or an “immunological cold” microenvironment,^515^ and inhibiting tumor-killing function of T cells in a metabolite and neurotransmitter GABA-dependent manner.^516^ Additionally, hyperactivation of MAPK signaling in cancer cells impairs the inflammatory monocyte state and intratumoral T cell stimulation by coordinately blunting the production of type I interferon and inducing the secretion of prostaglandin E2.^517^

#### Resistance related to T cell effector functions

Understanding the mechanisms of drug resistance in cancer immunotherapy requires a deep dive into the differentiation process of T cells and the various terminologies used to define T cells, despite their apparent overlaps that lead to notable heterogeneity across different publications. Once experiencing antigen, T cells differentiate into distinct T_eff_ cell and memory T cell (T_Mem_) populations, with the latter providing long-term immunity. The T_Mem_ cell population comprises subgroups at various stages of differentiation, at least including stem cell memory T (T_SCM_) cells, central memory T cells, effector memory T cells (T_EM_ cells), and tissue-resident memory T (T_RM_) cells, although these can occasionally be found at high frequencies in the blood, secondary lymphoid organs, and peripheral tissues. The second largest category in the TME is made up of effector T cells, which are exhausted T cells (T_EX_) with different functional states due to chronic antigen stimulation or increased IFN-γ response driven by ICI therapy.^518^ These cells display high expression of coinhibitory molecules such as PD-1, CTLA-4, LAG-3, TIM-3, and CD39 (encoded by ENTPD1) among others that represent redundant or compensatory pathways for immune evasion and treatment resistance, encompassing pre-exhausted T (T_PEX_) cells, TRM-like cells, and terminal exhausted T cells (T_TE_) and are rarely found in the circulating blood and instead localized to niches with a large number of their cognate antigens.

Cancer is a complex ecosystem, characterized by cancer cells that are enveloped by various non-cancer cell types, alongside the growth factors, cytokines, chemokines, kinases, and proteases that they secrete, collectively forming a highly structured, vasculated, nutrient-deprived, and hypoxic TME.^492^ Consequently, once T cells migrate and extravasate into the TME, they must confront significant challenges posed by this distinct milieu, necessitating critical adaptive adjustments- many of which are driven by transcriptional and epigenetic changes.^519^ T cell differentiation firstly involves downregulating cellular mechanisms that promote tissue departure (including S1PR1) and upregulating adhesion receptors required for interaction with the local microenvironment (such as CD103), along with transcription factors (including HOBIT, BLIMP1, EOMES, T-bet, and RUNX3). This adaptation enables them to reside in new tissues, facilitating their effector functions. Indeed, these regulatory abnormalities within T cells themselves or mutations in other oncogenic pathways within cancer cells, such as EGFR, ALK, ROS1, RET, and cMET,^520^ can also contribute to immunotherapy resistance, such as through upregulation of CD47.

Remarkably, subpopulations of T_PEX_ cells within the T_EX_ cells are not functionally quiescent, rather less functional impairment and subject to fewer epigenetic constraints, making them more responsive to PD-1 inhibitors.^521^ However, restoring the cytotoxic potential and plasticity of CD8^+^ T cells once they have progressed to a T_TE_ state, is challenging, notwithstanding they can still proliferate antigen-dependently, thereby explaining why antitumor immunity might be limited when only targeting the predominant T_TE_ cells in the TME.^522^ What needs to be emphasized is that the differentiation or exhaustion of T cells within tumors is a progressive process, not an independent sequence, hence, there are diverse T cell states that lack clear demarcations.

T effector cells, have been shown to engage ligand-receptor interactions with other immunocytes entering the tumor, such as CD4^+^ helper T cells and CD20^+^ B cells through spatial crosstalk in the TME^523^ or TLS, which are observed in lung cancer or other cancers (with certain exceptions),^524^ and associated with better responses to checkpoint blockade treatments. T_PEX_ cells are commonly found within the TLS, maintaining their ability to self-renew and differentiate through supportive interactions with other immune cells, serving as a reservoir to replenish T_TE_ cells. Conversely, NSCLC cases with liver metastases, are often characterized by poor response to ICI therapies, possibly due to the high presence of T_reg_ cells within the liver microenvironment. Lastly, a least in certain cases, cancer cells reduce cytotoxic sensitivity to GZMB and PRF1 through biomechanical properties, such as insufficient cellular contraction mediated by the actin cytoskeleton.^525^

In summary, each major step during T cell fate trajectory responds to cancer – from the productive activation of T cells in draining lymph nodes to their migration and infiltration into the TME, and adaptation to the TME for survival – providing opportunities for immune escape, however, also revealing vulnerability for therapeutic targeting. For instance, alleviating metabolic constraints, nutritional deficiencies, and/or hypoxia might enhance the survival and effector function of T cells, thereby offering potential clinical benefits.

#### The resistance induced by the crosstalk involving metabolism, immunity and cancer

From Warburg’s discovery of changes in cancer metabolism over a century ago, to Sidney Farber’s introduction of folic acid therapy for childhood leukemia in 1948, followed by two decades ago when the relationship between metabolism and oncogenes was elucidated, the study of interactions across metabolism, immunity, and cancer has largely unfolded in parallel. However, in recent years, the three domains have been converging, owing to discoveries that inhibiting specific metabolic pathways can impact every cell in the TME, thus suppressing or promoting tumor progression. Firstly, cancer cells are characterized by high consumption of resources, providing vulnerabilities for pharmacological interventions, which inevitably compete with surrounding immune cells for various nutrients including oxygen, amino acids, glucose, and fatty acids, thereby compromising function of the latter. Secondly, to adapt to harsh conditions, immune cells alongside cancer cells engage in rapid and extensive metabolic reprogramming through shared mechanisms to compete for energy, including the upregulation of glycolysis and glutamine metabolism, fatty acid oxidation, and tryptophan synthesis. Lastly, a plethora of metabolic byproducts such as lactate and peroxides (like NO), coupled with hypoxia, and low levels of glucose, folate, and essential amino acid, significantly inhibit the phenotype development and proliferation capacity of immune-effector cells.^526^

##### The metabolism of cancer cells

Many common oncogenic driver gene mutations directly regulate metabolic pathways, not only promoting tumor growth but also potentially leading to therapeutic resistance.^527^ For instance, in cancer mouse models, KRAS primarily induces metabolic reprogramming in cancer cells through the transcription factors HIF-1α and c-MYC, or concomitant LKB1 loss, leading to the upregulation of glycolysis, fatty acid and glutamine metabolism, together with lipid synthesis. In LUAD, mutations in Kelch-like ECH related protein 1 (KEAP1),^528^ and p53, structurally activate the transcription factor Nrf2, leading to increased expression of serine synthesis genes, upregulation of the cystine transporter SLC7A11, and elevation of xCT (the xc-cystine/glutamate exchanger) to maintain intracellular cysteine levels that support glutathione synthesis. This is associated with the reduced efficacy of immunotherapeutic interventions. H3K18la (histone H3 lysine 18 lactylation, downstream glycolysis) potentiates the immune escape of NSCLC cells by activating the POM121/MYC/PD-L1 pathway.^529^ The expression of PD-L1 in cancer cells can drive the activation of Akt-mTOR and glycolysis, increasing glucose uptake and then enhancing their ability to compete with T cells for glucose. Isocitrate dehydrogenases (IDHs), the most studied enzymes, act as rate-limiting enzymes in the tricarboxylic acid (TCA) cycle and are involved in cellular energy metabolism. More than 80% of mutations in IDH1/2 are found in World Health Organization grade II/III gliomas, whereas accounting for only 0.5%-3% in lung cancers and other solid tumors. The activation of IDH1/2 or IDO involved in tryptophan catabolism through reprogramming or mutation within the catalytic site of the enzyme, not only promotes cancer cell proliferation and migration, but also compromises the intensity and quality of T cell responses against cancer cells, thus contributing to the development of resistance to immunotherapies in NSCLC and other cancers,^530^ without doubt providing potential therapeutic targets.^531^

##### The metabolism of immune cells

Specific nutrient deficiencies in the TME severely limit the metabolism processes of highly proliferative immune cells (such as effector T cells), like protein and nucleotide synthesis. Therefore, amino acid transporters, including SLC7A5 (also known as LAT1), SLC38A1 (also known as SNAT1), SLC38A2 (also known as SNAT2), and SLC1A5 (also known as ASCT2), are highly upregulated in T cell with antigen experience,^532^ This upregulation enhances antitumor immune responses, if otherwise, immunotherapy resistance occurs. Lipid metabolism reprogramming also affects virtually all cell types, including CD4^+^T, CD8^+^T (like T_Mem_), NKT cells, NK cells, M1/M2 TAMs, DCs, and N1/N2 TANs. This reprogramming occurs through the upregulation of transcription factors SREBP1 and SREBP2, which promotes cholesterol uptake and drives the lipid synthesis cascade, and influences key processes such as oxidative phosphorylation. Therefore, the lack of SREBP1 and SREBP2 might impair the function of CD8^+^T cells. T cell activation upregulates the transcriptional activity of MYC and HIF-1, promoting metabolic reprogramming and upregulating genes encoding enzymes involved in glycolysis, such as pyruvate kinase (PKM1), hexokinase 2 (HK2), and GLUT1, however, a deficiency of PKM2 can generate TCF1^+^progenitor CD8^+^T cells to improve immunotherapy efficacy.^533^ The metabolic reprogramming mediated by Fc-IL-4 is indispensable for reinvigorating intratumoral CD8^+^T_TE_ cells through augmenting the glycolytic metabolism and the concentration of nicotinamide adenine dinucleotide.^534^ Reducing the concentration of glucose in the medium not only hinders DC activation but also inhibits the production of critical effector molecules in CD4^+^ and CD8^+^T cells, such as IFN-γ, IL-17, and granzyme B, and diminishes T cell survival rates. Fructose-1,6-bisphosphate (FBP1) is a key enzyme in gluconeogenesis, and in cancer mouse, increased FBP1 expression strongly inhibits glycolysis, leading to impaired NK cell function and survival. Mitochondrial respiration is also a crucial aspect of T cell energy metabolism, and evidence from mouse models and human cancers suggests that tumor-infiltrating CD8^+^T cells reduce mitochondrial number or acquire mitochondrial DNA mutations from cancer cells^535^ to impair their function – an exhaustion sign.

Hypoxia can induce the expression of extracellular nucleotidases such as CD39 and CD73, which bring out the accumulation of adenosine in the TME,^536^ which broadly suppresses T cell effector functions and proliferation. Hypoxia-induced conditions in vitro lead to MDSCs acquiring immunosuppressive M2-like TAM phenotypes. However, the activation of HIF-1α enhances glycolytic activity, which paradoxically facilitates the formation of long-living memory T cells and antitumor activity.

##### Toxic metabolic products and their impact on immune cells

Anaerobic glycolysis within the TME generates substantial amounts of lactate, which cancer cells^537^ and partial immune cells such as T_reg_ cells and MDSCs can utilize to promote their proliferation and maintain their immunosuppressive function, like the differentiation of TAMs towards an M2 phenotype, thereby contributing to resistance against ICI therapies. Downregulation of suppressive T_reg_ through deletion of lactate transporter MCT1 to prevent lactate uptake, can result in increased proliferation and IFN-γ production in CD8^+^T and non-conventional T cells, followed by a reduction in tumor growth and an extended survival period.^538^ Conversely, lactate and the associated low pH within the TME can inhibit the proliferation, cytotoxicity, and cytokine production of NK and T_eff_ cells, disrupting the upregulation of key transcription factors like NFAT. Intermediate metabolites of the TCA cycle, such as α-ketoglutarate (α-KG), succinate, and fumarate, can hinder the differentiation of CD8^+^T memory cells.^539^ Furthermore, oncogenic metabolites (oncometabolites) including (R)- and (S)-2-hydroxyglutarate (2-HG), as well as D-2-HG and L-2-HG released from cancer cells can lead to disrupting the proliferation through blocking polyamine biosynthesis, TCR signaling and NFAT activity in both CD4^+^ and CD8^+^ T cells.^540,541^ The accumulation of specific amino acid metabolites within tumors, such as quinolinic acid produced by IDO1, can also impair T cell effector functions displayed as the lower production of IFN-γ and TNF.^542^ High levels of cellular death can lead to an increase in potassium concentration within the TME, which also restricts the functionality of T_eff_ cells.

Furthermore, different cancer cells exhibit varying dependences on metabolic products, despite being cultured under the same conditions. Moreover, distinct metabolic pathways, enzymes, and products have different functionalities. The case in point is that an imbalance in reactive oxygen species (ROS) levels can lead to cytotoxicity, but ROS can also stimulate IL-2 expression in both CD4^+^ and CD8^+^T cells.

### The resistance induced by inherent properties of drugs and treatment

The inherent immunogenicity of antibody drugs is one of the primary mechanisms for generating secondary resistance to antibody-based immunotherapies, such as CAR-T cells, ADCs, monoclonal or polyclonal antibodies, and bispecific T-cell engagers (BiTEs). Among the almost 100 therapeutic antibodies approved by the FDA, European Medicines Agency (EMA), and NMPA, the prevalence of anti-drug antibodies (ADAs) ranges from 0% to over 70%, owing to the presence of mouse-specific epitopes on early humanized antibodies. As a result, researchers have developed and tested humanized single-chain variable fragments (scFvs) to circumvent CAR-specific immune responses associated with murine scFvs.^543^ Changes in Fc glycosylation also influence the induction of ADAs, exampled as the removal of N-linked glycosylation has been shown to decrease immunogenicity. However, fully human monoclonal antibodies lacking Fc function have also been shown to be immunogenic, having direct detrimental effects on the recruitment of macrophages and the activation of the complement system. Moreover, the production of ADAs is also influenced by the patient’s HLA typing, disease type, and the repetition of excipients in the drug. For oncolytic viruses, their foreign antigens may be presented by DCs, to trigger virus-specific cytotoxic T cell responses and also be presented to CD4^+^ helper T cells, promoting B cells to produce neutralizing antibodies that ultimately limit viral spread and replication.

### The resistance induced by other factors

Other systemic factors of the patient, such as female gender, older age, poor nutrition, obesity (or muscle loss), and diabetes, gut microbiota dysbiosis, specific site metastases (such as brain and liver), certain accompanying therapies (like antibiotics or steroid hormones), HIV infection, and autoimmune diseases, are associated with a poor response to ICIs. Lastly, phenotypic changes, such as EMT, morphological changes, stem cell transformation, and proliferation arrest (quiescence), are important mechanisms underlying immunotherapy resistance (Figs. 10 and 11).^544^

## Developing strategies for overcoming resistance in NSCLC treatment

**Fig. 12:**
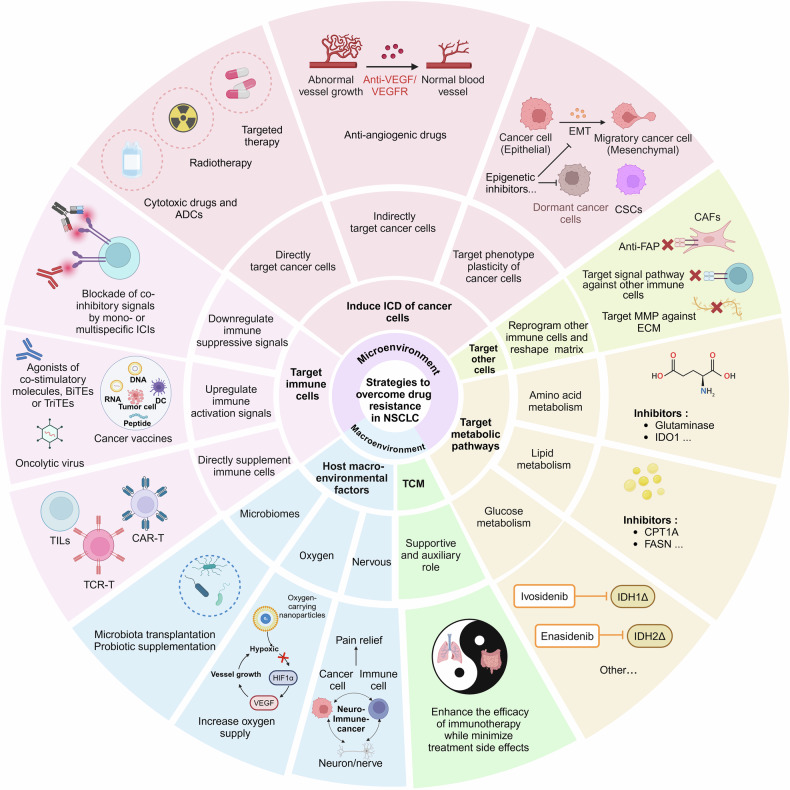
Established or developing strategies to overcome drug resistance in NSCLC. This diagram categorizes treatment drugs and strategies to facilitate logical understanding, though it may not be entirely accurate. A single treatment can overcome resistance through multiple mechanisms, and conversely, a single resistance mechanism may require multiple drugs for optimal combination therapy. Future developments should focus on combining multiple treatment regimens, particularly in immunotherapy, to overcome resistance. However, determining how to combine these treatments with other therapeutic approaches not shown in the diagram, such as surgery, radiotherapy, and local ablation, remains a challenge. It is important to note that even if some drug effects are not highly pronounced, if side effects are minimal, the cumulative effect can still be considerable. ICD includes apoptosis (in some cases), necroptosis, ferroptosis, and pyroptosis

Cancer, characterized by multifaceted systemic attributes,^6,114^ requires a multi-dimensional blockade to eradicate, involving diverse strategies. However, in this context, our primary focus is immunotherapy, considering the constraints of space and more importantly its extensive synergistic effects with other strategies,^6^ such as targeted treatment, chemotherapy (including ADCs), radiation, vaccines, OVs, as well as potential modulation methods on the host’s macro-system through diet,^545^ exercise, and gut microbiota(Fig. 12). We principally highlighted novel therapies that have entered early or later-phase clinical trials in the past several years, with promising developments.

### Directly targeting cancer cells

#### Cytotoxic drugs

For decades, the development of traditional chemotherapy was based on the ability to kill cancer cells in vitro or in highly immunocompromised mouse model, systematically overlooking the potential of the immune system to enhance the efficacy of cytotoxic drugs. However, numerous preclinical studies^366^ have shown that cytotoxic drugs, particularly when administered at moderate-to-low doses, not only lead to ICD of cancer cells, such as necroptosis, ferroptosis, and pyroptosis,^170,546,547^ but also deplete lymphocyte populations that suppress antitumor immunity, such as MDSCs, FOXP3^+^T_reg_s, and M2 polarized macrophages. Moreover, decreasing tumor size not only diminishes the number of cancer cells available for the immune system to eliminate, but also reduces the opportunities for immune evasion, which is often associated with larger tumor volumes. This strategy is supported by a substantial amount of clinical research on NSCLC, and highlights the advantages of immunotherapy in first-line rather than later-line settings.^353,423,548^

Current development of cytotoxic drugs in NSCLC focuses on enhancing delivery methods, such as formulating them as albumin-bound agents, or creating ADCs, which include trastuzumab deruxtecan targeting HER-2 mutations or overexpression,^181,549^ datopotamab deruxtecan^550^ and sacituzumab govitecan^551^ targeting trophoblast cell-surface antigen 2 (TROP2), telisotuzumab vedotin targeting c-MET,^552^ patritumab deruxtecan targeting HER-3,^553^ tusamitamab ravtansine targeting carcinoembryonic antigen-related cell adhesion molecule 5, mecbotamab vedotin targeting AXL, MRG003 targeting EGFR, and Ifinatamab deruxtecan targeting B7-H3^554^ (Table 5). These agents, either alone or in combination with immunotherapies, are being evaluated in various solid tumors including NSCLC, with promising activity in some cases, however, datopotamab deruxtecan recently failed to show a statistically significant OS improvement in phase III TROPION-Lung01 study.^555^ Given that ALK fusions are not membrane-bound proteins, the development of direct anti-ALK ADCs is indeed not feasible. However, the targeting of ADCs is not as perfect as anticipated, with only 1% reaching the tumor tissue. Furthermore, the ADCs did not significantly increase MTD of the associated non-conjugated drugs,^556^ which in clinical practice, eventuated their dosages often require to be adjusted to ensure safety – thereby classified as cytotoxic drugs herein.

Notably, combining chemotherapy, especially ADCs and immunotherapy can lead to overlapping or unexpected adverse effects^557,558^ and excessive chemotherapy can disrupt proliferating T_eff_ lymphocytes and gut microbiome balance, resulting in overall immune suppression. Consequently, caution is advised when employing this strategy, particularly through methods such as fractionated dosing or sequential administration.^559^

#### Radiotherapy

In the past two decades, significant progressions have been made in radiotherapy (RT) technologies, enabling accurate temporal and spatial delivery of radiotherapy. Radiotherapy also induces ICD, triggering DNA and RNA sensing cascades by various damage-associated molecular patterns (DAMPs) and upregulating large quantities of immunostimulatory or chemokines that ultimately activate the immune system. Moreover, the AEs associated with radiotherapy typically do not overlap with those related to immunotherapies,^560^ theoretically supporting the combination of ICIs with various modes of radiotherapy. However, not only is abscopal effect extremely rare, but radiotherapy does not enhance the response to the durvalumab plus tremelimumab combination therapy^561^ in patients with anti-PD-1-resistant NSCLC, despite ICIs being able to bolster the effectiveness of radiotherapy.^229^ Currently, the purpose of radiotherapy is evolving from its primary role in cancer ablation to an immunomodulatory drug that primarily aims to enhance the efficacy of ICIs, particularly for late-stage NSCLC treated with low-dose or hypofractionated radiotherapy.^561^ Moreover, when it comes to radiotherapy in conjunction with immunotherapy, achieving a balance between safeguarding lymph nodes – critical sites for the primary immune response involving T cells – and preventing local recurrence becomes paramount. Furthermore, it is well-established that circulating lymphocytes are more sensitive to radiation than resident tissue cells, resulting in more pronounced reductions and slower clonal recovery with conventional fractionated radiotherapy compared to SBRT. Particle radiation therapy, such as proton and carbon-based beams,^562^ as well as FLASH radiation therapy,^560^ can induce immunological features including upregulation of cell surface MHC molecules, intercellular adhesion molecule 1 (ICAM1), tumor associated antigens, and DAMPs. However, current clinical data does not provide sufficient evidence to fully substantiate these observations.^563^ A new therapeutic approach is electrofield treatment of tumors, which has been demonstrated to have promising activity as second-line therapy,^564^ despite awaiting further outcomes.

In summary, the radiation-induced immune effects are characterized as dynamic processes^565^ heavily influenced by tumor subtypes, drug features, radiation dose and fractionation, and the sequence of combining radiotherapy with immunotherapy.^560,566^ Therefore, determining the optimal regimen for combination in the treatment of NSCLC still faces significant challenges and requires extensive further research.

#### Targeted therapy

For NSCLC with driver gene mutations, strategies to circumvent resistance mechanisms involve alternating different generations or pathways of TKIs, like D3S-001,^567^ opnurasib, IBI351, glecirasib and RMC-7977^479^ targeting KRAS^G12C^, or zongertinib targeting HER-2,^113^ together with combinations across multiple pathways, such as EGFR inhibitors in conjunction with MET inhibitors,^568^ RET inhibitors, MEK inhibitors or SHP2 inhibitors. Alternatively, novel next-generation TKIs currently are undergoing early-stage clinical trials, including fourth-generation EGFR TKIs like EAI045, BLU-945^569^ and BLU-701, however, no phase III clinical trials have been conducted yet (Table 4). The insufficient inhibition strength of the drug against the C797S mutation and a reduced dependence of cancer cell survival on the C797S mutation are some putative challenges that may slow the progress of clinical studies. Another challenge is that the specific subgroup of patients (5–7%) is relatively small, leading to difficulties in patient enrollment. Of course, besides fourth-generation EGFR TKIs, other TKIs under development that target different mutations also face similar issues,^570^ like poziotinib, zipalertinib, BEBT-109, or afatinib combined with cetuximab^571^ targeting EGFR ex20ins,^572^ ningetinib targeting MET and AXL mutations,^573^ dostarlimab targeting homologous recombination repair-deficient (HRD),^574^ pevonedistat targeting NAE,^575^ pyrotinib targeting HER-2 mutations,^576^ bemcentinib targeting AXL,^577^ abemaciclib targeting CDK4/6, and tepotinib targeting MET exon 14-skipping or amplification.^323^ Similarly, for resistance associated with ALK mutations, TPX-0131 targeting ALK^G1202R^ and ALK^L1196M^ co-mutations, and NVL-655, a highly selective, CNS-penetrating next-generation TKI, targeting G1202R and L1196M mutations, as well as G1202R-L1198F and G1202R-G1269A co-mutations,^578^ all display promising activity. Therefore, further exploring new mechanisms of action that offer advantages over existing treatments is an urgent task.

**Table 4:** Fourth-generation EGFR TKIs under clinical investigation

Inhibitors	NCT numbers	Sponsors	Status
BLU-945	NCT04862780	Blueprint Medicines Corporation	Phase I/II
H002	NCT05552781	RedCloud Bio	Phase I/IIa
JIN-A02	NCT05394831	J INTS Bio	PhaseI/II
BPI-361175	NCT05329298	Betta Pharmaceuticals	Phase I/II
WJ13404	NCT05662670	Suzhou Junjing BioSciences	Phase I/II
QLH11811	NCT05555212	Qilu Pharmaceutical	Phase I
TQB3804	NCT04128085	Chia Tai Tianqing Pharmaceutical Group	Phase I
BBT-207	NCT05920135	Bridge Biotherapeutics, Inc.	PhaseI/II
BDTX-1535	NCT05256290	Black Diamond Therapeutics, Inc.	Phase I/II
HS-10375	NCT05435248	Jiangsu Hansoh Pharmaceutical	PhaseI/II
TAS3351	NCT05765734	Taiho Oncology, Inc.	PhaseI/II
WSD0922-FU	NCT06631989	Wayshine Biopharm, Inc.	Phase I/II

Targeted therapies for cancer not only exert direct antitumor effects but also exhibit certain immunomodulatory activities, such as T cell exhaustion, activation of T_reg_ cells and MDSCs, and impaired IFN-γ signaling and upregulated expression of immune checkpoint molecules.^501^ Moreover, certain oncogenic mutations in genes such as KRAS, BRAF, and PIK3CA are associated with high levels of pro-inflammatory cytokines and chemokines, as well as T-cell infiltration. This supports the rationale for many (though not all) concurrent or sequential combination approaches that integrate targeted anticancer drugs with immunotherapies,^44,579,580^ exemplified as ongoing studies where capmatinib, a MET inhibitor paired with nivolumab, and nimotuzumab with pembrolizumab and chemotherapy are being investigated for the second-line treatment of advanced NSCLC.

### Indirectly targeting cancer cells – anti-angiogenic drugs

Aberrant tumor angiogenesis within the TME not only facilitates tumor growth but also leads to treatment resistance.^581^ Consequently, the combinations of anlotinib with icotinib and ramucirumab with erlotinib for EGFR mutation,^582^ are currently under investigation.

For the immune system’s lens, anti-angiogenic drugs not only enhance drug delivery but also boost T-cell infiltration within tumors,^583^ facilitate polarization of TAMs towards a pro-inflammatory M1 phenotype, promote DC maturation, and activate effector T cells. They also inhibit T_reg_ cells, and myeloid cells, as well as downregulate PD-L1 expression in malignant cells.^583^ However, the development of immunotherapy combined with anti-VEGF agents in first-line NSCLC treatment has not been successful so far,^584^ regardless of high PD-L1 expression^585^ or high TMB. Owing to the distinctive low toxicity associated with anti-angiogenic therapy, coupled with the gradual drop in the development of new PD-L1/PD-1 inhibitors, as a second-line immunotherapies combined with anti-angiogenic treatments could potentially receive more focus, despite the results remaining equivocal. Atezolizumab in combination with bevacizumab demonstrates activity in the second-line treatment of non-squamous NSCLC, potentially overcoming resistance to ICIs.^586^ In PD-L1 positive advanced NSCLC, ramucirumab in conjunction with pembrolizumab exhibited controlled safety, with a median OS rate of 14.5 months, compared to 11.6 months with chemotherapy.^587^ Similarly, the combination of nintedanib with docetaxel serves as an effective second-line treatment strategy, showing a median PFS of 4.8 months in patients refractory to initial treatment with pembrolizumab plus chemotherapy. For patients with advanced NSCLC who have previously been treated with EGFR-TKIs or other targeted therapies, atezolizumab, pembrolizumab,^587^ or nivolumab in combination with VEGF inhibitors such as ramucirumab,^588^ have shown comparable efficacies. Additionally, anti-angiogenic multi-kinase inhibitors in combination with immunotherapy, for instance, anlotinib combined with toripalimab, camrelizumab, sintilimab,^589^ benmelstobart^590^ or TQB-2450, apatinib with camrelizumab and chemotherapy, lenvatinib with pembrolizumab,^591^ cabozantinib with atezolizumab and sitravatinib with nivolumab^592^ have all been investigated in clinical trials, revealing unequivocal safety profiles and promising or disappointing therapeutic benefits. In addition, retrospective analyses do support the use of anlotinib in combination with ICIs as second-line or later-line treatments.

To summarize, for frail patients intolerant to chemotherapy or receiving later-line treatment, the combination of immunotherapy with anti-angiogenic therapy remains a suitable option,^593^ although this approach still requires validation through large cohort studies.^364,594,595^ However, caution is warranted, given the risk of occasionally serious adverse events with multi-targeted kinase inhibitors, which can be particularly hard to distinguish from those coinciding and overlapping with ICIs.^581,596^ Currently, beyond at least three angiogenesis inhibitors approved in NSCLC, other targeted therapies against CD105, angiopoietin 1/2, and the tyrosine kinase receptor TIE2 (ANG1 receptor) are also undergoing clinical trials.

### Targeting the cell state of cancer cells (phenotypic plasticity)

While treatment-induced resistance is traditionally perceived through a genomic variation-centric viewpoint, providing clonal advantages to cancer cells,^597^ accumulating evidence emphasizes diverse non-mutation-based resistant states^598^ – where cancer cells can switch between different cellular lineage states without genetic alterations, a phenomenon known as cell plasticity. States of cell plasticity also manifest in EMT, facilitating the shedding and dissemination of tumor cells, or MET, adapting to organ colonization. Additionally, cancer cells can acquire stem cell features through dedifferentiation or metaplasia, or enter a dormant or DTP state.^599^

While the exact mechanisms underlying cellular plasticity are still uncertain, diverse processes probably involving epigenetic and transcriptional reprogramming,^600^ such as transcription factors like SOX2^597^ and chromatin regulators, notably like polycomb repressive complex 2 (PRC2), along with JAK/STAT inflammatory signaling pathways,^601^ may play central roles.

Consequently, various therapeutic approaches targeting the plasticity of cancer cells have been proposed. One strategy involves combining existing treatment regimens with different classes of epigenetic inhibitors, such as small molecule inhibitors against EZH2 or HDAC,^602^ alongside ICIs, all of which have shown encouraging outcomes even in lung cancer patients, with potential to eliminate slowly proliferating DTP cells. Furthermore, inhibiting TGF-β signaling pathway in combination with PD-L1 bispecific antibodies can also lead to the elimination of DTP cells.^603^ Given that autophagy also facilitates the formation of DTP, preclinical studies have uncovered that simultaneously inhibiting autophagy in NSCLC cells can potentiate antitumor responses of ICIs. Secondly, targeting the self-renewal capacity or promoting differentiation of CSCs, such as through Notch, WNT/β-catenin, and LGR5 pathway inhibitors, is being evaluated either alone or in combination with ICIs.^604^ Thirdly, since SNAI1, SNAI2, Twist1, ZEB1, and ZEB2, as well as CD70,^605^ FOXC2, SOX4, and PRRX1 are considered core transcription factors of EMT, their signaling pathway inhibitors are presently under evaluation alone or in conjunction with PD-1/PD-L1 inhibitors for NSCLC. Lastly, reawakening dormant cell subpopulations to enhance their sensitivity to treatment holds attractive prospects.

Unfortunately, current therapies targeting cellular lineage plasticity have not proven successful, likely due to overlapping phenotypes and regulatory mechanisms across different cell states attributed to bidirectional proliferation, metabolic flexibility, and environmental adaptation.^606^ Moreover, which tumors undergo this process of therapy-induced reprogramming and which molecular components are involved still remain unclear.

### Directly targeting immunocytes

Targeting the fundamental T cell processes of recruitment, infiltration, polarization, differentiation, activation, and proliferation – the basis of immunotherapies^501^ – to overcome drug resistance, is the most appealing direction, as this approach promises to induce longer-lasting reactions with fewer side effects and broader applicability, compared to chemotherapy, radiation therapy, and targeted treatments.^607^ Nevertheless, this represents a complex strategy due to the dynamic changes in functions and the presence of various subtypes and phenotypes of immune cells within the immunomicroenvironment

#### Modulating the immunosuppressive milieu

##### Immunotherapy through ICIs

LAG3, identified in 1990 as a homolog of CD4, acts as a negative regulator of CD4^+^T cell activation. Relatlimab, the first monoclonal antibody targeting LAG-3 approved by the FDA, showed improved PFS in a phase II-III trial for melanoma compared to nivolumab monotherapy,^608^ but its efficacy was less evident in NSCLC. Other anti-LAG-3 antibodies, such as favezelimab, Ieramilimab,^609^ fianlimab, Sym022, BI 754111, HLX26, TSR-033, INCAGN02385, REGN3767, and IMP321, are being evaluated for their effectiveness alone or in combination with other ICIs^610^ even in the neoadjuvant setting, however, on the overall, their activity has been moderate (Table 5).

**Table 5:** Ongoing phase II-III clinical trials evaluating immunotherapy-based combination strategies for advanced-stage NSCLC

Lines of therapy	Trial names	Assessed agents or regimes	Trial interventions	Special stratification criteria	Primary endpoints
First-line therapy	ACHIEVE Phase III(NCT06096844)	Pembrolizumab + Chemotherapy	Pembrolizumab + Chemotherapy vs. Pembrolizumab	≥70 years old, PD-L1 of 1–49%	OS
	QUILT 2.023 Phase III(NCT03520686)	Anktiva (IL-15 superagonist)	Cohort A: Anktiva + Pembrolizumab vs. Pembrolizumab Cohort B: Anktiva + Chemotherapy + Pembrolizumab vs. Chemotherapy + Pembrolizumab (squamous carcinoma) Cohort C: Anktiva + Chemotherapy + Pembrolizumab vs. Chemotherapy + Pembrolizumab (adenocarcinoma) Cohort D: Anktiva + Chemotherapy + Ipilimumab+ Nivolumab	PD-L1 ≥ 1% in Cohort A only	PFS
	INSIGNA Phase III (NCT03793179)	Pembrolizumab + Chemotherapy	Arm A: Pembrolizumab up to 2 years, if PD, followed by 4 cycles of Chemotherapy and maintenance of Pemetrexed Arm B: Pembrolizumab up to 2 years, if PD, followed by 4 cycles of Chemotherapy and maintenance of Pemetrexed+ Pembrolizumab Arm C: Pembrolizumab+ Chemotherapy for 4 cycles and maintenance of Pembrolizumab for 2 years if without PD	PD-L1 ≥ 1% Adenocarcinoma	OS
	ELDERLY Phase III(NCT03977194)	Atezolizumab+ Chemotherapy (1 cycle/four weeks)	Atezolizumab + Chemotherapy vs. Chemotherapy	70–89 years old, regardless of PD-L1 levels	OS
	Cabatezo-1 Phase II(NCT05859217)	Cabozantinib + Atezolizumab	Cabozantinib + Atezolizumab	PD-L1 < 1%	ORR
	Phase Ib/II(NCT06162572)	S095018 (anti-TIM3 antibody) S095024 (anti-CD73 antibody) S095029 (anti-NKG2A antibody)	Cemiplimab + S095018 or Cemiplimab + S095024 or Cemiplimab + S095029 vs. Cemiplimab	PD-L1 ≥ 50%	DLT AEs ORR
	EMPOWERVAX Lung1 Phase II (NCT05557591)	BNT116 (Vaccine) + Cemiplimab	BNT116 + Cemiplimab vs. Cemiplimab	PD-L1 ≥ 50%	ORR
	GALAXIES LUNG-201 Phase II (NCT05565378)	Belrestotug (anti-TIGIT antivody) Dostarlimab (anti-PD-1 antibody) Nelistotug (anti-CD96 antibody)	Belrestotug + Dostarlimab vs. Belrestotug + Dostarlimab + Nelistotug vs. Dostarlimab vs. Pembrolizumab	PD-L1 ≥ 50%	ORR
	KEYNOTE-C62 Phase II (NCT05860296)	SLC-391 (AXL inhibitor)	SLC-391+ Pembrolizumab	PD-L1 ≥ 1%	AEs ORR
	Phase IINCT04495153	CAN-2409 (oncolytic virus)	CAN-2409+ ICIs		DCR ORR AEs
	KETNOTE-B36 Phase II (NCT04892472)	NovoTTF-200T (tumor treating fields with 150 kHz) + Pembrolizumab	NovoTTF-200T + Pembrolizumab vs. Pembrolizumab	PD-L1 ≥ 1%	PFS
	CA224-104 Phase II NCT04623775	Relatlimab + Nivolumab + Chemotherapy	Relatlimab + Nivolumab + Chemotherapy vs. Nivolumab + Chemotherapy	Regardless of PD-L1 levels	ORR AEs
	SAVIMMUE Phase II (NCT04108026)	Durvalumab	Durvalumab	PS = 2 or 3 PD-L1 ≥ 25%	Grade 3–5 AEs
	NIVIPI-Brain Phase II (NCT05012254)	Nivolumab + Ipilimumab + Chemotherapy	Nivolumab + Ipilimumab + Chemotherapy	Brain metastases	Rate of intracranial clinical benefit
	ARC-7 Phase II (NCT04262856)	Zimberelimab (anti-PD-1 antibody) Domvanalimab (anti-TIGIT antibody) Etrumadenant (A2aR and A2bR antagonist)	Zimberelimab vs. Domvanalimab + Zimberelimab vs. Domvanalimab + Zimberelimab + Etrumadenant	PD-L1 ≥ 50%	ORR PFS
	Phase I/II (NCT03377023)	Nivolumab + Ipilimumab+ Nintedanib	Nivolumab + Ipilimumab + Nintedanib	Regardless of PD-L1 levels	ORR
	DIAL Phase II/III (NCT05255302)	Pembrolizumab as a maintenance therapy	Pembrolizumab+ Chemotherapy for 6 months followed by Pembrolizumab vs. Chemotherapy or Observation as a maintenance therapy	Regardless of PD-L1 levels	OS
	Phase II (NCT04856176)	Sargramostim (GM-CSF) + Pembrolizumab	Sargramostim + Pembrolizumab +/- Pemetrexed	PD-L1 range of 1-49%	PFS OS
	EDGE-Lung Phase II (NCT05676931)	Domvanalimab (anti-TIGIT antibody) Zimberelimab (anti-PD-1 antibody) Quemliclustat (CD73 inhibitor)	Domvanalimab + Zimberelimab vs. Quemliclustat + Zimberelimab vs. Quemliclustat + Zimberelimab + Chemotherapy vs. Quemliclustat + Zimberelimab + Chemotherapy vs. Domvanalimab + Quemliclustat + Zimberelimab + Chemotherapy		ORR AEs
	CITISCAPE Phase II (NCT03563716)	Tiragolumab (anti-TIGIT antibody) + Atezolizumab	Tiragolumab + Atezolizumab vs. Placebo + Atezolizumab	PD-L1 ≥ 1%	ORR PFS
	EDEN Phase III (NCT03542461)	Nivolumab	First-line standard treatment followed by Early switch nivolumab maintenance vs. Delayed second-line nivolumab	Regardless of PD-L1 levels	OS
	Phase II (NCT06403111)	fecal microbiota transplantation (FMT)	FMT + Tislelizumab + Chemotherapy	PD-L1 < 50%	PFS
	Phase II (NCT06554613)	Olanzapine	Olanzapine + Nivolumab ± Chemotherapy vs. Nivolumab ± Chemotherapy		OS
	IMMONC0008 Phase II (NCT06322108)	Botensilimab (anti-CTLA-4 antibody) Balstilimab (anti-PD-1 antibody)	Botensilimab + Balstilimab	Regardless of PD-L1 levels	PFS
	Phase II (NCT04937972)	SHR-1701 (anti-PD-L1/TGF- β antibody)	SHR-1701+ Fluzoparib as maintenance therapy for chemo-immunotherapy	Regardless of PD-L1 levels	PFS
	KIOM-NSCLC-ICT-01 PhaseII(NCT06162572)	Bojungikki-tang (BJIKT, herbal medicine)	BJIKT + Pembrolizumab vs. Pembrolizumab	PD-L1 ≥ 50%	PFS
	BNT327-06 Phase II/III (NCT06249854)	BNT327 (anti-PD-L1/VEGF antibody)	BNT327+ Chemotherapy vs. Pembrolizumab + Chemotherapy	Regardless of PD-L1 levels	PFS OS
Subsequent-line therapy	HUDSON Phase II (NCT03334617)	Durvalumab Olaparib AZD9150 (STAT3 inhibitor) AZD6738 (ART inhibitor) Vistusertib (mTOR inhibitor) Trastuzumab deruxtecan Cediranib (multi-kinase inhibitor)	Durvalumab + Olaparib vs. Durvalumab + AZD9150 vs. Durvalumab + AZD6738 vs. Durvalumab + Vistusertib vs. Durvalumab + trastuzumab deruxtecan vs. Durvalumab + Cediranib	Resistance to ICIs and chemotherapy	ORR
	Phase I/II(NCT03581487)	Durvalumab Tremelimumab Selumetinib (MEK inhibitor)	Durvalumab + Tremelimumab + Selumetinib	Resistance to ICIs and chemotherapy	MTD PFS
	BAH2472 Phase I/II (NCT06538012)	Lifileucel (TILs)	Lifileucel + Pembrolizumab	No standard treatments available	AEs ORR
	Landscape 1011 Phase Ib/II (NCT04585815)	Sasanlimab (anti-PD-1 antibody) Encrafenib (BRAF inhibitor) Binimetinib (MEK inhibitor) Axitinib (a multi-kinase inhibitor) SEA-TGT (anti-TIGIT antibody)	Sub-study A: Sasanlimab + Encrafenib + Binimetinib Sub-study B: Sasanlimab + Axitinib+SEA-TGT	BRAF V600E mutation for sub-study A PD-L1 ≥ 1% for sub-study B	ORR
	Phase II (NCT03971474)	Ramucirumab+ Pembrolizumab	Ramucirumab + Pembrolizumab vs. Standard of care therapy	Resistance to ICIs	OS
	LEAP-008 Phase III (NCT03976375)	Pembrolizumab+ Lenvatinib	Pembrolizumab + Lenvatinib vs. Docetaxel vs. Lenvatinib	Resistance to ICIs and chemotherapy	OS
	Phase II/III (NCT05096663)	Anktiva+ Pembrolizumab	Anktiva + Pembrolizumab vs. Standard of care therapy	Resistance to ICIs	OS
	KEYPEMLS-004 Phase II (NCT05599789)	Pembrolizumab Plinabulin Docetaxel	Pembrolizumab + Plinabulin + Docetaxel	Resistance to ICIs	ORR
	MILES-5 Phase II (NCT03975114)	Tremelimumab Durvalumab Chemotherapy drugs	Tremelimumab + Durvalumab + Chemotherapy vs. Durvalumab + Chemotherapy vs. Chemotherapy	≥70 years old	12-month OS rates
	COMBI-TED (NCT04884282) Phase II	Tedopi (vaccine)	Tedopi + Docetaxel vs. Tedopi + Nivolumab vs. Docetaxel	Resistance to ICIs and chemotherapy	1-year survival rates
	Phase II (NCT03965689)	Pevonedistat (NAE inhibitor)	Pevonedistat + Carboplatin + Paclitaxel	Resistance to previous ICI treatment	ORR
	LOTOS Phase II (NCT05941897)	Ceralasertib (ATR inhibitor)	Ceralasertib + Durvalumab	Resistance to ICIs and chemotherapy	ORR
	KEYVIBE-002 Phase II (NCT04725188)	MK-7684A (Pembrolizumab/Vibostolimab coformulation)	Arm A: MK-7684A Arm B: MK-7684A + Docetaxel Arm C: Docetaxel	Resistance to ICIs and chemotherapy	PFS
	STUDY-21-00907 Phase I/II (NCT05013450)	Dupilumab (anti IL-4Rα antibody) Anakinra (anti IL-1Rα antagonist)	Dupilumab + Anakinra+ anti-PD-1/PD-L1 vs. Dupilumab + anti-PD-1/PD-L1	Resistance to ICIs	ORR
	Pragmatica-Lung Phase III(NCT05633602)	Ramucirumab + Pembrolizumab	Ramucirumab + Pembrolizumab vs. Standard of care chemotherapy	Resistance to ICIs and (or) chemotherapy	OS
	Phase II/III NCT06616584	Cemiplimab Docetaxel Ramucirumab	Cemiplimab + Docetaxel + Ramucirumab vs. Docetaxel + Ramucirumab	Resistance to ICIs and (or) chemotherapy	OS
	CAPTRAL2024v1 Phase II (NCT06388031)	Pembrolizumab Tisleizumab Camrelizumab Toripalimab	ICIs rechallenge in long-term responders to prior ICIs	Resistance to ICIs	PFS
	VIRO-25 Phase II (NCT06463665)	Olvi-Vec (oncolytic vaccinia virus)	Olvi-Vec + Chemotherapy + anti-PD-1/PD-L1 antibody vs. Docetaxel	Resistance to ICIs	PFS
	QUILT-3.055 Phase II (NCT03228667)	Anktiva PD-L1 t-haNK (PD-L1-targeted high-affinity NK cells)	Anktiva + Pembrolizumab Anktiva + Nivolumab Anktiva + Atezolizumab Anktiva + Avelumab Anktiva + Durvalumab Anktiva + Pembrolizumab + PD-L1 t-haNK Anktiva + Nivolumab+ PD-L1 t-haNK Anktiva + Atezolizumab+ PD-L1 t-haNK Anktiva + Avelumab+ PD-L1 t-haNK Anktiva + Durvalumab+ PD-L1 t-haNK	Solid tumor patients with resistance to ICIs	ORR
	ATHENA Phase II (NCT04322617)	Anlotinib + ICIs	Same ICIs rechallenge in combination with Anlotinib New ICIs rechallenge in combination with Anlotinib	Resistance to ICIs and chemotherapy	PFS
	Phase II (NCT05738317)	Adebrelimab (PD-L1 inhibitor)	Adebrelimab + Bevacizumab + Albumin Paclitaxel	Resistance to ICIs	PFS
	Phase II (NCT06467500)	Cadonilimab	Cadonilimab + Chemotherapy	Resistance to ICIs	ORR

We excluded trials where the study objectives were unclear, at least as far as we understood them

*PFS* progression free survival, *AEs* adverse events, *OS* overall survival, *ORR* objective response rate, *ECOG* eastern Cooperative Oncology Group, *NAE* NEDD8-activating enzyme, *IL-15* Interleukin-15

A promising avenue of research is TIGIT (T-cell Ig and ITIM domain), which was first discovered in 2009. TIGIT can competitively bind to the ligand of the activated CD226 receptor, and co-express with PD-1. Consequently, dual inhibition of TIGIT and PD-1/PD-L1 is a promising development pipeline,^611^ as exemplified by the combination therapy of the TIGIT inhibitor tiragolumab and atezolizumab in phase II studies (CITYSCAPE), which showed improved ORR and PFS, though the OS results were yet to be released.^612^ Similarly, vibostolimab, used either alone or in combination with pembrolizumab, demonstrated an ORR of 26% in NSCLC.^613^ The ongoing phase III SKYSCRAPER-06 study is evaluating the combined use of tiragolumab and atezolizumab with chemotherapy, while the SKYSCRAPER-03 trial is assessing atezolizumab and tiragolumab specifically for unresectable stage III NSCLC. Other TIGIT-targeting agents, such as domvanalimab, ociperlimab, M6223, BMS-986207, IBI939, etigilimab, rilvegostomig, or BAT6021, when used in combination with various PD-1/PD-L1 drugs or even chemotherapy, have exhibited promising short-term efficacy and safety profiles, however, their long-term prognosis remains to be substantiated by additional data.^610^

T cell immunoglobulin and mucin domain 3 (TIM-3) identified in 2002, is another inhibitory immune checkpoint molecule that is highly expressed in dysfunctional T cells. In patients with NSCLC, the combination therapy of TIM-3 antibody (sabatolimab,also called MBG453) and PD-1 inhibitor (spartalizumab) has shown antitumor activity.^614^ Various combinations of TIM-3 antibodies with PD-1 antibodies (dostarlimab and nivolumab) or other drugs are currently being explored.^610^

VISTA (V-domain Ig suppressor of T cell activation), a transmembrane protein discovered in 2011,^615^ functions by binding to VSIG-3, thereby inhibiting T-cell proliferation and cytokine production. Drugs targeting VISTA, such as CI 8993, HMBD-002, and SNS-101, have been developed and are currently undergoing evaluation in early clinical trials.

B7 homolog 3 (B7-H3, also known as CD276), discovered in 2001 as part of the B7 family,^616^ facilitates immune evasion by impeding T cell infiltration and promoting exhaustion of CD8^+^ T cells. Antibodies against B7-H3, such as T-1A5, 8H9, and enoblituzumab (MGA271 or TJ271), have been developed, however, their clinical efficacy has been limited. Notably, a combination treatment with enoblituzumab and pembrolizumab has shown objective responses in 5 out of 14 NSCLC patients.^617^

B and T lymphocyte agonist (BTLA), a member of the TNF receptor family, was discovered in 2003, and herpevirus entry mediator (HVEM) (also known as TNFRSF14) was identified in 1996. Currently, the BTLA/HVEM axis represents a promising new target in cancer immunotherapy. The anti-BTLA blocking antibody, tifcemalimab (icatolimab), has shown promising preliminary efficacy and safety profile in various phase I clinical trials as a monotherapy or in combination with other therapies, including ICIs, chemotherapy, and cell therapy, in PD-1/PD-L1 refractory NSCLC. Nevertheless, BTLA can supply either co-stimulatory or co-inhibitory signals to activated CD8^+^T cells.^618^ Therefore, deep and dynamic functional characterization of the BTLA/HVEM axis in diverse contextual settings is crucial for ensuring optimal clinical outcomes.

Other inhibitors targeting the colony-stimulating factor 1 receptor tyrosine kinase (CSF-1R) discovered in 1987 (such as emactuzumab),^619^ NKG2A and CD39/CD73, along with mCCR5-Ig fusion proteins, as well as CXCR2 antagonists in combination with ICIs are being assessed. However, IL-1β blocking canakinumab in combination with pembrolizumab and chemotherapy did not extend PFS or OS in NSCLC.^620^

##### Beyond single-target antibodies

Developments in antibody engineering over the past three decades have enabled specificity towards multiple distinct antigens or different epitopes of the same antigen, enhancing the therapeutic efficacy of antibodies.^621^ While immunotherapies, including the combination therapy of ipilimumab and nivolumab, have seen significant success in several cancers and recently might mitigate resistance to PD-(L)1 inhibition in NSCLC with STK11 and/or KEAP1 alterations,^113^ the associated immunological adverse reactions remain a substantial barrier. Bispecific antibodies provide one feasible strategy to address this challenge, leveraging shared Fc regions to mitigate Fc-mediated toxicity,^622^ although enhanced FcγR affinity can harness FcγR-dependent mechanisms to potentiate T cell responsiveness, reduce intratumoral T_reg_s, and enhance antigen-presenting cell activation.^623^ An additional potential advantage of bispecific antibodies lies in their ability to concurrently block CTLA-4 and PD-1, offering higher specificity binding.^624^ Consequently, in the past decade, there has been significant interest and focus on the development of these molecules, as demonstrated by the culmination of the approvals of 18 bispecific antibodies by early 2025, with 14 of them designated for cancer therapy.^621^

Various PD-1 + CTLA-4 dual blockade inhibitors are currently under development, such as QL1706 (PSB205), MGD019, AK104 (also known as cadonilimab, with approval of cervical cancer as indication in China), which have shown promising efficacy and safety in NSCLC patients, and awaiting the maturation of data from phase II and III randomized trials. KN046, which simultaneously inhibits the PD-L1 and CTLA-4 pathway, has displayed good efficacy and safety as a the second-line treatment of advanced NSCLC, however, LY3415244, a bispecific antibody targeting TIM-3 and PD-L1, was prematurely terminated due to unexpected allergic reactions.^625^ Other drugs, targeting PD-1 + PD-L1, PD-1/PD-L1 + CTLA-4, PD-1/PD-L1 + LAG-3, CTLA-4 + LAG-3, TIGIT + PD-1, PD-1 + TIM-3, TGF-β + PD-L1^253,626^ and PD-1 + VEGF,^627^ are undergoing clinical trials to evaluate their efficacy as monotherapies or in combination with chemotherapy. As noted, while multi-specific antibodies show promise, they exhibit varying affinities for each binding site, thus, fine-tuning of these affinities for each binding site is required to optimize the pharmacokinetic properties for effectively blocking two immune checkpoints.^628^

#### Upregulation of immune activation pathways

T cell activation requires co-stimulatory molecules primarily belonging to the tumor necrosis factor (TNF) superfamily. Agonizts targeting 4-1BB, OX40, CD27, GITR, and ICOS are being developed in clinical trials either alone or in combination with other co-inhibitory monoclonal antibodies to enhance T-cell mediated antitumor immunity.

4-1BB was first reported in 1989 and has the potential to enhance various T cell functions, increase cytokine production, potentiate memory differentiation, and reverse T cell dysfunction/exhaustion states. Agonizts that activate 4-1BB have been available for over 25 years, but they only recently gained significant attention, with at least 20 4-1BB agonizts currently in development. Considering the hepatotoxicity, urelumab, a 4-1BB agonist, has been reintroduced into clinical trials at a tolerable low-dose scheme, but with a response rate of only 13%. Moreover, combinations of new 4-1BB activators with PD-1 pathway inhibitors, such as urelumab+ nivolumab or utomilumab+ pembrolizumab, have demonstrated therapeutic activity and favorable liver safety profiles, with some cases even achieving CR.^629^ Genmab, a PD-L1 and 4-1BB bispecific antibody (GEN1046)^630^ and GEN1042, a CD40 and 4-1BB bispecific antibody^631^ have exhibited single-agent therapeutic activity in tumors resistant to anti-PD-L1 antibodies. Furthermore, to minimize off-target effects and toxicity, specifically designed bispecific antibodies combining 4-1BB with tumor antigens (such as HER-2, EGFR, or CEACAM5) can selectively activate the 4-1BB signaling pathway within the TME.

OX-40 was discovered in 1987 and transiently expressed after T cell activation, where its interaction with OX-40L enhances T cell survival and memory formation. The clinical efficacy of OX-40 agonist monotherapy has been disappointing, owing to limited clinical benefits being observed when used in combination with atezolizumab, pembrolizumab, nivolumab, and/or ipilimumab.^632^ Bispecific antibodies, such as ATOR-1015 targeting both CTLA-4 and OX-40, and FS120 targeting both 4-1BB and OX-40, are currently being evaluated in clinical trials for their safety and tolerability.

ICOS (Inducible co-stimulator) was first recognized in 1999 and facilitates the promotion of antitumor T cell responses once activated in Th1 and other T effector cells. However, activating this pathway in T_reg_s can promote tumor growth. Agonizts (such as JTX-2011 and feladilimab) or antagonists (such as MEDI-570 and KY1044) targeting this pathway are being studied as cancer immunotherapies alone, or combined with anti-CTLA4 or anti-PD-1/PD-L1 antibodies,^633^ and the agonist feladilimab (GSK3359609) has advanced to phase III clinical trials (NCT04128696).

First described in 1997, glucocorticoid-induced TNF-related receptor (GITR) has been shown to enhance the overall antitumor activity of T lymphocytes. Generally, the efficacy of GITR agonistic antibodies as monotherapies has been disappointing, including AMG-228, ragifilimab, BMS-986156, GWN323, INCAGN1876, MK-1248, MK-4166, REGN6569, and TRX518. Therefore, akin to the development paths of other drugs, combinations with already efficacious ICIs (such as pembrolizumab, ipilimumab, nivolumab, spartalizumab, or retifanlimab) as well as with radiotherapy and chemotherapy, and even bispecific antibodies (such as WO2018091739) are being explored.^634^

In 2009, research revealed that when activated, the stimulator of interferon genes (STING) triggers a downstream signaling cascade leading to the production and release of type I IFN and other pro-inflammatory cytokines, which activates innate immune cells and promotes their maturation, thereby triggering adaptive immune responses.^635^ In preclinical and early clinical studies, STING agonizts have been proven to be capable of eliciting significant systemic immune responses, and they are currently being assessed in combination with other ICIs. Notably, the activation of the cGAS-STING pathway can also be accomplished through alternative means, such as small molecule DNA repair inhibitors (PARP inhibitors), or by augmenting DNA damage through radiotherapy and chemotherapy.^636^

Across various preclinical tumor models, a range of targeted agents, such as CDK4/CDK6 and CDK7 inhibitors (palbociclib, ribociclib, and abemaciclib), the JAK1 inhibitor (itacitinib), BCL2 inhibitors such as venetoclax and navitoclax,^637^ the aminopeptidase inhibitor (ubenimex), PARP inhibitors (niraparib), and the selective anti-insulin growth factor 1 receptor antibody (dalotuzumab) or the intermediate-affinity cytokine binding to IL-2 receptor (nemvaleukin)^638^ and IL-4 fusion protein,^534^ have shown immunostimulatory effects and activity in NSCLC when combined with anti-PD-1 antibodies. However, these drugs have not yet been adopted for clinical use.

Overall, the efficacy of most agonizts, both individually and in combination, has been less than ideal, with challenges and obstacles including issues related to target affinity, antigen epitope selection, receptor occupancy, interactions and half-life of Fcγ receptors. There are also concerns regarding the toxicity of agonizts, such as the hepatotoxicity of 4-1BB agonizts. Lastly, and perhaps most importantly, many agonizts target multiple molecules in various immune cells, which can potentially exert different and even opposing functions, such as T_eff_ and T_reg_ cells. Moreover, the strength and amplitude of co-stimulatory signals are dynamic and vary across spatiotemporal dimensions. In addition to co-stimulatory agonizts, T-cell engagers are also being developed in NSCLC, targeting peptides such as survivin, among others,^639^ although currently lagging behind SCLC treated by BiTEs targeting DLL3. To enhance the antitumor efficacy and safety of agonizts, strategies are being developed, including the activation of antibodies through enzymatic activation or conformational changes dependent on pH, or the combination with existing inhibitory ICIs, vaccines, OVs, radiation therapy, chemotherapy, targeted therapy, and adoptive cellular therapy, while closely monitoring for immune side-effects.^409,640^

#### Cancer vaccines to upregulate immune activation signals

Cancer immunotherapy mostly revolves around the basic interaction between T cell receptors (TCR) and their specific antigens, hinting that vaccines are pivotal components thereof.^641^ Based on the origin of antigen components, cancer vaccines are classified into categories such as prepared whole tumor cells, MHC-specific peptides/proteins,^642^ recombinant proteins expressed in DCs using viral or bacterial vectors, various nucleic acid molecules (like RNA or DNA), as well as various adjuvant components that contain Toll-like receptor (TLR) agonizts to enhance immune responses against tumor-associated antigens (TAAs).

In recent years, there have been two significant advancements in cancer vaccines. Firstly, high-throughput sequencing techniques, combined with computational algorithms and machine learning tools, have been optimized to predict the affinity, antigenicity, and potency of more likely mutated epitopes recognized by T cells, and to design personalized vaccines for individual patient.^643^ Secondly, mRNA vaccines encoding tumor-related or tumor-specific antigens have been developed. These vaccines deliver engineered synthetic mRNA to autologous DCs or directly to cancer cells, enabling antigen expression, MHC presentation, and the activation cascade of both CD8^+^ and CD4^+^T cells, thereby inducing an antitumor response.^643^ The mRNA vaccine technology, garnering the Nobel Prize in Physiology or Medicine in 2023,^644^ has also multiple advantages. Clinical studies have employed dozens of mRNA vaccines – each containing 2–20 mutations, which are tailored to each patient. In NSCLC, at least 10 neoantigen-based tumor mRNA vaccines, like EGFR or ALK vaccines, are undergoing clinical trials,^645^ either as monotherapies or in combination with anti-PD-1 antibodies or other treatments, which preliminarily demonstrate their feasibility, immunogenicity, and safety.^646,647^

However, with the exception of sipuleucel-T (for prostate cancer), the FDA has not approved any therapeutic cancer vaccines, suggesting that there are still numerous challenges, including weak immunogenicity, off-target effects, and suppressive immune microenvironments. To overcome these challenges, a series of strategic approaches are being explored, such as employing varied bioinformatics techniques for the analysis of transcriptomics and proteomics to refine neoantigen optimization and selection, enhancing delivery systems through vaccine encapsulation in degradable-resistant hydrogels^648^ or reprogram tumor cells by adenoviral delivery of the transcription factors,^649^ and conducting preclinical testing in conjunction with other therapies^133^ – after all, monotherapy for advanced cancer remains a considerable challenge.

#### Oncolytic virus to upregulate immune activation signals

OVs constitute a novel class of antitumor drugs that not only directly lyse tumor cells but also induce ICD, which can facilitate T cell activation and infiltration as an in situ vaccine. To date, talimogene laherparepvec (T-VEC) and nadofaragene firadenovec have been approved by the FDA. Other OVs include H101 from China for nasopharyngeal carcinoma, and teserpaturev from Japan for glioblastoma.^650^

Primary OVs have limited oncolytic activity specifically against lung cancer, thus one direction for OVs is to harness their immunostimulatory, non-overlapping, and tolerable safety profiles to develop a higher-order combination with the other strategies. The combination of OVs with immunotherapies(OV-ICB) targeting CTLA4, PD-1, TIGIT, TIM3, and LAG3, and even with radiotherapy, for the treatment of advanced NSCLC is still in its initial stages^650^ and has not demonstrated superior efficacy compared to immunotherapies alone.^651^ Nonetheless, we are eagerly awaiting the outcomes of an ongoing clinical trial evaluating oncolytic coxsackievirus treatment for metastatic NSCLC.

To enhance the potency of OVs, multiple approaches to arm them have been developed. Initially, using the viral genome as a platform to express anti-PD-1 antibodies, BiTEs, or antibodies targeting other molecules can augment cancer cell killing and immunostimulation.^652^ Additionally, altering delivery strategies, such as intraperitoneal injection, can more evenly distribute the virus into larger treatment areas, as evidenced in our pleural effusion clinical trials.^653^ Moreover, oncolytic viruses can also be delivered to lung tumors in powder or aerosol form, which is especially suitable for patients with central lung cancer and related symptoms. Finally, OVs can be encapsulated in nanoparticles or liposomes to evade antibody clearance, achieving the goal of repeated systemic intravenous administration of OVs.^654^

#### Directly supplementing immune cells

The interaction between T cells and cancer cells within the TME, mirrors the predator-prey dynamics found in free-living species populations. Therefore, directly increasing the number of predators – through adoptive immune cell infusion therapy, including TILs, engineered T cell receptors (TCR) T cells, and chimeric antigen receptor (CAR) T cells – is a reasonable strategy.^655^

TIL therapy, originating in the 1980s, involves the isolation and expansion of T cells targeting tumor-associated antigens (TAAs) or neoantigens from lymphocytes obtained via tumor biopsies, then transfusing these cells into lymphodepleted patients This therapy was approved for use in melanoma in 2024.^656^ Recently, clinical trials evaluating the combination of autologous TIL^657^ and CIK cell therapy (Fig. 12) with anti-PD-1 therapy in metastatic NSCLC have shown favorable tolerability and preliminary efficacy, thus confirming clinical trials are being conducted for further validation.^658^

Similarly in the 1980s, researchers started investigating the technique of genetically modifying highly antigen-specific TCRs (T-cell receptors) and transferring them into naturally unprimed T cells for antitumor therapies. TCR T cells require matching with the patient’s HLA genotype (such as HLA-A*02:01), and can target multiple TAAs, including the melanoma antigen gene (MAGE) family and New York esophageal squamous cell carcinoma 1 (NY-ESO-1), as well as non-mutated overexpressed antigens such as carcinoembryonic antigen (CEA) and mesothelin or differentiation antigens like gp100 and MART-1.^659,660^ Currently, several phase I clinical trials are underway to assess the efficacy and safety of TCR T-cell therapy targeting a specific protein, NY-ESO-1, either as a monotherapy or in combination with pembrolizumab for advanced NSCLC.^661^ However, the high efficiency of TCR signaling has raised significant safety concerns, regarding the potential for TCR T cells to induce serious off-target toxicity in major organs, possibly due to cross-reactivity caused by shared epitopes. Fortunately, no such safety issues were observed in recent clinical studies targeting MAGE-A4 (recently approved by FDA for synovial sarcoma) and NY-ESO-1 with TCR T-cell therapy, and they are currently being explored for application in various solid cancers.^662^

At present, CAR-T cells signify a groundbreaking development within the realm of immunotherapy, leveraging modular protein components to redirect the responsiveness of immune cells towards specific targets. Following the success of six CAR-T cell products,^663^ various clinical trials are now exploring the utilization of CAR-T cell therapies for solid tumors, notably including NSCLC. For instance, clinical trials treating patients with EGFR-positive refractory/recurrent NSCLC have demonstrated that EGFR CAR-T cells are both feasible and safe. Other targets include CEA, orphan tyrosine kinase receptor ROR1, mesothelin, IL-13Rα2, and di-sulfide linked GD2, all of which have reported preliminary efficacy and safety in NSCLC. P-MUC1C-ALLO1, an allogeneic CAR-T cell therapy, has shown promising clinical outcomes, albeit with an overall response rate of only 9%. Combining CAR-T cells with PD-1 inhibitors might enhance the efficacy of CAR-T cell therapies, as evidenced in clinical studies such as SNK01 plus pembrolizumab for NSCLC treatment (2-year survival rate: 58.3% vs.16.7% for pembrolizumab alone), however, these results need to be validated.

The integration of CAR-T cells with ICIs holds promise for cancer therapy, yet a major concern is the toxicity associated with CAR-T cells, particularly on-target off-tumor toxicity (OTOT), which involves the recognition of targets outside the tumor and subsequent destruction of non-malignant tissues expressing the target antigen.^663^ To address these issues, various molecular structures can be used to create Boolean logic gates to control the activation of CAR-T cells, including “IF/THEN”, “AND”, “OR”, and “NOT” strategies. For instance, an AND logic circuit requires both target antigens to be positive for CAR-T cell activation, allowing for specific cytotoxicity against cells expressing both target antigens. However, the addition of an extra target antigen increases the risk of tumor cell escape. An alternative option involves creating reversible switches, whereby, in the absence of protease inhibitors such as asunaprevir, proteases cleave masking elements off the surface of CAR structures, enabling CAR recognition of the corresponding antigen and initiating downstream signaling pathways. A variety of condition-responsive modules, including ultrasound-sensitive Piezo1 ion channels, heat shock proteins,^664^ and modules sensitive to magnetic fields, X-rays, and electric fields,^665^ can be utilized for remote control of local CAR-T cell activation. Once exposed to an external stimulus, they trigger cascades of signaling reactions, thereby reducing the potential risks associated with OTOT Toxicity.

Several strategies have been employed to address another bottleneck – how to enhance the efficacy of CAR-T cell therapy. Firstly, reducing antigen escape can be achieved by engineering multi-specific CARs that bind to multiple distinct epitopes or antigens,^666^ or by inserting antigen ligands recognized by CAR into the tumor cell membrane to redirect CAR-T cells.^667^ Secondly, selecting stronger co-stimulatory domains is crucial, such as CD28 co-stimulatory CARs. However, higher affinity is not always beneficial, as excessively high-affinity CAR architectures may initially be very effective but can trigger cytokine release syndrome (CRS) and hasten T cell exhaustion or death. Thirdly, recent studies utilizing single-cell transcriptomics, epigenetics, and proteomics, and even TCR sequencing, have uncovered that the overexpression of the Prodh2 gene, which is involved in proline metabolism,^177^ along with the upregulation of IL-15 and CCR4 (receptors for several chemokines),^668^ the disruption of DNA methylation regulatory molecules such as TET2 and SUV39H1 and the knockout of the DNMT3A gene can collectively enhance T cell amplification, boost cell persistence, and thereby improve the overall antitumor response. Furthermore, inhibiting the PI3K-mTOR-AKT, BTK, or tyrosine kinase signaling pathways can be utilized to prevent CAR-T cells from exhaustion, and transform them into stem cell-like and central memory subpopulations. CAR-X also holds promise, leveraging antitumor activity of innate T cells, including natural killer cells,^669^ iNKT cells, γδ T cells, and macrophages.^670^ Lastly, bedside rapid manufacturing technologies and equipment such as Clinimacs Prodigy^671^ or off-the-shelf allogeneic CAR-T cells are under investigation, of which aim to reduce manufacturing costs and shorten treatment times, thereby expanding opportunities for patient treatment.

In essence, the above various strategies employed to enhance CAR-T therapies, as living drugs, should not operate independently but rather complementarily and in balance.

### Targeting metabolic pathways

The reprogramming of cellular metabolism and the ability to evade immunological destruction are now recognized as the new hallmarks of cancer, marking a significant advancement in our understanding of cancer mechanisms.^114^ Cancer cells exhibit highly active metabolic pathways that can lead to nutrient depletion and hypoxia within the TME, fostering a metabolic competition between tumor cells and contiguous immune cells.^672,673^

#### Glucose metabolism

Glucose metabolism is a multi-step process regulated by various enzymes, primarily including those involved in glycolysis, tricarboxylic acid cycle, and nucleotide synthesis, which govern intake, transport, breakdown, and synthesis of glucose. The mutations in IDH1 and IDH2,^674^ are common across various cancer types, instead being rare in NSCLC. IDH inhibitors, approved for the treatment of relapsed or refractory AML and cholangiocarcinoma, represent a success in targeted therapy against cancer metabolism. Currently, a phase II clinical trial is recruiting participants to evaluate the efficacy of ivosidenib in combination with nivolumab for treating metastatic solid tumors including NSCLC. Other inhibitors targeting different sites, including but not limited to MEDI7247, 2-DG, CPI-613, STF-31, WZB117, ritonavir, CO-101 (CP-4126), glutor, resveratrol, benitrobenrazide, POMHEX, GSK2837808A, GNE-140, AZD3965, XMT-1592, JPH203, SKN103, canagliflozin, nateglinide, LY345899 and DS18561882, are under development, though most lack specific antitumor effects

#### Amino acid metabolism

Cells take up glutamine, an abundant circulating non-essential amino acid via active transport through ASCT2, which is then converted to glutamate through deamination by mitochondrial glutaminases GLS1 and GLS2. Inhibitors of glutaminase, like telaglenastat (CB-839), IPN60090, cilazapril (BHV-4157), MEDI7247, and LAT1 inhibitor (JPH203) have advanced to phase I or II clinical trials (NCT04471415) in which they are being assessed in combination with sapanisertib or nivolumab for the treatment of NSCLC or other solid tumors.

Tryptophan, an essential amino acid for humans, can only be obtained through the consumption of external food sources instead of being synthesized endogenously.^675,676^ The key enzymes in its metabolism, including IDO1, IDO2, and tryptophan 2,3-dioxygenase (TDO), have attracted particular attention for their role in tumor immunomodulation.^675,677^ However, targeted therapies against IDO1, such as epacadostat,^678^ indoximod,^679^ linrodostat, and navoximod, either individually or in combination with ICIs,^530^ have not shown significant antitumor efficacy in clinical studies. Concurrently, the utilization of PROTACs targeting IDO1, IDO1 peptide vaccines, the introduction of microRNA series into CAR-T cells to suppress IDO1 expression, and the combination of IDO1 inhibitors with IDO1 vaccines, demonstrate promising avenues for cancer therapy. In addition to clearing kynurenine (Kyn), employing small molecule antagonists to inhibit the binding of Kyn to its receptor, AhR (aromatase receptor), and the subsequent activation of downstream signaling pathways represents another strategy for upregulating antitumor immunity.^530^ This approach, used either alone or in conjunction with ICIs, is currently being evaluated in early-stage trials for patients with advanced cancers. To summarize, at present the journey towards developing inhibitors targeting IDO1 (also IDO2 and TDO2),or AhR has encountered bottlenecks, and future endeavors should focus on identifying more effective inhibitors that can be used in combination with ICIs^680^ to enhance the anti-tumor efficacy.^675^

In addition to the aforementioned targets involving amino acid metabolism, studies are ongoing to develop arginine-depleting enzyme pegargiminase (ADI-PEG20) and human recombinant polyethylene glycol arginase (rhArgPEG, BCT-100, and pegzilarginase) for solid tumor patients in combination with chemotherapy or immunotherapy.

#### Lipid metabolism

While some tumors rely on fatty acid oxidation, there are currently few highly specific inhibitors available for this process. Etomoxir, which inhibits fatty acid oxidation, exhibits off-target effects, and agents like ST1326, VY-3-135, C75, AZ22, and AZ65 that target carnitine O-palmitoyltransferase 1A (CPT1A) only show preliminary antitumor activity. Following the identification of fatty acid synthase (FASN) as a potential cancer therapeutic target decades ago, agents targeting FASN, such as TVB-3664, TVB-3166, TVB-2640 and statins^681^ have been clinically investigated in various solid tumors, including NSCLC (NCT03808558), and the mature results of these studies are eagerly expected.

To sum up, while the study of cellular metabolism has a history spanning over a century, forming the foundation of modern biology and intertwined with the development of chemotherapy,^672^ overall progress in solid tumors such as NSCLC has not been as successful as the explosive developments in targeted and immunotherapies in recent years.^682^ The possible reasons are, firstly, that in the absence of specific metabolic mutations, there are almost no reliable metabolic features to distinguish cancer cells from normal cells, and high nutrient intake is not limited to cancer cells, instead, immune cells may absorb more glucose than cancer cells, leading to the widespread drug toxicity. Secondly, metabolic dysregulations rarely occur in isolation, underscoring the complexity and adaptability of metabolic networks supporting cancer growth which is driven by numerous input and output nodes across pathways^683^ involved in ATP production, electron transfer, one-carbon unit transfer, and ion channel regulation, as well as nucleotide metabolism. Therefore, this complexity implies that no single metabolic intervention can achieve a sustained effect. Thirdly, some metabolic enzymes possess moonlighting functions beyond their traditional catalytic activities, participating in the activation of oncogenic pathways.^531^

### Directly targeting host macro-environmental factors

A more ecological perspective on cancer posits that factors such as nutritional status,^684^ dietary habits, endocrine and neural networks, as well as microbiome status embodied as bacterial, viral, and even fungal infections, all influence the incidence of cancer and the efficacy of immunotherapies,^685^ whereas the foundational mechanisms still require further investigation.

#### Microbial communities and cancer

The association between the human microbiome and cancer was observed as far back as 4000 years ago, yet until the early 20th century, the realization dawned that localized infections could sometimes lead to tumor regression – the genesis of modern antitumor immunology. Through whole-genome sequencing of fecal samples, a study revealed that the presence of the gram-negative commensal bacterium *Akkermansia muciniphila* is associated with favorable clinical outcomes in patients with advanced NSCLC. The prevalence of gut mucus protozoa (specifically, the predominant SGB9226, detected in 39% of patients) accurately predicts the clinical benefits from first-line or second-line treatment,^686,687^ and even neoadjuvant^688^ therapy with anti-PD-(L)1 antibodies, independent of other clinical prognostic factors. However, there may be overlaps between beneficial and harmful bacterial groups in specific types of cancer. On the other hand, compared to controls, NSCLC patients receiving ICIs after antibiotic exposure showed shorter PFS and OS.^689^ Recently, a comprehensive analysis across various solid tumor types has shown the presence of intracellular or intratumoral microbiomes which are ubiquitous in tumors with varying components and abundances,^690^ and could enhance the efficacy of immunotherapies.^691^ However, much remains unknown about the origin and exact role of the microbiome within the TME.

#### Biologic therapeutic supplements

Given the direct relationship between microbes, cancer, and the immune system, ameliorating cancer immunotherapy outcomes through fecal microbiota transplantation (FMT) is a consequent approach – not a new concept,^692,693^ tracing back to ancient Chinese methods for treating diarrhea during the 4th century BC.^691^ While the combination of FMT and anti-PD-1 therapy has been shown to improve immunotherapy resistance in single-center phase I and II melanoma clinical trials, FMT in cancer is still in its early stages, and whether the conclusions from FMT in melanoma can be extended to NSCLC^694^ or other solid tumors is still an unknown field that needs to be confirmed. Recently, a microbial consortium named the microbial ecosystem therapeutic 4 (MET4) was developed and utilized in the MET4-IO phase II-III trials as an alternative to FMT.^695^ Probiotic supplementation has also garnered considerable public attention, with numerous clinical trials attempting to demonstrate the benefits of probiotics,^696^ yet many studies have shown that probiotics do not confer benefits, possibly due to low overall α-diversity of the microbiome.^697^ A recent study indicated that following FMT in patients with ICIs-resistant NSCLC, oral supplementation of castalagin supports anti-PD-1 activity,^698^ however, further validation is required with larger prospective cohorts. Other next-generation probiotics are being explored as adjunctive antitumor therapies.^699^ However, when chemotherapy and immunotherapy combinations, emphasizing the impacts of chemotherapy on the proliferation of viable microbiota and immunotherapy outcomes^700–702^ is imperative.

While over a hundred clinical trials are currently underway aiming to harness the microbiome to improve cancer treatment, progress towards clinical implementation has been limited, possibly due to the variations in DNA extraction and standardized sequencing approaches, or methodologies for handling sequencing contaminants across different studies.^703–705^ In addition, a majority of evidences coming from cross-sectional studies, suggested a pressing need for more prospective, longitudinal, and well-controlled human studies to confirm the outcomes of using the microbiome as an intervention.^706^

#### Strategies to increase oxygen supply, modulate the nervous system, and relieve pain

The immunosuppressive barrier in the TME, driven by potent biochemical hypoxia/HIF-1α/adenosine/A2AR pathways, poses a significant challenge to the efficacy of most known T-cell and NK cell-based cancer immunotherapies.^707^ The primary mechanism regulating hypoxia was discovered at the end of the 1990s and was awarded the Nobel Prize in Physiology or Medicine in 2019, primarily for the discovery of HIF, a critical transcription factor composed of three HIF-α subunits (HIF-1α, HIF-2α, and HIF-3α) and one HIF-1β subunit (also known as ARNT). As of now, belzutifan, a specific inhibitor of HIF-2α, received FDA approval in August 2021 for the treatment of patients with VHL-associated tumors. Currently, belzutifan in combination with pembrolizumab and lenvatinib is being evaluated in advanced solid tumors, with preliminary results indicating no additional toxicity.^708^ Other compounds targeting HIF-2α, such as PT2385, PT2399, Tempo, acriflavin, BAY 87-2243, CCS 1477, 2-methoxyestradiol, and PX-478, as well as numerous compounds targeting HIF-1α mRNA expression or influencing protein synthesis, stability and heterodimer activation, are under investigation,^709^ however, most have not been successful.

Consequently, an alternative approach to eliminate tumors has been considered, involving the direct supplement of oxygen and oxidants, which are expected to increase pro-inflammatory cytokines and chemokines (such as IL-2, IL-12 and IFN-γ) and instead reduce immunosuppressive components (such as TGF-β or T_reg_ cells), and counter or reverse hypoxia/HIF-1α-adenosine-induced immunosuppression. However, high concentrations of oxygen (>60%) can lead to severe side effects. Fortunately, oxygen-carrying nanoparticles^710^ offer several advantages in increasing local oxygen levels in tumors, yet clinical benefits have not been significantly enhanced. This may be due to the inadequate targeting and penetration properties of nanomaterials, as well as the premature release of oxygen from nanotherapeutics in circulation, potentially causing toxicity to normal cells in other organs. Moreover, metal-based nanoplatforms used for delivering and generating oxygen, such as manganese, cerium, copper, and iron, might accumulate within the body non-specifically, posing toxicity to normal tissues and organs, especially if over extended periods (≥6 months).

Recent evidence suggests that interactions among immune cells, tumor cells, and neurons or neural components, as well as even the microbiome,^711^ can control the occurrence, progression, and metastasis of cancer, and also influence therapeutic resistance.^712^ In fact, cancer cells can invade neurons within the TME through a process called perineural invasion (PNI), which is a pathway for tumor dissemination involving complex signaling interactions among neurons, cancer cells, immune cells, and Schwann cells. The resulting nerve damage activates regeneration and repair programs in Schwann cells and the nerves themselves, including the release of cytokines, such as IL-1, IL-6, IL-10, and TGF-β. Downstream immune responses include the recruitment of macrophages that secrete growth factors, which not only enhance cancer cell migration along nerves and remodel the extracellular matrix to favor PNI, but also negatively impact the global tumor immune microenvironment.^713,714^ On the contrary, cholinergic parasympathetic signals can inhibit tumor development, suggesting that the role of specific neuronal types or neurotransmitters may vary depending on the characteristics of the TME.^713^ Local neurotransmitter, like γ-aminobutyric acid (GABA) can promote tumor inflammation, thereby further affecting the efficacy of antitumor immunotherapy.^715^ Thus, the inhibition of the GABA signal is associated with higher infiltration of CD4^+^ and CD8^+^T cells, as well as CD103^+^DCs, and better tumor control.^716^ The nervous system also regulates the functions of the immune system at a systemic level, including the migration and function of immune cells. Similarly, the immune system is referred to as the seventh sense, interacting with the sensory nervous system through common regulatory molecules and receptors. Cancer pain, one of the most prevalent neurological symptoms in patients with advanced lung cancer, is a multidimensional experience involving components such as sensory discrimination, emotional motivation, and cognitive assessment. Pain relief has been shown to enhance anti-cancer effects related to the immune system.^717^ Consequently, drugs typically used to improve sleep, nausea, anxiety, and depression, particularly those that regulate pain neurotransmitters and neurocognition, should be combined with cancer immunotherapies.^516^ However, cancer-neuro-immunological science is still in its infancy, thus to map out the interaction networks and connectomes among the three at multiple scales and levels is an eager research field in the future.^516^

### Opportunities for traditional Chinese medicine in the era of immunotherapy

Traditional Chinese medicine (TCM), with over 3000 years of history, is highly favored by both cancer patients and clinicians in China, where almost all cancer patients receive TCM treatments,^718^ moreover, TCM practitioners are integral members of the multidisciplinary team (MDT).^718^ The Yin-Yang theory in TCM not only resembles Western medical immunological characteristics completely, but also uniquely elucidates a holistic treatment principle that aligns with the Western medical perspective that cancer is a systemic disease – involving interactions among different organ systems participating in the immunotherapy response.^719^

Currently, numerous studies have analyzed and validated the effects of single herbal compounds or ingredients in TCM, including phenolic compounds (such as resveratrol), polysaccharides (such as astragalus polysaccharides and ganoderma polysaccharides), flavonoids (such as epigallocatechin gallate), saponins (such as ginsenosides), terpenoids (such as Rg-III), and alkaloids (such as scopoletin), and further revealed that they not only possess antitumor activity, but also enhance the immunogenicity of vaccines, increase infiltration and function of cytotoxic T cells, and promote the potential for tumor-suppressing immune reactions through the downregulation of PD-1/PD-L1 on immune cells.^720^ Distinctly, TCM also acts to inhibit the immune system through its anti-inflammatory properties, enhancing the safety of immunotherapies,^721^ leading to a decoupling of toxicity and efficacy, this has yet to be substantiated clinically.^722^ Recent studies have also highlighted that TCM can modulate the systemic response to ICIs by regulating crosstalk between gut microbiota and immune cells.^722^

In summary, the advent of immunotherapy represents a new opportunity for TCM to fully harness its potential – a supportive and auxiliary role rather than a direct leadership one, despite the fact that most of the insights are preliminary clinical conclusions or from non-randomized controlled studies. Recently, AI technologies have been applied to validate the scientific validity and efficacy of TCM, potentially addressing the shortcomings of subjectivity, vagueness, and complexity in TCM application processes.^723^ Therefore, developing a clinical research framework tailored to the intrinsic characteristics of TCM, or integrating TCM with Western medicine seamlessly, makes sense, although this formidable task may not be easily achieved in the short term.

## Translational research in NSCLC

Facing the complex and multi-dimensional nature of NSCLC biology,^724^ which spans a vast breadth and scope encompassing molecular, cellular and tissue pathology, involving tumor formation and treatment response, as well as a plethora of “big data” evidence derived from the utilization of sophisticated experimental methodologies and computing tools,^725^ a key priority for the oncology community is to concentrate on how to translate a drug from target identification into clinical practice, along with optimizing its application. Undoubtedly, this does not imply that other crucial areas are dispensable for the eradication of cancer cells,^726^ as mainly considering limited space of this article.

### Discovery of new drug targets

Since the approval of imatinib in 2001, more than 90 small-molecule targeted drugs have been used to treat various cancers,^727^ yet they only cover approximately 7% of patients. Consequently, for a broader population of NSCLC patients,the rapid and efficient development of novel drugs is need.^728^

Drug development often employs simple strategies that might involve repeating or iterating through currently validated targets, such as EGFR or ALK,^729^ or traditional undruggable targets like KRAS, P53, and MYC mutations to achieve novel clinical validation.^730^ Most importantly, leveraging large datasets from the latest human molecular genetics for computational biology analysis can help identify previously unvalidated novel targets.^108,731^

With the improvement and application of DNA and RNA sequencing technologies, such as whole genome and whole exome sequencing,^134,732^ genomic sequence abnormalities occurred in protein kinases^733^ or transcriptional regulatory molecules, etc., have been systematically identified and described in hundreds of thousands of patient samples from over 30 different cancer types, including substitutions, insertions, deletions, fusions or splicing, copy number changes, and complex chromatin structural variations in coding or non-coding regions.^734–736^ Moreover, through RNA interference (RNAi) techniques such as siRNA and short hairpin RNA (shRNA), the phenotypic consequences of downregulating a single gene in cancer cells can be characterized^737^ – unfortunately producing many false positive. However, currently, CRISPR-Cas9 gene editing tools^738^ can be used to induce specific genetic changes and have been shown to have fewer overall off-target effects than RNAi^739^ in uncovering novel resistance targets for drug development. The endeavor above results in novel cancer targets being discovered, such as the NRG1 or NRG2 fusion, RAS-GRF1 fusion,^740^ CLIP1-LTK fusion,^741^ UBA1 mutation,^742^ RICTOR, PINK1, L1RE1, ILF2 mutation and SBS40a or SBS22a mutation,^743^ as well as ATM (aurora kinase)-ATR (ataxia telangiectasia and rad3-related protein)-CHEK1 (checkpoint kinase 1), all of which will feature in the next step that drug development will take.

The unveiling of synthetic lethality relationships (the initial focus was primarily limited to DNA repair pathways), achieved through combined sequencing and functional genomics endeavors,^739^ represents a significant advancement, leading to the discovery and application of numerous novel therapeutic targets.^744^ For instance, Brg1/Brm-associated factor (BAF) is part of the SWI/SNF chromatin remodeling complex, featuring subunits such as SMARCA4, ARID1A, and SMARCB1, representing another class of genes frequently lost or mutated in cancer.^745^ These genes are commonly found in more than 20% of human cancers. Currently, several inhibitor formulations targeting SMARCA4 mutations and SMARCB1 loss are undergoing clinical trials.^746^

What needs to be pointed out is that while certain oncogene dependencies can indeed be explained by specific genetic mutations, the relationship between them is non-linear, and interactions among various genetic alterations often determine the degree of dependence on individual genes.^477^ Furthermore, other non-genetic factors may also contribute to selective dependencies, encompassing changes in the epigenome, transcriptome, proteome of cancer cells, along with their microenvironment. In short, addressing cancer survival dependencies often necessitates interventions against multiple genetic variants – target inhibitions.^272^

### Pharmaceutical chemistry techniques for drug development

While identifying novel, biology-dependent targets during drug discovery efforts is a priority, perhaps of equal importance is achieving a drug with high therapeutic index – a composite parameter reflecting the selectivity of drug, the specificity of target binding and the off-target toxicity, exemplified by third-generation EGFR and ALK inhibitors.

Among tumor-related protein targets, 85% are considered “undruggable” due to lack of clear binding pockets, well-defined ligands or substrates with excessively high-affinity, such as mutations in P53 and RAS genes. Following closely are numerous cancer drivers, including transcription factors, which cannot be targeted by current therapies owing to their inability to be expressed on the cell surface.^747^ Consequently, the field of medicinal chemistry needs to transcend the “Rule of 5” proposed in 1997 – which stipulates that hydrogen bond donors should be ≤5, hydrogen bond acceptors ≤10, molecular weight ≤500, and logP (indicative of hydrophobicity) ≤5 for compounds – and expand to encompass other crucial drug attributes and safety considerations.^748^ Specifically, compounds obtained through macrocyclization contain more hydrogen bond donors and acceptors, enabling tighter binding to difficult-to-bind pockets while maintaining cellular permeability and oral bioavailability. Secondly, allosteric ligands bind to sites distinct from the active site or ortho site, modulating protein activity, for example, SHP099 inhibits the SHP2 phosphatase via interaction with an allosteric site. However, identifying these sites within targets poses a significant challenge, especially for those that might be cryptic, and are only revealed under specific protein conformations. Thirdly, fragment-based drug discovery (FBDD) has emerged as a widely adopted strategy in the field of drug discovery, represented by the BRAF inhibitor, vemurafenib, which was the first approved drug discovered with fragment-based approaches. A notable advancement is the rational design of covalent small molecule drugs,^749^ which has led to over 40 covalent drugs being approved, or currently under clinical evaluation, such as RMC-6236 or RMC-7977 against both mutant (KRAS^G12X^) and wild-type KRAS, NRAS and HRAS variants.^750^ However, a common criticism of covalent compounds is their potential for off-target or non-specific toxicity due to their ability to modify non-target proteins.

Furthermore, specific degradation agents^751,752^ can be developed for proteins of interest (POI) that are difficult to develop functional inhibitors or lack traditional deep binding pockets, as well as transcription factors – that cannot be targeted by currently available therapies due to their inability to be expressed on the cell surface.^747,753^ Molecular glue degraders, such as thalidomide, are capable of forming a ternary complex with E3 ubiquitin ligases, leading to the ubiquitination of POIs and subsequent degradation by the proteasome. Other modular degraders, known as proteolysis targeting chimeras (PROTACs), which were first described in 2001,^754^ exemplified by bifunctional or heterobifunctional inhibitors,^755^ are undergoing clinical development, covering a wide range of target proteins including KRAS^G12C^, BCL-xL, BRD9, BTK, EGFR^L858R^, BRAF^V600E^, ER, AR, TRK, folate receptor,^756^ transferrin receptor 1^757^ and IRAK4.^754^ Lately, endocytosis-triggering binding proteins (EndoTags) fused to soluble or transmembrane proteins can lead to lysosomal trafficking and higher-specificity target degradation, thereby offering considerable therapeutic potential when applied to the development targeted antibody-drug and antibody-RNA conjugates.^758^

### Data science and AI application in drug development

Over the past 20 years, in the realm of oncology drugs, 68% have been small molecules, encompassing targeted therapies such as lorlatinib and osimertinib in NSCLC.^279,759,760^ Significant advancements in medicinal chemistry are underpinned by structure-based drug discovery methodologies, alongside advances in computational capabilities (including Graphics Processing Units), sophisticated software algorithms (such as those deep learning), and enhanced data accessibility. By leveraging computational tools and various databases (such as cBioPortal, COSMIC, ICGC Data Portal, UCSC Genome Browser, Genomic Data Commons, FireBrowse, OncoKB, DepMap, and canSAR.ai), researchers can conduct virtual screening, pharmacological predictability assessments, and structural refinement to uncover novel chemical entities and enhance the efficacy and selectivity of the existing small molecules, thereby minimizing unwanted side effects. The integration of tumor genetic and functional genomic datasets derived from large-scale screens has led to the development of several innovative therapeutic approaches. For instance, three BRAF inhibitors, vemurafenib, dabrafenib and encorafenib, have been approved for treating multiple tumors harboring BRAF^V600^ mutations.

Advancements in DL technology have also fueled the development of generative chemistry engines, enabling the creation of entirely new chemical structures represented through SMILES notation or molecular graphs, facilitating the discovery of more promising compounds. Protein structure prediction is a fascinating area of research, where DeepMind’s AlphaFold (awarded 2024 Nobel Prize in Chemistry) and AlphaFold2^761^ have been applied in virtual screening and free energy perturbation (FEP) However, given the large degree of dynamic flexibility in proteins, current methods for predicting protein structures still require further improvement in terms of accurately describing their dynamic changes. An alternative strategy is to leverage DL algorithms, including DrugCell,^762^ DeepSynergy, or MOLI, alongside tumor molecular phenotype data,^763^ to model in silico and forecast drug sensitivity in human cancer cells. However, these algorithms have only been used for preliminary screening for research purposes so far.

What needs to be emphasized is that in the future, we must fully integrate and collaborate on methodologies^108^ simultaneously rather than apply techniques in a linear and sequential manner to develop novel drugs with enhanced pharmacological profiles, broader therapeutic windows, improved tolerance, extended treatment durations, higher efficacy, and the ability to work synergistically with other drug classes, ultimately offering the best opportunities for NSCLC cure.^108^

### Enhancing the clinical translation of drugs

Of course, even after identifying new latent targets and devising novel approaches to overcome resistance, covering TKIs, various formats of antibodies, ADCs, cell therapies, vaccines, and OVs, substantial experimental validation and clinical trials are required. These processes not only demand considerable time and resources but also carry significant risks of failure. Therefore, establishing more efficient and accurate systems for validation and trials is essential to improve the success rate of drug development.

Modeling of in vitro drug responses represents another rapidly progressing technique for delineating tumor functionality. Beyond DL models, like DrugCell, which are capable of preliminarily pre-screening potential drug candidates,^762^ the progress in 3D culture methods based on fresh tissue samples or CTCs, like organoid cell culture – retains certain aspects of tumor genotypes and phenotypes, can also provide quick insights into the effectiveness of candidate compounds. More recently, automated platforms have enabled high-throughput drug testing on organoid cells and biopsies, also facilitating sequential drug treatment screening. Notably, using organoid models for drug testing involves timescales of several weeks to months, making their integration as a co-clinical tool poses a challenging endeavor.

Given the NSCLC research landscape, various targets existed in oncogenes or novel mutations induced by drugs are becoming increasingly rare (incidence ≤5%),^764^ suggesting under such circumstances, developing newer drugs may offer lower economic appeal for pharmaceutical companies. On the other hand, in the context of immunotherapy, collaborative efforts among diverse stakeholders for conducting combined drug treatments have yielded very few successful cases to date.^463^ Consequently, significant financial investment, particularly from government-sponsored funds, could help to address the existing two issues.

Moreover, the growing difficulty in enrolling participants harboring rare molecular subtypes in clinical trials has prompted the introduction of innovative trial methodologies, such as umbrella trials, which focus on multiple targeted therapies or immunotherapies for a singular condition, or basket trials,^765^ which assess a particular treatment’s efficacy across diverse diseases.^766^ For instance, the NCI-MATCH (Molecular Analysis for Therapy Choice) trial,^767^ which commenced in 2015 and was completed in 2023, and in which 38% of participating patients had rare or less common types of cancer, provided a portion of findings to support the accelerated approval of the combination therapy of dabrafenib plus trametinib for patients with BRAF^V600^ mutant tumors. By extension, the REFINE-Lung study utilized a design known as the multi-arm multi-stage approach focusing on continuous response interventions, which is suitable for studies aiming to optimize dosing, frequency, or duration of treatments for both new and existing drugs

Secondly, diverse sequencing analyses targeting circulating free DNA or RNA, followed by the integration of imaging data and comprehensive radiogenomic insights^185^ through machine learning techniques, are evolving into a technology for dynamic^768^ assessment. Once integrated into innovative clinical trial designs,^453^ this technology not only accelerates the drug development process but also enables a more personalized and effective treatment strategy.^769^

Thirdly, while OS remains the most valuable endpoint for assessing efficacy, composite endpoints such as PFS, DFS, recurrence-free survival (RFS), and EFS have become popular over the past few decades to increase statistical power in clinical trials and reduce the required follow-up period, thus lowering costs.^770^ However, these composite endpoints are often poorly defined and vary significantly across studies, complicating the comparison of results across trials. Moreover, using OS in clinical trials involving early NSCLC patients can be challenging, leading to the adoption of alternative endpoints such as MPR and pCR,^203^ as well as EFS^202^ or DFS. Notably, there are no phase III randomized controlled trials that test the association between these non-time-based or composite endpoints and OS, hence, longer-term follow-up data is still required.^195^

Notably, while multiple primary endpoints pose certain advantages within a particular setting in randomized controlled trials,^771^ the false discovery rate rises to 0.098 when analyzing two independent endpoints at a significance level of 0.05. As a result, it is imperative to adjust and reallocate statistical alpha threshold, such as potentially splitting the significance level from 0.05 to 0.01 and 0.04, to avoid an increased likelihood of false positives regarding the intervention’s effectiveness.

Lastly, how to optimally manage adverse effects, particularly those associated with immunotherapies, is crucial for successfully improving patient prognosis, as there is a positive correlation between the occurrence of non-fatal irAEs and the efficacy of immunotherapies. As a result, the excessive use of corticosteroids to mitigate toxicity inevitably compromises PFS and OS. Hence, the critical challenge is to decouple the relationship between side effects and efficacy – to reduce side effects without impacting therapeutic outcomes. Strategies targeting specific immune cell subsets (such as neutrophils and tissue-resident effect memory CD8 T cells) and cytokines (including IL-1, IL-6, and IFN-γ),^772,773^ as well as complement activation pathways^774^ or even FMT,^775^ might not impact the sustained antitumor immunity.

### Maximizing the utilization of real-world data

As clinical trials increasingly concentrate on rare genetic alterations and diseases, regulatory authorities have recognized that conducting large-scale randomized controlled trials might not be practical in every scenario. Instead, single-arm studies that showcase clear efficacy could potentially suffice for drug approval. Simultaneously, there has been an acknowledgment of the significance of gathering data from patients undergoing treatments outside of clinical trials^776^ to inform clinical decisions, which underscores broader real-world data (RWD) from post-approval evaluations are crucial in confirming the improvements in patient outcomes. Under the mandate of the 21st Century Cures Act passed by Congress in 2016, the FDA established the real-world evidence (RWE) initiative, which not only focuses on post-approval studies but also accelerates the approval of new indications for drugs, for instance, pemphixine, a CDK4/6 inhibitor, was recently approved for the treatment of HER-2 negative metastatic breast cancer, partly based on RWE. With RWD increasingly supplementing clinical trial data, standardization in data collection and application will be essential, and several initiatives aimed at capturing RWD have been prompted for this purpose. However, unlike the structured, easily accessible data defined within clinical trials, RWD is typically unstructured and heterogeneous, scattered across different systems including HER (Health system-scale language), medical images, tumor samples, and blood test data.^777–779^ As such, AI-driven pipelines are essential for reproducibly and transparently integrating, mining and sharing information from the cloud space^780^ containing RWD in oncology^781^ to uncover molecular patterns^782^ associated with treatment response and clinical prognosis.^783^

## Conclusions and prospects

### Prevention

For over a century, lung cancer has transitioned from a rare disease to the leading cause of cancer-related deaths among both men and women.^1^ Prevention of NSCLC based on risk factors is our advanced pursuit goal. Regarding the risk factors associated with the development of NSCLC,^600^ while the International Agency for Research on Cancer has listed several environmental factors such as arsenic, asbestos, beryllium, cadmium, chromium, smoke, diesel exhaust, nickel, silica, soot, and uranium, other factors, like hormone replacement therapy, second-hand smoke,^19^ or germline mutation,^784^ might increase the risk of lung cancer in non-smoking females, especially for early-onset disease. Moreover, how to increase the motivation of various populations to quit smoking (including electronic cigarettes^785^), ensure the acquisition of correct cancer prevention knowledge,^786^ and improve screening methods for high-risk lung cancer populations represents an unmet area that requires further clarification.^20^

### Therapies

In the past two decades, surgical interventions for NSCLC have shown sustained progress,^192^ as evidenced by a historical narrative tracking the gradual transition from initial traditional open thoracic surgery to minimally invasive VATS, and more recently, the single-port thoracoscopy assisted by the Da Vinci surgical system. In the future, AI will be widely applied in the field of NSCLC surgery. Prior to the operation, AI not only detects and categorizes lung nodules^787^ but also conducts comprehensive surgical risk assessments. During the operation, AI seamlessly integrates preoperative and intraoperative imaging data through leveraging on a range of indicators such as radiological, fluorescent, magnetic, and hybrid options to provide real-time guidance, achieving unparalleled accuracy in identifying tumor boundaries that surpasses human visual capabilities. Post-operation, AI facilitates pathology analyses, manages complications, and assists in building predictive models to enhance personalized treatment decisions, ultimately achieving improved patient outcomes.

Thanks to transformative advancements in fields such as computer technology (like digital twins or digital connectivity), robotics, particle therapy,^395,788^ and FLASH radiotherapy, as well as the integration of MRI (MR Linac) or PET signals, radiotherapy has significantly revolutionized the capabilities of accurate dose computation and geometric localization,^789^ however, midtreatment PET-adapted RT dose escalation

did not improve efficacy outcomes.^790^ In parallel, the understanding of cancer biology and radiobiology continues to advance, contributing collectively to increased long-term survival rates for cancer patients. In the short term ahead, the application of data-driven AI algorithms in radiotherapy will further unfold exceptional capabilities across multiple dimensions, notably in tumors and normal tissues segmentation, dose optimization, prognosis and toxicity prediction, and quality assurance,^791^ even along with enhancing communication between healthcare providers and patients.

In 1948, David Karnofsky and his colleagues published an article on the treatment of lung cancer with nitrogen mustard,^8^ marking the first appearance of chemotherapy in the field of lung cancer research. Following the dawn of the new millennium, NSCLC was recognized as a disease characterized by significant molecular pathologic heterogeneity, leading to a ground-breaking therapeutic landscape that evolved towards stratification strategies based on tumor genetics and immunobiological markers. The former can indicate the precise targeting for targeted therapy,^481^ while the latter can imply the degree of benefit of ICIs.^32,792^ Over the past five years, treatments based on TKIs and ICIs have increasingly been incorporated into the management of early-stage NSCLC, primarily aiming to enhance cure rates post-surgery or after radiotherapy.

In the near future, apart from expanding the arsenal of drug treatments, there has been a growing anticipation of synergies among different therapeutic approaches, where ICIs are utilized in tandem with chemotherapy, anti-angiogenic treatments, allogeneic CAR-T cell therapies, customized vaccines,^793^ T cell engagers,^794^ and ADCs, among other methodologies.^795^ However, regarding immunotherapy, a more dialectical perspective may be necessary, which recognizes that tissue-resident lymphocytes, such as T_reg_s, can facilitate timely wound healing and tissue repair following cancer cell lysis, as well as mitigate excessive immune responses in normal tissues – thereby reducing treatment side effects. Thus, a outstandingimmunotherapy should be have diverse capabilities in a coordinated manner, ultimately restoring tissue homeostasis.^796^ Simultaneously, establishing molecular tumor boards (MTBs) is therefore crucial for developing frameworks to decipher rare or complex mutation events, facilitating evidence-based treatment decision-making processes.

### Other considerations

Over the past decade, oncologists worldwide have witnessed an unprecedented acceleration in drug development and regulatory approvals, yet they have also become acutely aware of the disparities in accessing these therapies and in participating in clinical trials,^457^ leading to significant survival differences among cancer patients.^797^ As a result, increased government investment in healthcare, encouragement of charitable donations, and promotion of public-private partnerships are essential. Meanwhile, in addition to comprehensive medical insurance,^316^ healthcare providers, especially doctors, regardless of the situation, choose a reasonable and cost-effective treatment plan is indispensable.^798^

Nowadays, cancer treatment based on the organ where the tumor originated is now increasingly disconnected from the advancements in precision medicine – which uses the molecular characteristics of tumors and immune cells to guide treatment. However, exhibiting similar therapeutic responses across cancers of different tissue origins, which share common drivers, is not universal rules.^473^ Therefore, considering the existing financial constraints and technological limitations, a holistic molecular profiling approach that integrates epigenetics, transcriptomics, and proteomics analysis, while complementing rather than superseding traditional histopathological classifications, appears to be the most prudent course of action. Moreover, notably, AI models^799^ or innovative foundational modeling technologies merely represent the most recent iterations within the probabilistic model continuum that informs medical decision-making, therefore, the ultimate value judgment should always be independently held by the designers and users of these models, in other words, the core position of human values and ethical deliberations within the decision-making process should remain unaltered.^800^

## Conclusions

In the last two decades, the panorama in our eyes encompasses outstanding advancements in NSCLC epidemiological studies, molecular pathology exploration and diverse treatment modalities, with a particular emphasis on drug therapies, all of which have shed light on the promise of improving survival rates for a progressively larger group of patients.^31^ However, the persistence of inevitable drug resistance poses a substantial hurdle, leading to the rarity of durable responses.^481^

**Fig. 13:**
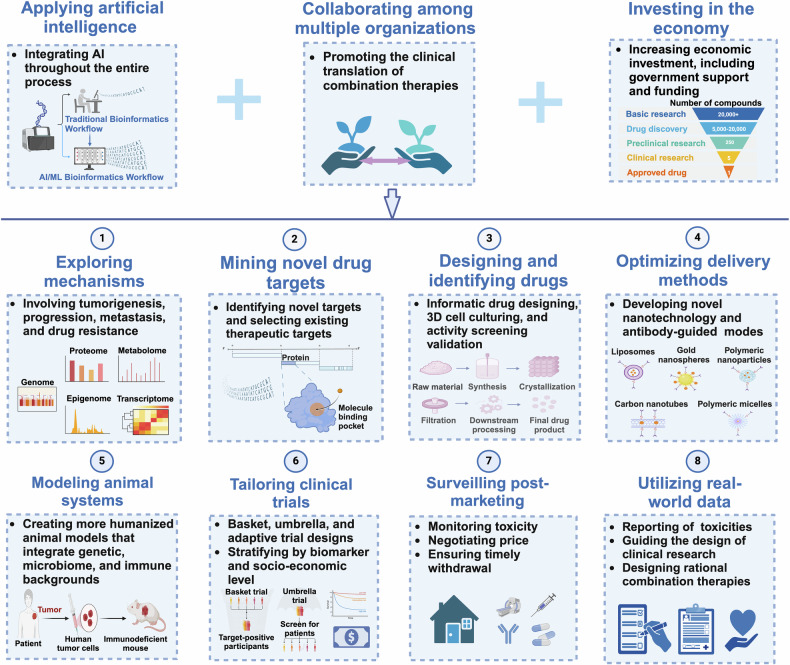
Flowchart for attaining optimal drug treatment efficacy in NSCLC. This flowchart represents an ideal scenario, and not all drugs or treatments will necessarily follow this development process. The exploration of mechanisms is the most critical and fundamental step. Without this, subsequent steps might merely be illusory. The battle against NSCLC requires close collaboration among various teams, which involves searching for precise internal markers within seemingly random surface events and strategically allocating dominant and cooperative roles in combination therapy to ultimately conquer this formidable disease

Looking ahead, the ideal research process, under AI management throughout, is comprised of, employing multi-omics profiling to deepen our understanding of NSCLC biology, designing high-index therapeutics, and then utilizing humanized animal models for screening,^108^ ultimately conducting biomarker-guided,^801^ combination therapy-focused clinical^463^ trials, by which the most precise treatment^468^ for NSCLC will be achieved, in optimistic estimation (Fig. 13).
